# Efficient model selection for predictive pattern mining model by safe pattern pruning

**DOI:** 10.1016/j.patter.2023.100890

**Published:** 2023-12-01

**Authors:** Takumi Yoshida, Hiroyuki Hanada, Kazuya Nakagawa, Kouichi Taji, Koji Tsuda, Ichiro Takeuchi

**Affiliations:** 1 Department of Engineering, Nagoya Institute of Technology, Nagoya, Aichi 466-8555, Japan; 2 Center for Advanced Intelligence Project, RIKEN, Tokyo 103-0027, Japan; 3 Department of Mechanical Systems Engineering, Nagoya University, Nagoya, Aichi 464-8603, Japan; 4 Department of Bioinformatics and Systems Biology, The University of Tokyo, Bunkyo-ku, Tokyo 113-0033, Japan

**Keywords:** predictive pattern mining, item set mining, sequence mining, graph mining, sparse learning, safe screening, convex optimization

## Abstract

Predictive pattern mining is an approach used to construct prediction models when the input is represented by structured data, such as sets, graphs, and sequences. The main idea behind predictive pattern mining is to build a prediction model by considering unified inconsistent notation sub-structures, such as subsets, subgraphs, and subsequences (referred to as patterns), present in the structured data as features of the model. The primary challenge in predictive pattern mining lies in the exponential growth of the number of patterns with the complexity of the structured data. In this study, we propose the safe pattern pruning method to address the explosion of pattern numbers in predictive pattern mining. We also discuss how it can be effectively employed throughout the entire model building process in practical data analysis. To demonstrate the effectiveness of the proposed method, we conduct numerical experiments on regression and classification problems involving sets, graphs, and sequences.

## Highlights

• Predictive pattern mining is used for interpretable modeling of complex structures
• Safe pattern pruning (SPP) can solve this problem efficiently
• Utilizing multiple feasible solutions reduces further computational costs of SPP

## The bigger picture

Data-driven approaches to predicting properties or classifying instances represented as complex structures (e.g., sequences and graphs) are highly important. For example, the relationship between organic molecules and their chemical properties can be modeled using graphs as inputs, where nodes represent atoms and edges represent chemical bonds. Similarly, the classification of proteins is achieved by models that predict class probabilities from sequences of amino acid residues. While deep neural networks have been widely studied recently, they often lack explainability; that is, they are difficult to interpret and monitor by humans. Thus, the development of more interpretable and resource efficient methods for such data remains an essential challenge.

## Graphical abstract

**Figure:**
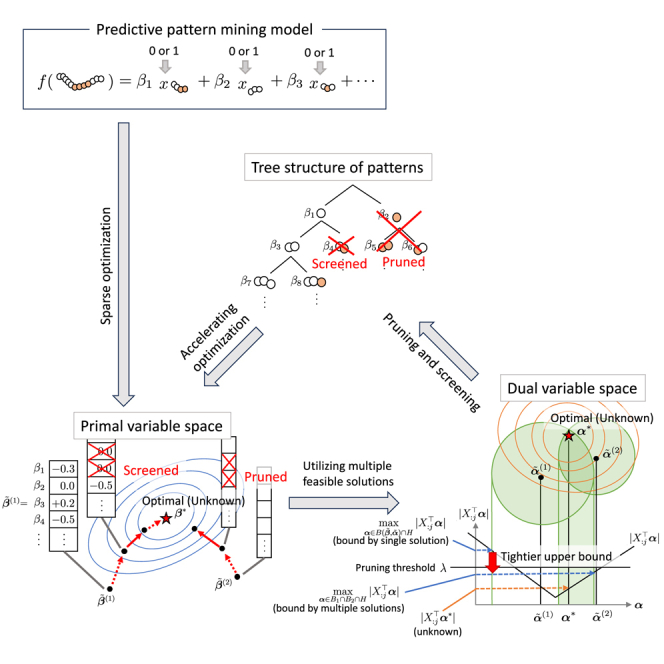

## Teaser

Predictive pattern mining is an approach to modeling the relationship between complex structures such as amino acid sequences or molecular graphs and their numerical properties or class labels using their unified inconsistent notation sub-structures. Yoshida et al. present a method (safe pattern pruning [SPP]) that uses safe screening and the tree structure of patterns. SPP overcomes the computational drawback of existing strategies and reduces further computation costs by utilizing multiple feasible solutions from the optimization problem for obtaining the predictive model.

## Introduction

In various practical problems, it is necessary to handle structure data such as sets, graphs, and sequences. For example, in the field of life sciences, interactions between different genes are represented as sets, drug-able chemical compounds are represented as graphs, and amino acid sequences that make up proteins are represented as sequence data. In this paper, we consider prediction problems such as regression and classification when the input is structure data. In the aforementioned cases, problems such as predicting the presence or absence of a disease based on interactions between genes, predicting the effectiveness of drugs based on chemical compound structures, and predicting allergic reactions based on the amino acid sequences of food proteins are examples of our target applications. In predictive modeling for structure data, the challenge is how to represent the structure data so that they can be fed into machine learning framework. In this paper, we consider a class of machine learning models called predictive pattern mining.^1^^,^^2^^,^^3^^,^^4^^,^^5^^,^^6^^,^^7^^,^^8^^,^^9^^,^^10^^,^^11^^,^^12^^,^^13^^,^^14^

There are mainly three types of machine learning approaches for structure data. The first approach is the kernel-based approach. In this approach, a kernel function that can quantify similarities between sets, graphs, sequences, and other structures are introduced, and it is used together with kernel-based machine learning methods such as support vector machines and Gaussian processes. A variety of kernel functions specialized for each type of structures have been proposed and used in practical problems.^15^^,^^16^^,^^17^^,^^18^^,^^19^^,^^20^^,^^21^ The second approach is deep learning-based approach. This approach employs neural network models with special types of input and hidden layers that are designed to handle the inputs in the form of sets, graphs, sequences, and other structures. For example, PointNet is a neural network especially developed for set data,^22^^,^^23^ graph neural networks are used for graph data,^24^^,^^25^^,^^26^^,^^27^^,^^28^ and there are many neural network architectures for sequence data such as recurrent neural networks and LSTM.^29^^,^^30^^,^^31^^,^^32^ These two approaches are effective when we are only interested in prediction. In many practical problems, however, simply having good predictive performance is not enough. In prediction modeling for structure data, knowledge extraction such as identifying the sub-structures that contribute to the prediction, is required for the explanation and interpretation.

**Figure 1:**
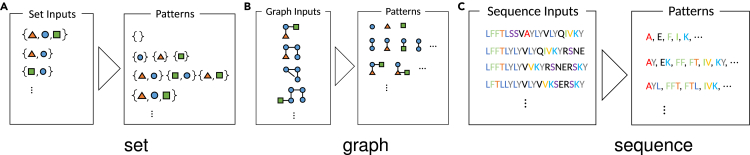
Examples of patterns for each type of structure Examples of patterns (sub-structures) of (A) set data, (B) graph data, and (C) sequence data.

The third approach is predictive pattern mining, which is the subject of this paper. In contrast with the two aforementioned approaches, knowledge extraction is possible in a predictive pattern mining approach. A common feature among many types of structure data such as sets, graphs, and sequences is that they can be decomposed into sub-structures. For example, considering a set of three genes $\left\{g_{A},g_{B},g_{C}\right\}$ as a set data, it has the following sub-structures:

$$\emptyset \text{,}\left\{g_{A}\right\},\left\{g_{B}\right\},\left\{g_{C}\right\},\left\{g_{A},g_{B}\right\},\left\{g_{A},g_{C}\right\},\left\{g_{B},g_{C}\right\},\left\{g_{A},g_{B},g_{C}\right\}\text{,}$$

where ∅ is the empty set. If we consider a predictive model that takes a set as an input, it is possible to extract knowledge by knowing which sub-structures contribute significantly to the prediction. In this paper, these unified inconsistent notation sub-structures are called patterns. Figure 1 shows examples of patterns for set, graph, and sequence data.

**Figure 2:**
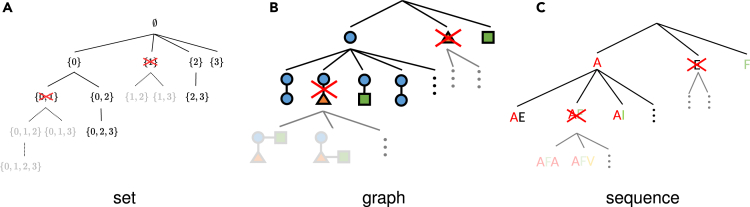
Pruning in the search for patterns of each type of structure Conceptual diagram of pruning in the search for patterns of (A) sets, (B) graphs, and (C) sequences represented by a tree.

The difficulty in predictive pattern mining lies in the computational complexity of efficiently handling an exponentially increasing number of patterns. In any of the set, graph, or sequence structure data discussed so far, the number of all possible sub-structures (patterns) are huge, making it difficult to consider a machine learning model that naively treats all possible patterns as features. Since the patterns that affect predictions are often only a small part of the vast number of pattern features, the basic strategy for predictive pattern mining is to efficiently identify relevant pattern features and remove irrelevant pattern features that do not affect predictions. In the field of pattern mining, algorithms utilizing the fact that patterns can be represented in a tree structure have been exploited for tasks such as enumerating frequently occurring patterns (Figure 2). Our main contribution in this paper is to propose a method for efficiently finding patterns that significantly contribute to predictions by using the tree representation of patterns, similar to other pattern mining methods.

**Figure 3:**
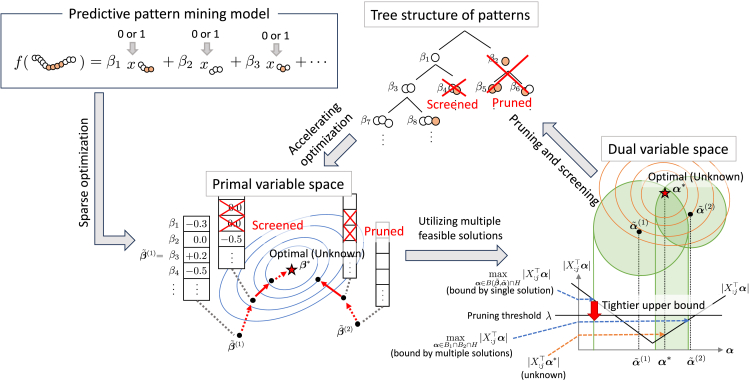
Overview of our method It aims to solve predictive pattern mining problems utilizing safe screening. We enhanced the screening performance by exploiting the tree structure of patterns and the multiple feasible solutions of the optimization problem.

To this end, we propose a technique called safe pattern pruning (SPP) by combining safe screening, which has been developed in the field of sparse modeling, and pattern mining that utilizes tree-based pattern representations. We show the overview of our method in Figure 3. In the SPP method, we consider the sparse estimation of linear models that can have any pattern as a feature for predictive pattern mining model and identify a specific set of patterns where the coefficients become zero in the optimal solution.

This paper is an extended version of the preliminary conference proceeding presented by a part of the authors.^33^ The entire content of “SPP for model selection” is an addition to this paper, and a significant portion of the experiments described in “Numerical experiments” were newly conducted in this paper. In this paper, we newly introduce methods to effectively perform model selection by effectively using the SPP; provide software that can comprehensively handle regression and classification problems with sets, graphs, and sequences; and present new numerical experimental results.

## Results

### Notations

We use the following notations in the rest of the paper. For any natural number n, we define [n]:={1,…,n}. For a matrix $Z\in R^{n\times m}$, its i-th row and j-th column are denoted as $Z_{i:}$ and $Z_{:j}$ , respectively, for i∈[n] and j∈[m]. The $L_{1}$ norm, the $L_{2}$ norm of a vector $v\in R^{n}$ are defined as ${\left\|v\right\|}_{1}:=\sum_{i\in \left[n\right]}\left|v_{i}\right|$ and ${\left\|v\right\|}_{2}:=\sqrt{\sum_{i\in \left[n\right]}{\left|v_{i}\right|}^{2}}$ , respectively. In addition, the symbols 0 and 1 denote vectors of suitable dimensionality, where all of their elements are equal to 0 and 1, respectively.

### Problem setup: Predictive pattern mining

Let S be the input space for structure data, e.g., S is a collection of sets, graphs, and sequences. We assume that a certain partial order ⊏ on S is defined, which represents an inclusion relationship. For example, in the case of subset mining, we can use well known subset operator ⊂ as ⊏. Let Y be the space of response (i.e., output space). We consider Y⊆R for regression problems and Y={−1,+1} for binary classification problems.

We denote the training dataset with n instances as $D={\left\{\left(S_{i},y_{i}\right)\right\}}_{i\in \left[n\right]}$, where $S_{i}\;\in S$ and $y_{i}\;\in Y$ are the pair of structure input and response for the i-th training instance. Let P be the set of all sub-structures (patterns) contained in ${\left\{S_{i}\right\}}_{i\in \left[n\right]}$ and $p_{1},p_{2},\ldots \text{,}p_{d}\;\in P$ be the elements of P. Here, d is the total number of patterns, which increases exponentially with the complexity of structure inputs. In predictive pattern mining, we consider a generalized linear model in the form of

(Equation 1) $$g\left(y_{i}\right)=\beta_{0}+\beta_{1}x_{i1}+\beta_{2}x_{i2}+\cdots +\beta_{d}x_{id},i\in \left[n\right]\text{,}$$

where $x_{ij}$ equals 1 if the i-th input structure has the j-th pattern and 0 otherwise, $\beta_{0}$ and $\beta_{j}\;\in R\text{,}j\in \left[d\right]$ are the model coefficient, and g is the link function for generalized linear model. With this notation, the training set for the predictive pattern mining is also represented as (X,y), where $X\in {\left\{0,1\right\}}^{n\times \left(d+1\right)}$ is the matrix with the (i,j)-th element $x_{ij}$ for j∈[n] and 0-th column as the intercept, i.e., $x_{i0}=1$ , and $y\in R^{n}$ is the vector with $y_{i}$ being the i-th response. Furthermore, we define a vector of coefficients as $\beta =\left[\beta_{0},\beta_{1},\ldots ,\beta_{d}\right]\in R^{d+1}$. Then, the linear model in Equation 1 is simply written as g(y)=Xβ, where g is assumed to be applied to y in an element-wise manner.

The goal of predictive pattern mining is to find the vector of coefficients parameters $\beta \in R^{d+1}$ that minimizes the following class of loss function:

(Equation 2) $$\begin{array}{l} \beta :=\underset{\beta \in {\mathbb{R}}^{d+1}}{\mathrm{argmin}}\;P\left(\beta \right)\text{,}\\ P\left(\beta \right):=L_{y}\left(X\beta \right)+\Omega_{\left(\lambda ,\kappa \right)}\left(\beta \right)\text{,} \end{array}$$

where $L_{y}:R^{n}\to R$ is a convex loss function with Lipschitz continuous gradient, and $\Omega_{\left(\lambda ,\kappa \right)}$ is a convex regularization function. In this paper, we focus on the following regularization function as $\Omega_{\left(\lambda ,\kappa \right)}$, which is called elastic net regularization^34^:

(Equation 3) $$\Omega_{\left(\lambda ,\kappa \right)}\left(\beta \right)=\lambda \sum_{j\in \left[d\right]}\left(\left|\beta_{j}\right|+\frac{\kappa }{2}\beta_{j}^{2}\right)\text{,}$$

where λ>0 and κ≥0 are the hyperparameters for tuning the strength of regularization. Note that we assume that the intercept $\beta_{0}$ of β is not regularized. When using this regularization function, a sparse solution is obtained, meaning that many coefficients $\beta_{j}^{*}$ shrink to zero in the optimal solution.

The dual problem of Equation 2, introduced by Fenchel’s duality theorem (see Corollary 31.2.1 of Rockafellar,^35^ for example), is written as

(Equation 4) $$\begin{array}{l} \alpha^{*}:=\max_{\alpha \in R^{n}}\;D\left(\alpha \right)\text{,}\\ D\left(\alpha \right):=-L_{y}^{*}\left(-\alpha \right)-\Omega_{\left(\lambda ,\kappa \right)}^{*}\left(X^{⊤}\alpha \right)\text{,} \end{array}$$

where $f^{*}$ indicates the convex conjugate of a convex function f, which is defined as follows.

Definition 1 (convex conjugate): Let $f:R^{n}\to R$ be a convex function, the convex conjugate $f^{*}:R^{n}\to R$ is defined as

$$f^{*}\left(v\right)=\underset{u\in R^{n}}{\sup}\left\{u^{⊤}v-f\left(u\right)\right\}\text{.}$$

In this paper, we efficiently solve the predictive pattern mining problem by effectively combining the primal and the dual problems.

### Sparse learning and safe screening

A class of methods for obtaining sparse solutions by using a sparsity-inducing regularization term such as Equation 3 is called sparse learning. In sparse learning, the set of features whose optimal solution is non-zero is called the active set, and is denoted by

$$A^{*}:=\left\{j\in \left[d\right]|\beta_{j}^{*}\neq 0\right\}\text{.}$$

One characteristic of sparse learning is that the optimal solution for a dataset containing the features in the active set gives the same optimal solution obtained for a dataset containing all features. Concretely, let us consider a superset $A⊇A^{*}$ of the active set and a dataset $\left(X_{A\cup \left\{0\right\}},y\right)$ containing only the features belonging to A. Then, the optimal solution for this dataset

$$\beta^{*}\left(A\right):=\underset{\beta \in {\mathbb{R}}^{\left|A\right|+1}}{\mathrm{argmin}}\;L_{y}\left(X_{A\cup \left\{0\right\}}\beta \right)+\Omega_{\left(\lambda ,\kappa \right)}\left(\beta \right)$$

has a property that

$$\beta_{j}^{*}\left(A\right)=\beta_{j}^{*},\forall j\;\in A\cup \left\{0\right\}\text{.}$$

This property implies that, if we can obtain a superset that contains the active set, it is sufficient to solve the optimization problem for a smaller dataset with smaller number of features.

In general, an active set cannot be obtained until the optimization problem is solved. However, by using an approach called safe screening, there is a case where it is possible to identify features that cannot be active in the optimal solution, i.e., features for which $\beta_{j}^{*}=0\text{,}j\in \left[d\right]$, before solving the optimization problem.

Specifically, by using theorems 23.5 and 31.3 of,^35^ it can be shown that the following relationship exists between the optimal solutions of the primal problem Equation 2 and the dual problem Equation 4, expressed as

(Equation 5) $$X\beta^{*}\in \partial L_{y}^{*}\left(-\alpha^{*}\right)\Leftrightarrow -\alpha^{*}\in \partial L_{y}\left(X\beta^{*}\right)\text{,}$$

(Equation 6) $$X^{⊤}\alpha^{*}\in \partial \Omega_{\left(\lambda ,\kappa \right)}\left(\beta^{*}\right)\Leftrightarrow \beta^{*}\in \partial \Omega_{\left(\lambda ,\kappa \right)}^{*}\left(X^{⊤}\alpha^{*}\right)\text{,}$$

where ∂f is the subgradient of f. In case of elastic net regularization, $\Omega^{*}$ is written as follows

$$\begin{array}{l} \Omega^{*}\left(X^{⊤}\alpha \right)=\sum_{j\in \left[d\right]\cup \left\{0\right\}}\omega_{j}^{*}\left(X_{:j}^{⊤}\alpha \right)\text{,}\\ \omega_{0}^{*}\left(v\right)=\left\{ \begin{array}{ll} 0 & \left(v=0\right)\\ +\infty & \left(v\neq 0\right) \end{array}\right.\text{,}\\ \omega_{j}^{*}\left(v\right)=\left\{ \begin{array}{ll} 0 & \left(\left|v\right|\leq \lambda \right)\\ {\left(\left|v\right|-\lambda \right)}^{2}/\left(2\kappa \lambda \right) & \left(\kappa \;>0\;\text{and}\;\left|v\right|>\lambda \right)\\ +\infty & \left(\kappa =0\;\text{and}\;\left|v\right|>\lambda \right) \end{array}\right.,\forall j\in \left[d\right]\text{,} \end{array}$$

and noting that subgradients are defined only on their feasible regions, i.e., v∈R such that $\omega_{j}^{*}\left(v\right)\neq +\infty$, $\partial \omega_{j}^{*}$ is also written down as

$$\partial \omega_{0}^{*}\left(0\right)=R\text{,}$$

(Equation 7) $$\partial \omega_{j}^{*}\left(v\right)=\left\{ \begin{array}{ll} \left\{0\right\} & \left(\left|v\right|<\lambda \right)\\ \left\{\text{sign}\left(v\right)\left(\left|v\right|-\lambda \right)/\left(\kappa \lambda \right)\right\} & \left(\kappa \;>0\;\text{and}\;\left|v\right|\geq \lambda \right)\\ {}[0,+\infty ) & \left(\kappa =0\;\text{and}\;v=\lambda \right)\\ (-\infty ,0] & \left(\kappa =0\;\text{and}\;v=-\lambda \right) \end{array}\right.,\forall j\in \left[d\right]\text{.}$$

From the right-hand side of Equation 6 and Equation 7, we can obtain the following simple relationship:

(Equation 8) $$\left|{X_{:j}}^{⊤}\alpha^{*}\right|<\lambda \Rightarrow \beta_{j}^{*}=0\text{,}\forall j\in \left[d\right]\text{.}$$

The basic idea of safe screening is to compute an upper bound on $\left|{X_{:j}}^{⊤}\alpha^{*}\right|$ in Equation 8. If the upper bound is smaller than λ, the condition in Equation 8 is satisfied, meaning that the optimal solution of the corresponding primal problem is $\beta_{j}^{*}=0$ and this feature can be removed beforehand. Since safe screening was first proposed, serveral improvements have been made,^36^^,^^37^^,^^38^^,^^39^^,^^40^^,^^41^ and its range of applications has been expanded.^42^^,^^43^^,^^44^^,^^45^^,^^46^^,^^47^^,^^48^ Especially, it is known that GAP safe screening^38^^,^^39^ has good performance of screening, and there are several studies that have utilized GAP safe screening in recent years. Extensions to specialized regularizers have been proposed.^49^^,^^50^ There is a method alleviates the assumption that the dual problem is strongly convex.^51^ Furthermore, there are studies on GAP safe screening for optimization algorithm based on stochastic gradient descent.^52^^,^^53^

In this paper, we also employ GAP safe screening. The basic idea of GAP safe screening is to use an arbitrary primal feasible solution $\tilde{\beta }\in R^{d+1}$ and an arbitrary dual feasible solution $\tilde{\alpha }\in R^{n}$ to compute an upper bound on $\left|{X_{:j}}^{⊤}\alpha^{*}\right|$. The following lemma states that, given a pair of primal and dual feasible solutions $\left(\tilde{\beta },\tilde{\alpha }\right)$, it is possible to determine the range of the dual optimal solution $\alpha^{*}$.

Lemma 2 (GAP safe screening rule): Suppose that ∇L is Lipschitz continuous with constant γ>0. For any pair of feasible solution $\left(\tilde{\beta },\tilde{\alpha }\right)$ and j∈[d], let us define, what is called, safe screening score as follows:

(Equation 9) $$u_{j}\left(\tilde{\beta },\tilde{\alpha }\right):=\left|{X_{:j}}^{⊤}\tilde{\alpha }\right|+r\left(\tilde{\beta },\tilde{\alpha }\right){\left\|X_{:j}-\Pi_{1}\left(X_{:j}\right)\right\|}_{2}\text{.}$$

Then,

(Equation 10) $$u_{j}\left(\tilde{\beta },\tilde{\alpha }\right)<\lambda \Rightarrow \beta_{j}^{*}=0\text{,}$$

where

$$\begin{array}{l} r\left(\tilde{\beta },\tilde{\alpha }\right):=\sqrt{2\gamma \left(P\left(\tilde{\beta }\right)-D\left(\tilde{\alpha }\right)\right)}\text{,}\\ \Pi_{u}\left(v\right):=\frac{u^{⊤}v}{u^{⊤}u}u,u,v\in R^{n}\text{.} \end{array}$$

($\Pi_{u}\left(v\right)$ is known as the projection of v onto u.)

The proof of lemma 2 is presented in Note S1. This lemma indicates that, given a pair of primal and dual feasible solutions $\left(\tilde{\beta },\tilde{\alpha }\right)$, we can first compute the upper bound $u_{j}\left(\tilde{\beta },\tilde{\alpha }\right)$ for each j∈[d], and remove the feature if $u_{j}\left(\tilde{\beta },\tilde{\alpha }\right)<\lambda$.

Note that this lemma requires both the primal feasible solution $\tilde{\beta }$ and the dual feasible solution $\tilde{\alpha }$; however, most algorithms that solve problem Equation 2 update only $\tilde{\beta }$ and hence $\tilde{\alpha }$ is not available. To obtain $\tilde{\alpha }$, we assume that the relationship on the right-hand side of Equation 5 holds even if $\tilde{\beta }$ is not optimal

$$-\bar{\alpha }=\nabla L\left(X\tilde{\beta }\right)\text{,}$$

where we assume that L is differentiable in this paper, and hence ∂L is equivalent to ∇L. Since there is no guarantee that $\bar{\alpha }$ calculated in this way is feasible, a process to move it into the feasible region is required (referred to as dual scaling). The detailed computation depends on L and Ω. For instance, if L is the squared loss, i.e., $L\left(v\right)=\|v-y{\|}_{2}^{2}/\left(2n\right)$ and κ>0 for elastic net, the feasible region is determined solely by the feasiblity condition of $\omega_{0}^{*}$, i.e., $1^{⊤}\alpha =0$. Thus, it is enough to compute $\tilde{\alpha }$ as $\tilde{\alpha }=\bar{\alpha }-{\bar{\alpha }}^{⊤}1$. If κ=0, there is a feasible condition on $\omega_{j}^{*}$, i.e., $\left|X_{:j}^{⊤}\alpha \right|\leq \lambda$ for all j∈[d] or $\max_{j\in \left[d\right]}\left|X_{:j}^{⊤}\alpha \right|$. Taking feasiblity of $\omega_{0}^{*}$ into account, we need to compute $\tilde{\alpha }$ such that $\tilde{\alpha }=\mu \left(\bar{\alpha }-{\bar{\alpha }}^{⊤}1\right)$, where

$$\mu =\min\left\{\max\left\{\frac{{\bar{\bar{\alpha }}}^{⊤}y}{\|\bar{\bar{\alpha }}{\|}_{2}^{2}},-\frac{\lambda }{\max_{j\in \left[d\right]}\left|X_{:j}^{⊤}\bar{\bar{\alpha }}\right|}\right\},\frac{\lambda }{\max_{j\in \left[d\right]}\left|X_{:j}^{⊤}\bar{\bar{\alpha }}\right|}\right\},\bar{\bar{\alpha }}=\bar{\alpha }-{\bar{\alpha }}^{⊤}1\text{,}$$

which is calculated to minimize duality gap P(β)−D(α) within the feasible region for faster convergence. Another example considered in this paper is the squared hinge loss, i.e., $L\left(v\right)=\sum_{i=1}^{n}\;\max\;{\left\{1-y_{i}v_{i},0\right\}}^{2}/\left(2n\right)$. In this case, there is a feasiblity condition on $L_{y}^{*}$ such that $y_{i}\alpha_{i}<0$. To satisfy the feasiblity conditions of both $L_{y}^{*}$ and $\Omega^{*}$, we apply different scaling for positive instances and negative instances, i.e., ${\tilde{\alpha }}_{i}=\mu_{+}{\bar{\alpha }}_{i}$ for $y_{i}=+1$ and ${\tilde{\alpha }}_{i}=\mu_{-}{\bar{\alpha }}_{i}$ for $y_{i}=-1$. We compute the ratio between the scaling factors as $\mu_{+}/\mu_{-}=-\sum_{i:y_{i}=-1}{\bar{\alpha }}_{i}/\sum_{i:y_{i}=+1}{\bar{\alpha }}_{i}$. Then we compute $\mu_{-}$ in the manner similar to the squared loss case,

$$\mu_{-}=\min\left\{\max\left\{\frac{{\bar{\bar{\alpha }}}^{⊤}y}{\|\bar{\bar{\alpha }}{\|}_{2}^{2}},0\right\},\frac{\lambda }{\max_{j\in \left[d\right]}\left|X_{:j}^{⊤}\bar{\bar{\alpha }}\right|}\right\},\bar{\bar{\alpha }}=\left\{ \begin{array}{cc} \mu_{+}/\mu_{-}{\bar{\alpha }}_{i} & \left(y_{i}=+1\right)\\ {\bar{\alpha }}_{i} & \left(y_{i}=-1\right) \end{array}\right.,\tilde{\alpha }=\mu_{-}\bar{\bar{\alpha }}\text{.}$$

If the ratio $\mu_{+}/\mu_{-}$ is unavailable due to $\sum_{i:y_{i}=+1}{\bar{\alpha }}_{i}=0$, then we compute the ratio $\mu_{-}/\mu_{+}$ and determine $\mu_{+}$ instead.

Our basic idea is to apply this GAP safe screening to predictive pattern mining. However, since the number of all possible features d is exponentially increasing, it is impossible to compute the upper bound $u_{j}\left(\tilde{\beta },\tilde{\alpha }\right)$ for each pattern. To address this challenge, in the next section, we extend the safe screening rule so that it can identify a group of removable patterns at once.

### SPP

The basic idea of SPP is to represent the relationship among patterns in a tree (Figure 2) and identify a group of patterns for which the optimal coefficients satisfy $\beta_{j}^{*}=0$ by pruning the tree. To obtain the pruning rule, we exploit the monotonicity of patterns, i.e., the occurrence of patterns decreases monotonically as pattern grows in the tree. We describe this property in the following lemma more specifically.

Lemma 3 (monotonicity of patterns): Let $p_{j},p_{k}\;\in S$ be patterns in ${\left\{S_{i}\right\}}_{i\in \left[n\right]}$ such that $p_{k}⊏p_{j}$.

Then, for any i∈[n],

$$x_{ik}=1\;\Rightarrow x_{ij}=1\text{.}$$

This is obvious because, if an input instance $S_{i}$ has $p_{k}$ (i.e., $p_{k}⊏S_{i}$), then $p_{k}⊏p_{j}⊏S_{i}$ also holds. Using this lemma, we derive the following theorem.

Theorem 4 (SPP rule): For any pair of feasible solution $\left(\tilde{\beta },\tilde{\alpha }\right)$ and j∈[d], let us define, what we call the SPP score, as follows:

(Equation 11) $$v_{j}\left(\tilde{\beta },\tilde{\alpha }\right):=\max\left\{\sum_{i:{\tilde{\alpha }}_{i}>0}x_{ij}{\tilde{\alpha }}_{i},-\sum_{i:{\tilde{\alpha }}_{i}<0}x_{ij}{\tilde{\alpha }}_{i}\right\}+r\left(\tilde{\beta },\tilde{\alpha }\right){\left\|X_{:j}\right\|}_{2}\text{.}$$

Then,

$$v_{j}\left(\tilde{\beta },\tilde{\alpha }\right)<\lambda \Rightarrow \beta_{k}^{*}=0\;\forall k\in \left[d\right]\;s.t.\;p_{k}⊏p_{j}\text{.}$$

The proof is presented in Note S2. Using theorem 4, it is possible to screen a group of patterns at once during the process of searching in the tree that represents the relationships between patterns. Specifically, when searching for the screen-able patterns in the tree from the root node to descent nodes, if the SPP score of a pattern $p_{j}$ corresponding with a certain node of the tree satisfies $v_{j}\left(\tilde{\beta },\tilde{\alpha }\right)<\lambda$, all patterns $p_{k}$ corresponding with its descendant nodes satisfies $p_{k}⊏p_{j}$, so they can be screened out as unnecessary patterns. Algorithm 1 in Note S4 shows the pseudo-code of the SPP.

To apply the SPP to actual predictive pattern mining, a pair of feasible solutions $\left(\tilde{\beta },\tilde{\alpha }\right)$ for the primal and dual problems is necessary. Although the SPP rules hold for any feasible solutions $\left(\tilde{\beta },\tilde{\alpha }\right)$, the tightness of the bound depends on the choice of feasible solutions. Specifically, because the tightness of the SPP bound is determined by the duality gap $P\left(\tilde{\beta }\right)-D\left(\tilde{\alpha }\right)$ of the feasible solution, the closer the pair of feasible solutions $\left(\tilde{\beta },\tilde{\alpha }\right)$ is to the (unknown) pair of optimal solutions $\left(\beta^{*},\alpha^{*}\right)$, the tighter the SPP bound will be. Therefore, when applying the SPP to actual predictive pattern mining, it is important to obtain feasible solutions that are sufficiently close to the optimal solutions.

### SPP for model selection

As mentioned in the previous section, to screen out features using the SPP, a feasible pair of solutions $\left(\tilde{\beta },\tilde{\alpha }\right)$ for the primal and dual problems, respectively, that are sufficiently close to the optimal solutions $\left(\beta^{*},\alpha^{*}\right)$ is necessary. In practical data analysis, it is often necessary to learn multiple models rather than just obtaining a single predictive pattern mining model, e.g., in selecting hyperparameters λ,κ, or evaluating the generalization performance through cross-validation (CV). In this section, we discuss how to apply the SPP for a series of model fittings in model selection. Our main idea is to use the optimal solutions of models fitted in slightly different problem settings (e.g., with similar hyperparameter values or with only a part of the data being different in CV) as reference feasible solutions for the SPP. Furthermore, we propose an extension of the SPP that enables more effective utilization of multiple reference feasible solutions in practical model fitting scenarios where multiple reference feasible solutions are naturally available.

First, in “SPP with multiple pairs of feasible solutions,” we describe an extension of safe screening using two different reference feasible solutions. Next, in “Multiple dynamic screening with SPP,” we introduce an approach called dynamic screening in which the solutions obtained during learning process are used as reference feasible solutions for the SPP. Furthermore, in “SPP with multiple hyperparameter selection,” we consider how to apply the SPP in model selection process in which two hyperparameters λ and κ are optimized. Finally, in “SPP with hyperparameter selection by CV,” we discuss how to apply the SPP when selecting hyperparameters using CV.

#### SPP with multiple pairs of feasible solutions

Let us consider the case where two feasible solutions $R_{1}:=\left({\tilde{\beta }}^{\left(1\right)},{\tilde{\alpha }}^{\left(1\right)}\right)$, $R_{2}:=\left({\tilde{\beta }}^{\left(2\right)},{\tilde{\alpha }}^{\left(2\right)}\right)$ are available. From lemma 3, the optimal solution $\alpha^{*}$ must be contained in both of the two hyperspheres $B_{1}:=B\left(R_{1}\right)$ and $B_{2}:=B\left(R_{2}\right)$. Therefore, it is possible to further narrow down the range of $\alpha^{*}$ to $B_{1}\cap B_{2}$, and consider a tighter upper bound

$$\max_{\alpha \in B_{1}\cap B_{2}\cap H}\left|{X_{:j}}^{⊤}\alpha \right|\leq \min\left\{\max_{\alpha \in B_{1}\cap H}\left|{X_{:j}}^{⊤}\alpha \right|,\max_{\alpha \in B_{2}\cap H}\left|{X_{:j}}^{⊤}\alpha \right|\right\}\text{,}$$

where $H=\left\{\alpha \in R^{n}|1^{⊤}\alpha =0\right\}$ is introduced by the feasiblity of $\omega_{0}^{*}$. By using this tighter upper bound in the safe screening, we expect that more inactive patterns can be screened out. For a pattern j, if $\max_{\alpha \in B_{1}\cap B_{2}\cap H}\left|{X_{:j}}^{⊤}\alpha \right|<\lambda \leq \min\left\{\max_{\alpha \in B_{1}\cap H}\left|{X_{:j}}^{⊤}\alpha \right|,\max_{\alpha \in B_{2}\cap H}\left|{X_{:j}}^{⊤}\alpha \right|\right\}$, then it is not possible to remove the pattern j using either $B_{1}$ or $B_{2}$ alone, but it becomes possible to remove it by using the intersection of $B_{1}$ and $B_{2}$. The following theorem indicates that $\max_{\alpha \in B_{1}\cap B_{2}\cap H}\left|{X_{:j}}^{⊤}\alpha \right|$ can be expressed in a closed form and can be computed in O(n) time.

Theorem 5 (multiple safe screening rule): For any pair of primal-dual feasible solutions $R_{1}=\left({\tilde{\beta }}^{\left(1\right)},{\tilde{\alpha }}^{\left(1\right)}\right),R_{2}=\left({\tilde{\beta }}^{\left(2\right)},{\tilde{\alpha }}^{\left(2\right)}\right)$, and for any j∈[d], it holds that

$$u_{j}^{\prime }\left(R_{1},R_{2}\right):=\max_{\alpha \in B_{1}\cap B_{2}\cap H}\left|{X_{:j}}^{⊤}\alpha \right|=\max\left\{u_{j}^{+},u_{j}^{-}\right\}<\lambda \Rightarrow \beta_{j}^{*}=0\text{,}$$

where

$$u_{j}^{+}:=\left\{ \begin{array}{cc} {X_{:j}}^{⊤}{\tilde{\alpha }}^{\left(1\right)}+r\left(R_{1}\right){\left\|X_{:j}-\Pi_{1}\left(X_{:j}\right)\right\|}_{2}, & X_{:j}\in C_{1},\\ {X_{:j}}^{⊤}{\tilde{\alpha }}^{\left(2\right)}+r\left(R_{2}\right){\left\|X_{:j}-\Pi_{1}\left(X_{:j}\right)\right\|}_{2}, & X_{:j}\in C_{2},\\ {X_{:j}}^{⊤}{\tilde{\alpha }}^{\prime }+r^{\prime }{\left\|X_{:j}-\Pi_{1}\left(X_{:j}\right)-\Pi_{\delta }\left(X_{:j}\right)\right\|}_{2}, & \text{otherwise}, \end{array}\right.$$

$$u_{j}^{-}:=\left\{ \begin{array}{cc} -{X_{:j}}^{⊤}{\tilde{\alpha }}^{\left(1\right)}+r\left(R_{1}\right){\left\|X_{:j}-\Pi_{1}\left(X_{:j}\right)\right\|}_{2}, & -X_{:j}\in C_{1},\\ -{X_{:j}}^{⊤}{\tilde{\alpha }}^{\left(2\right)}+r\left(R_{2}\right){\left\|X_{:j}-\Pi_{1}\left(X_{:j}\right)\right\|}_{2}, & -X_{:j}\in C_{2},\\ -{X_{:j}}^{⊤}{\tilde{\alpha }}^{\prime }+r^{\prime }{\left\|X_{:j}-\Pi_{1}\left(X_{:j}\right)-\Pi_{\delta }\left(X_{:j}\right)\right\|}_{2}, & \text{otherwise}, \end{array}\right.$$

and

$$\begin{array}{ll} \delta & :={\tilde{\alpha }}^{\left(1\right)}-{\tilde{\alpha }}^{\left(2\right)}\text{,}\\ {\tilde{\alpha }}^{\prime } & :=t{\tilde{\alpha }}^{\left(1\right)}+\left(1-t\right){\tilde{\alpha }}^{\left(2\right)}\text{,}\\ r^{\prime } & :=\sqrt{r{\left(R_{2}\right)}^{2}-t^{2}\|\delta {\|}_{2}^{2}}\text{,}\\ t & :=\frac{1}{2}\left(1+\frac{r{\left(R_{2}\right)}^{2}-r{\left(R_{1}\right)}^{2}}{\|\delta {\|}_{2}^{2}}\right)\text{,}\\ C_{1} & :=\left\{a\in R^{n}|\frac{a^{⊤}\delta }{{\left\|a-\Pi_{1}\left(a\right)\right\|}_{2}}\leq \frac{r{\left(R_{2}\right)}^{2}-r{\left(R_{1}\right)}^{2}-\|\delta {\|}_{2}^{2}}{2r\left(R_{1}\right)}\right\}\text{,}\\ C_{2} & :=\left\{a\in R^{n}|\frac{a^{⊤}\delta }{{\left\|a-\Pi_{1}\left(a\right)\right\|}_{2}}\geq \frac{r{\left(R_{2}\right)}^{2}-r{\left(R_{1}\right)}^{2}+\|\delta {\|}_{2}^{2}}{2r\left(R_{2}\right)}\right\}\text{.} \end{array}$$

The proof of this theorem is presented in Note S3.

Instead of the safe screening rule in theorem 5, we may consider the safe pruning rule for two reference solutions. However, unlike the safe screening case above, we conjecture that the pruning conditions cannot be written in a closed form. So, for the safe pruning with two reference solutions, we just apply two pruning rules derived by each of the solutions:

$$v_{j}^{\prime }\left(R_{1},R_{2}\right):=\min\left\{v_{j}\left(R_{1}\right),v_{j}\left(R_{2}\right)\right\}<\lambda \Rightarrow \forall p_{j}⊏p_{k},\beta_{k}^{*}=0\text{.}$$

In addition, if we have three or more reference solutions, we expect that we can screen out more features. However, we conjecture that the screening conditions become very complicated as the number of reference solutions increases. □

#### Multiple dynamic screening with SPP

In this section, we describe the extension of multiple safe screening to dynamic screening.^41^ Dynamic screening is a method of performing safe screening using feasible solutions obtained during optimization. Because the performance of safe screening depends on how close the feasible solution is to the optimal solution, more patterns tend to be removed with updated solutions. This means that, if the update to the solution is not substantial enough, the performance of safe screening may not differ significantly between before and after the updates. In the case where multiple feasible solutions are available, all of the solutions must be sufficiently updated. However, updating multiple solutions needs additional computational costs, so it is necessary to consider the trade-off between the cost of updating multiple solutions and the number of patterns that can be removed by safe screening.

To reduce the cost of updating multiple solutions, we restrict the number of multiple solutions updates to M∈N. Specifically, we repeat updates and screening for multiple solutions up to M iterations, and then keep the single solution that has the smallest duality gap, which is an indicator of proximity to the optimal solution, while the others are discarded. Although there are no theoretical indicators to select M, we demonstrate that the optimizations with M=1 show better results, and setting M larger than 1 does not have much of an effect in numerical experiments.

#### SPP with multiple hyperparameter selection

**Figure 4:**
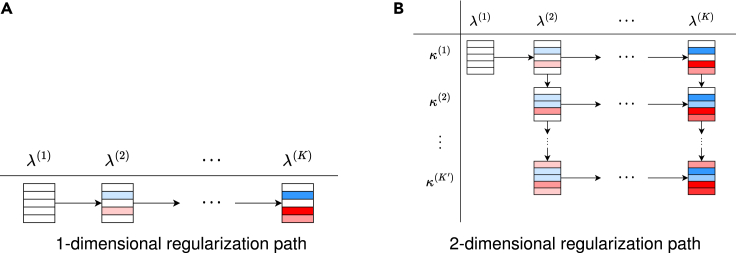
Regularization path for the one-dimensional and two-dimensional regularization parameter Schematic illustration of (A) regularization path for the $L_{1}$-norm regularization parameter λ and (B) two-dimensional regularization path for the L1-norm regularization parameter λ and the relative regularization parameter κ for the L2-norm in the elastic net. The rectangles in each cell represent the $\beta_{j}^{*}$ at the corresponding regularization parameter. The color of each rectangle indicates the value of $\beta_{j}^{*}$ where red/blue shows its signs while the thickness of the color indicates the absolute value (illustrating the increase of active (non-zero) coefficients and their absolute values as λ decreases). Note that, in (B), when $\lambda =\lambda^{\left(1\right)}$ and $\beta^{*}=0$, it is not necessary to change κ.

In this section, we describe a method for accelerating the computation of regularization paths for multiple hyperparameters using screening and pruning with multiple reference feasible solutions. Specifically, we consider the regularization paths for the two hyperparameters of the elastic net. When there are two hyperparameters, we can consider a two-dimensional regularization path as shown in Figure 4, where the sequence of regularization parameters for the $L_{1}$ norm is represented by $\lambda^{\left(1\right)},\lambda^{\left(2\right)},\ldots$, and the sequence of regularization parameters that adjust the relative strength of the $L_{2}$ norm is represented by $\kappa^{\left(1\right)},\kappa^{\left(2\right)},\ldots$. For a given set of hyperparameters $\left(\lambda^{\left(t\right)},\kappa^{\left(t^{\prime }\right)}\right)$, there are two feasible solutions that can be used as reference solutions for optimization, i.e., the optimal solution at $\left(\lambda^{\left(t-1\right)},\kappa^{\left(t^{\prime }\right)}\right)$ and the optimal solution at $\left(\lambda^{\left(t\right)},\kappa^{\left(t^{\prime }-1\right)}\right)$.

For the hyperparameters λ and κ, we considered the following sequences of candidates: $\lambda^{\left(k\right)},k\in \left[K\right]$ such that $\lambda^{\left(1\right)}>\lambda^{\left(2\right)}>\cdots >\lambda^{\left(K\right)}$ and $\kappa^{\left(k^{\prime }\right)},k^{\prime }\in K^{\prime }$ such that $\kappa^{\left(1\right)}<\kappa^{\left(2\right)}<\cdots <\kappa^{\left(K^{\prime }\right)}$, respectively. In many cases, as κ increases for a fixed $\lambda^{\left(k\right)}$, the number of patterns for which $\beta_{j}^{*}\neq 0$ decreases. Therefore, by setting $\kappa^{\left(k^{\prime }\right)}$ in this way, the number of patterns for which $\beta_{j}^{*}\neq 0$ increases as $k^{\prime }$ becomes larger. Furthermore, we set $\lambda^{\left(1\right)}$ as the smallest λ value such that $\beta^{*}=0$ and set $\kappa^{\left(1\right)}=0$.

In the case of having two regularization parameters, iterative optimization is performed in a manner analogous to the case of a single parameter. There are multiple possible options to optimize the regularization parameter sequence. In this paper, we adopt an option to optimize them in the order of $\left(\lambda^{\left(1\right)},\kappa^{\left(1\right)}\right)$, $\left(\lambda^{\left(1\right)},\kappa^{\left(2\right)}\right)$, …, $\left(\lambda^{\left(1\right)},\kappa^{\left(K^{\prime }\right)}\right)$, $\left(\lambda^{\left(2\right)},\kappa^{\left(1\right)}\right)$, …, $\left(\lambda^{\left(2\right)},\kappa^{\left(K^{\prime }\right)}\right)$, …, $\left(\lambda^{\left(K\right)},\kappa^{\left(K^{\prime }\right)}\right)$.

Let R denote the set of feasible solutions. During optimization at $\left(\lambda^{\left(k\right)},\kappa^{\left(k^{\prime }\right)}\right)$, if k>1, the optimal solution at $\left(\lambda^{\left(k-1\right)},\kappa^{\left(k^{\prime }\right)}\right)$ is appended to R. On the other hand, if $k^{\prime }>1$, the optimal solution at $\left(\lambda^{\left(k\right)},\kappa^{\left(k^{\prime }-1\right)}\right)$ is appended to R. When |R|=1, we execute safe pruning and screening using a single feasible solution in conventional way. In contrast, when |R|=2, we execute pruning and screening using two feasible solutions. The detailed algorithm is described in Algorithms 2 and 3 in Note S4. The former is for one-dimensional settings and the latter is for two-dimensional ones. In exploiting theorem 5 in the algorithm, we require two pairs of feasible solutions. It is obvious that the optimal solution at $\left(\lambda^{\left(k-1\right)},\kappa^{\left(k^{\prime }\right)}\right)$ and $\left(\lambda^{\left(k\right)},\kappa^{\left(k^{\prime }-1\right)}\right)$ may not be feasible for optimization at $\left(\lambda^{\left(k\right)},\kappa^{\left(k\right)}\right)$. To address this issue, we utilize dual scaling as described in “Sparse learning and safe screening” for both solutions. Note that this approach can be easily extended to cases with three or more hyperparameters although it is not explicitly described in this paper.

#### SPP with hyperparameter selection by CV

CV is commonly used for determining hyperparameters. In CV, the following steps are taken to determine hyperparameters. First, the given dataset is divided into several groups. Then, one of the groups is used as the validation set, while the remaining groups are used for model training. Performance metrics such as prediction errors and classification accuracy are calculated for each hyperparameter(s) candidate using the validation data. This process is repeated by sequentially swapping the validation group, and the metrics are averaged for each hyperparameter(s) candidate. The hyperparameter candidate(s) with the highest average score is selected as the best hyperparameter(s). In such a CV process, a sequence of optimization problems with slightly different training set are solved one by one for each hyperparameter.

**Figure 5:**
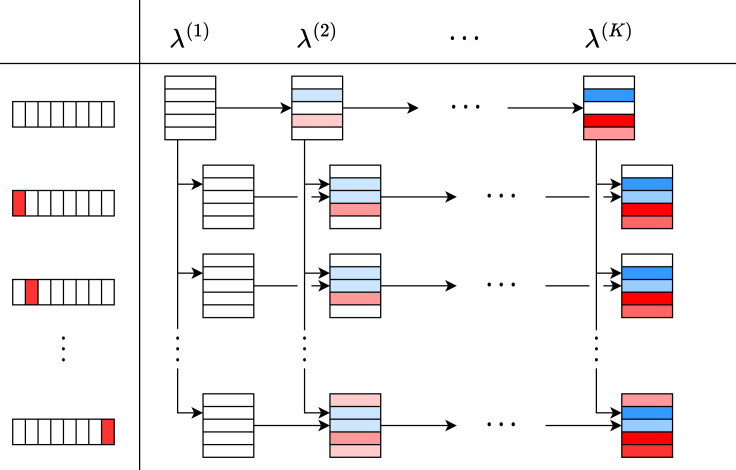
A schematic illustration of how to use feasible solutions in hyperparameter selection based on CV The left rectangle represents the training data, with white corresponding with the training data and red with the validation data. In using multiple solutions for CV setting, we not only use the optimal solution at the previous regularization parameter, but also use the optimal solution trained with the entire data.

Our idea here is to use optimal solutions obtained at different steps of CV as another reference feasible solutions. Specifically, we use two reference feasible solutions obtained as the optimal solutions at different hyperparameters and at different CV steps and perform safe screening and pruning using these two reference feasible solutions as described in “SPP with multiple pairs of feasible solutions.. Let $I^{\left(1\right)}=\left[n\right]$ denote the set of indices of the entire dataset, and consider a sequence of its subset, denoted by $I^{\left(2\right)},I^{\left(3\right)},\ldots \text{,}I^{\left(K\right)}⊊I^{\left(1\right)}$. Figure 5 shows a schematic diagram of this procedure, and the details are described in Algorithm 4 in Note S4. Given a sequence of subscript sets ${\left\{I^{\left(k\right)}\right\}}_{k\in \left[K\right]}$ and a sequence of hyperparameters ${\left\{\lambda^{k^{\prime }}\right\}}_{k^{\prime }\in \left[K^{\prime }\right]}$, we use the reference feasible solutions of the optimal solutions at $\left(I^{\left(1\right)},\lambda^{\left(k^{\prime }\right)}\right)$ and $\left(I^{\left(k\right)},\lambda^{\left(k^{\prime }-1\right)}\right)$ during optimization of $\left(I^{\left(k\right)},\lambda^{\left(k^{\prime }\right)}\right)$. Similar to the case of multiple hyperparameter selection, we apply dual scaling to both solutions to ensure their feasibility. Note that, in Algorithm 3, κ is fixed for simplicity, but it is possible to extend it to select both λ and κ.

### Numerical experiments

In this section, we describe numerical experiments that verify the effectiveness of the proposed SPP method and its extension in model selection scenario.

#### SPP with multiple hyperparameter selection

We first conducted a comparison of computation times for two-dimensional regularization path calculations for the two regularization parameters, λ and κ, in the elastic net. We compared the performances of Single-SPP, which utilizes only a single reference feasible solution, and multi-SPP, which uses multiple reference feasible solutions. In the case of multi-SPP, experiments were performed for M∈{0,1,2,4}, where M represents the number of times that multiple dynamic screening is executed (note that M=0 signifies the use of multiple solutions solely for screening and pruning at the start of optimization, with dynamic screening performed using only a single solution thereafter). Regarding λ, we investigated cases where the number of partitions from $\lambda_{\max}$ to $0.01\lambda_{\max}$ was 5, 10, 20, and 40, where $\lambda_{\max}$ is the smallest λ that makes all patterns inactive (see “Experimental setup”). As for κ sequence, we investigated the cases with κ∈{0,0.01,0.1,1.0,10.0,100.0}. For both single-SPP and multi-SPP, the optimization was performed based on Algorithm 3 in Note S4. In single-SPP, only the optimal solution from the previous λ was used as a reference feasible solution.

**Figure 6:**
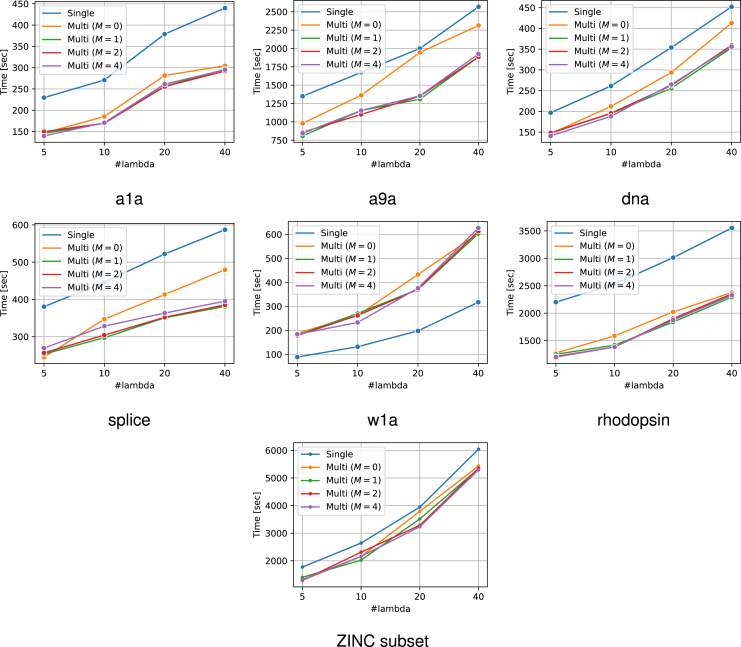
Computation time for the entire regularization path for each dataset The horizontal axis shows how many partitions of λ were made. It can be confirmed that the use of multiple solutions is effective in most cases. In addition, the multi-dynamic screening also often leads to a reduction in computation time.

**Figure 7:**
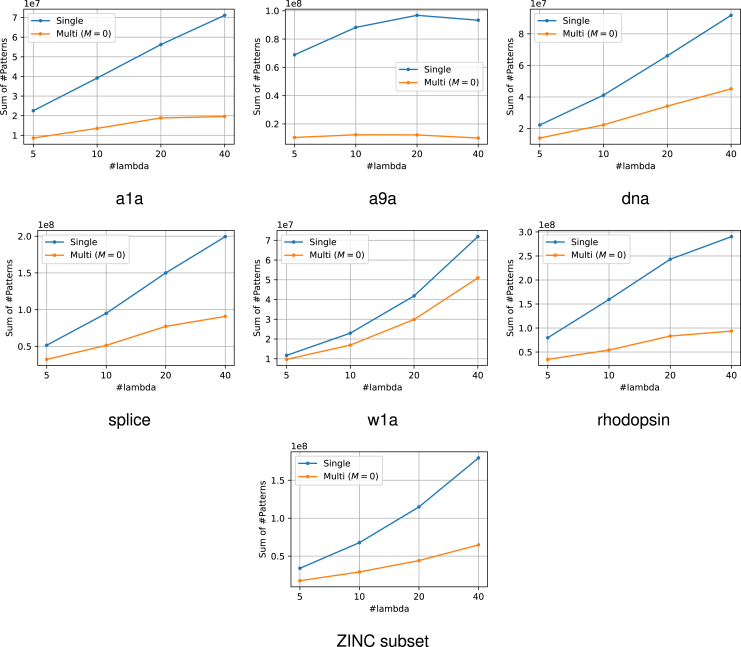
The number of patterns after SPP before optimization These values represent the sums over the entire regularization path for each number of λs. Our multiple feasible solution approach significantly reduces the number of patterns in most datasets. Note that the number of patterns after pruning is irrelevant to M, hence we compared only the single reference case and multiple reference case with M=0.

**Figure 8:**
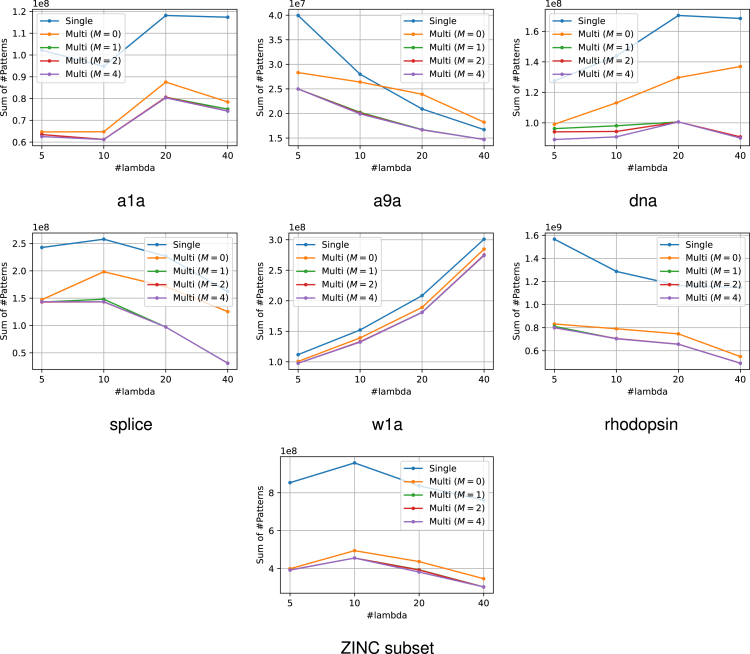
The number of patterns after safe screening during optimization These values represent the sums over the iterations until convergence across the entire regularization path. Note that for fair comparison, we exclude iterations from the sum where optimization has already converged with some M. Multiple reference screening reduces numerous patterns and multiple dynamic screening (i.e., M>0) reduces the number of patterns further in some cases.

The experimental results are presented in Figure 6. From this figure, it can be confirmed that the use of multiple reference solutions is effective in many cases. Moreover, in multi-SPP, dynamic screening can improve performance to some extent even for small values of M. The increase in M did not result in a considerable increase in computational time. The number of patterns remaining after SPP is also displayed in Figure 7. It is confirmed that the use of multiple feasible solutions leads to a significant reduction of the number of patterns. Note that the value of M is irrelevant to pruning, therefore we compared only the single feasible case and multiple feasible case with M=0. Furthermore, to demonstrate the significance of multiple dynamic screening, we show the number of patterns after safe screening during optimization in Figure 8. For fair comparison, we summed the number of patterns over the iterations until convergence, except for the iterations where optimization has already converged with some M. We observe that multiple dynamic screening reduces the number by more than one-half in some cases. Although no improvement in computation speed was observed for w1a, we confirmed that the number of patterns is reduced by our method. Therefore, this may be due to the fact that the additional cost of computing multiple solutions outweighs the benefits of reducing the computation cost with multiple solutions.

#### SPP with hyperparameter selection by CV

Next, we conducted experiments to investigate the use of reference feasible solutions in the hyperparameter selection process based on CV. To compare the computational costs, we investigated the case where one of the two hyperparameters, κ, was fixed at 0, and only λ was varied as in “SPP with multiple hyperparameter selection.” In terms of CV configuration, we compared the computation time of leave one out CV. Specifically, we constructed 10 leave-one-out datasets at random, and compared the relative computational costs of each method option and problem setting. For a single-SPP with one reference feasible solution, we used the optimal solution from the previous λ, as in “SPP with multiple hyperparameter selection.”

**Figure 9:**
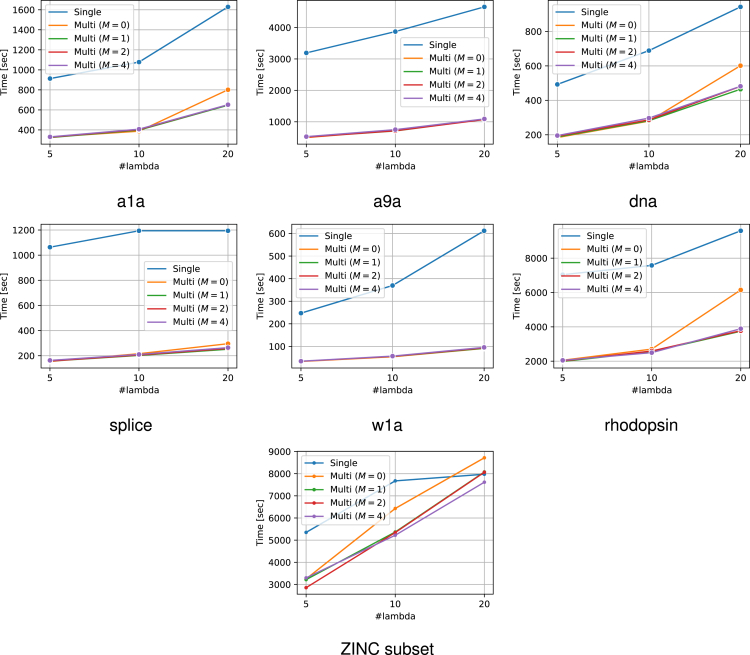
The computation time for leave-one-out CV for each dataset The use of multiple solutions is effective for all the cases. The multi-dynamic screening is effective in settings where the number of λ is large.

**Figure 10:**
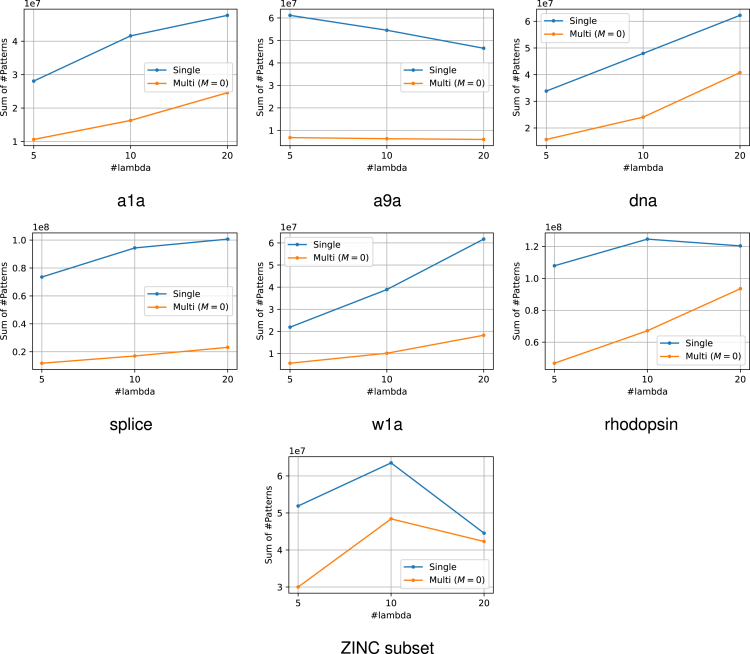
The number of patterns remaining after SPP, before optimization for leave-one-out CV Similar to the case of multiple hyperparameter selection, our method can eliminate numerous patterns.

**Figure 11:**
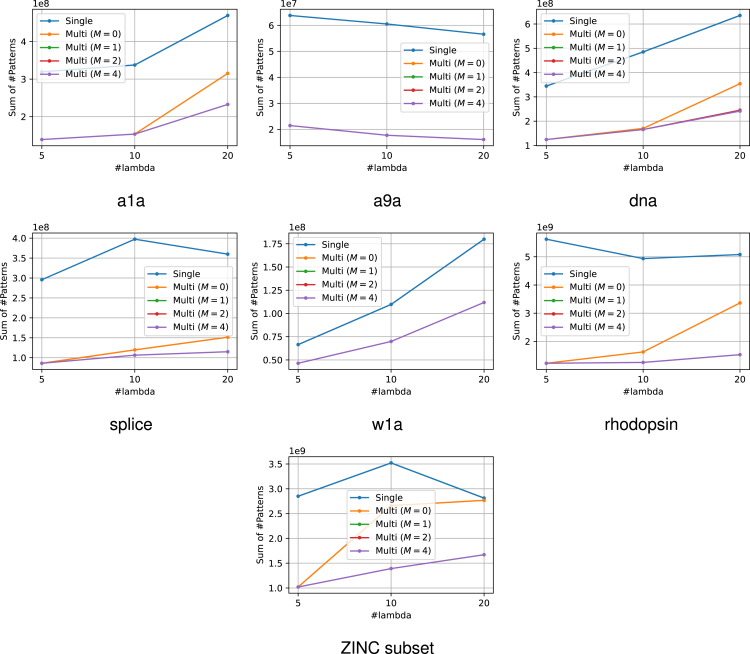
The number of patterns remaining after safe screening during optimization for leave-one-out CV Similar to Figure 8, multiple feasible screening eliminates numerous patterns, while multiple dynamic screening reduces the number of patterns further, particularly in cases where the number of λ s is smaller in some datasets.

Figure 9 shows the experimental results. In multi-SPP, a significant reduction in overall computation time can be achieved by using the optimal solution with the entire dataset as a reference feasible solution. The number of patterns after SPP is displayed in Figure 10 and safe screening in Figure 11. From these figures, we can confirm a reduction in the number of patterns in most cases. While there were some cases where dynamic screening in multi-SPP showed some effectiveness, no significant changes in performance were observed in many other cases. We conjecture that this is due to a trade-off between the reduction in computation time resulting from the effectiveness of screening with increasing M and the increase in computation time necessary for updating multiple optimal solutions.

#### Comparison with boosting-based methods

Finally, we compared the computational costs of the proposed SPP and the existing boosting-based approach in predictive pattern mining.^1^^,^^7^^,^^8^ The boosting-based approach involves sequential addition of patterns to the prediction model, necessitating tree traverse search at each step. In contrast, the SPP requires only a single tree traverse search (under fixed regularization parameter), thereby exhibiting a computational advantage.

To conduct fair comparisons, we set the problem such that both methods start from $\lambda_{\max}$ and seek the optimal solution at $0.01\lambda_{\max}$. For the boosting-based method, we measured the computational cost taken from the optimal solution at $\lambda_{\max}$, which does not include any patterns, to adding one pattern at a time until arriving at the optimal solution at $0.01\lambda_{\max}$. For the SPP, we considered the one-dimensional regularization path from $\lambda_{\max}$ to $0.01\lambda_{\max}$ and measured the computational cost when performing the same process as in “SPP with multiple hyperparameter selection.” Both methods used the coordinate gradient descent method^54^ for optimization, and the another hyperparameter κ was set to 0.

**Figure 12:**
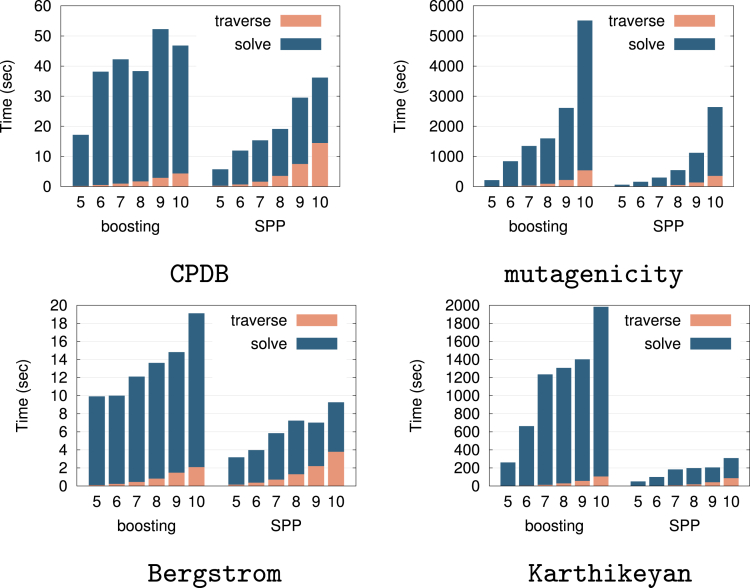
Computational time comparison for graph classification and regression The horizontal axis represents the maximum length of patterns that are mined. Each bar contains computational time taken in the tree traverse (traverse) and the optimization procedure (solve) respectively. Same figure as our preliminary work.^33^

First, we considered the problems of graph classification and graph regression with chemical compound datasets as examples in predictive graph mining. Specifically, we used two datasets for graph classification: CPDB (“Helma CPDB Mutagenicity Subset,” n=684) and Mutagenicity (“Bursi Mutagenicity Dataset,” n=4337), and two datasets for graph regression: Bergstrom (“Bergstrom Melting Point Dataset,” n=185) and Karthikeyan (“Karthikeyan Melting Point Dataset,” n=4450). All datasets were retrieved from http://cheminformatics.org/datasets/. Note that, since these datasets were downloaded when our preliminary work^33^ was conducted, and the websites above were closed later, we also present the link to the archived website: http://web.archive.org/web/20150503130239/http://cheminformatics.org/datasets/. In addition, since the number of instances n were mistakenly noted in the preliminary work, we noted collect numbers. Figure 12 shows the computational cost of the boosting-based method (boosting) and the SPP. In all the cases, SPP is faster than boosting, and the differences become more significant as the maximum length of patterns increases. The results also indicate that the tree traverse time are not so different between the two methods. We conjecture that this is because the most time-consuming part of gSpan is the isomorphism check, which is required to avoid enumerating duplicated graphs.

**Figure 13:**
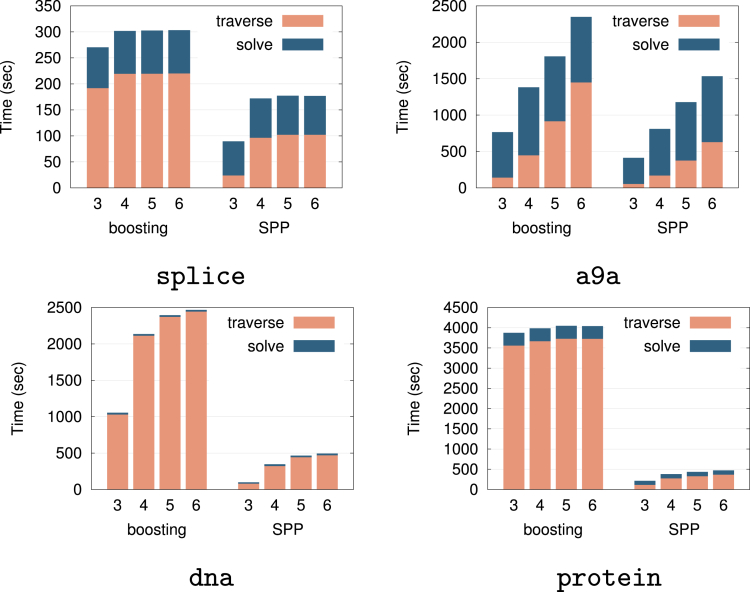
Computational time comparison for item set classification and regression The horizontal axis represents the maximum length of patterns that are mined. Each bar contains computational time taken in the tree traverse (traverse) and the optimization procedure (solve) respectively. Same figure as our preliminary work.^33^

Next, we considered classification and regression in item set predictive mining. We used two datasets for classification: splice (n=1000) and a9a (n=32561), and two datasets for regression: dna (n=2000) and protein (n=6621). The results are shown in Figure 13. In all the cases, SPP was faster than boosting. Unlike graph mining cases, the tree traverse time of the SPP was smaller than that of boosting-based method. We conjecture that this is because boosting-based methods require multiple enumeration of patterns, while the SPP requires only one enumeration.

## Discussion

Structured data such as sets, graphs, and sequences are common in many fields, and it is necessary to develop machine learning methods to handle such structure data. Although neural networks for structured data have seen significant recent development and can achieve good predictive performances, practical problems often require interpretation and explanation of the model behavior and extracting important features. In this study, we propose a pattern mining approach for machine learning modeling of structured data that can both prediction ability and explainability.

The challenge of extracting features from structural data lies in the exponential increase in the number of potential unified inconsistent notation sub-structures that can serve as features. This difficulty has led to the development of various pattern mining algorithms, particularly in the task of enumeration. However, research that integrates pattern mining with predictive modeling, such as regression or classification, is limited. To our knowledge, only boosting-based approaches have been proposed, which are inefficient due to the requirement of a tree traverse for each additional feature.

In this study, we addressed a common problem in the field of pattern mining by introducing safe screening. Safe screening is a technique developed in the field of sparse modeling that allows for the identification of redundant features before solving the optimization problem, which can significantly reduce the computational cost. However, as noted in the “Introduction” and “SPP,” applying conventional safe screening to many patterns is not feasible. To address this, we proposed the SPP method, which efficiently handles multiple features in a single tree traverse, as opposed to a boosting-based approach. By leveraging the monotonicity of patterns, as described in lemma 3, the proposed SPP method can safely eliminate a subset of patterns with only a moderate increase in computational cost.

For a comparison with recent methods, there are several papers which utilize GAP safe screening as described in “Sparse learning and safe screening.” The extension to special regularizers such as non-convex penalty are proposed.^49^^,^^50^ Our concept of using multiple solutions can be incorporated into these methods and can accelerate their optimizations. Several studies exist in the paradigm of stochastic gradient method.^52^^,^^53^ Although it is not clear that our method can be directly applied to them, this topic is worth discussing in future work. Additionally, the sphere refinement technique^51^ can further accelerate SPP more in some loss functions. We would like to demonstrate its effectiveness in the context of predictive pattern mining in the future.

Finally, we demonstrated the effectiveness of the proposed SPP method in the entire model building process, including hyperparameter selection and CV in this paper. Especially, it has been observed that the SPP method works more efficiently if two reference feasible solutions are available, as experimentally demonstrated in “SPP with multiple hyperparameter selection” and “SPP with hyperparameter selection by CV.” Additionally, in “Multiple dynamic screening with SPP,” we discussed that the parameter M, which determines the number of dynamic safe screening applications with multiple solutions, needs to be appropriately chosen. The experiments in “SPP with multiple hyperparameter selection” and “SPP with hyperparameter selection by CV” have shown that not only small but also large values of M may increase computational costs. In our experiments, it is demonstrated that our method performed well at M=1; however, a more general selection criterion is a topic for future work.

## Experimental procedures

### Resource availability

#### Lead contact

Further information and requests for resources and reagents should be directed to and will be fulfilled by the lead contact, Ichiro Takeuchi (ichiro.takeuchi@mae.nagoya-u.ac.jp).

#### Materials availability

This study did not generate new unique reagents.

#### Data and code availability

The code for “SPP with multiple hyperparameter selection” and ”SPP with hyperparameter selection by CV” has been deposited at Zenodo under the DOI https://doi.org/10.5281/zenodo.10017314, and the code for “Comparison with boosting-based methods” (same as our preliminary work^33^) is at Zenodo under the DOI https://doi.org/10.5281/zenodo.10017836. They are publicly available as of the date of publication.

Any additional information required to reanalyze the data reported in this paper is available from the lead contact upon request.

### Experimental setup

First, we describe the settings common to all the experiments. We compared the computation time of the entire or partial regularization path with respect to the hyperparameters λ and/or κ. Regarding λ, we defined $\lambda_{\max}$ as the largest value of λ for which $\exists j\in \left[d\right],\beta_{j}^{*}\neq 0$, and constructed a sequence of λs by partitioning the interval from $\lambda_{\max}$ to $0.01\lambda_{\max}$ into equally spaced values on a logarithmic scale, where the number of partitions is varied depending on the experimental options. The datasets used in the experiments in “SPP with multiple hyperparameter selection” and “SPP with hyperparameter selection by CV” are presented in Table 1. We used the squared hinge loss function as L for classification problem, and the squared loss function for regression. The coordinate descent method was employed for optimization, with a convergence criterion $\epsilon =10^{-4}$. During optimization, dynamic screening was performed every other iteration for the first T=5 cycles, and subsequently executed once every ten iterations. PrefixSpan^55^ was employed as the mining algorithm for both set and sequence mining tasks, while gSpan^56^ was used for graph mining tasks.

**Table 1:** The list of datasets used in the experiments

Dataset	No.	Structure type	Maximum length of patterns	Problem
a1a	1,605	item set	5	classification
a9a	32,561	item set	5	classification
dna	2,000	item set	3	regression
splice	1,000	item set	3	classification
w1a	2,477	item set	3	classification
rhodopsin	1,162	sequence	50	regression
ZINC subset	500	graph	10	regression

Datasets used in the experiments in “Numerical experiments.” The dataset rhodopsin is studied in Inoue et al.^57^ and Karasuyama et al.^58^ The dataset ZINC subset is a random sample from ZINC database^59^ with molecular weights between 450 and 500, and the objective variable is “logP” (logarithm of partition coefficient). The remaining datasets are listed in Chang and Lin.^60^

## Supplemental information

Document S1. Notes S1–S4

Document S2. Article plus supplemental information
