# Supplementary material for: Efficient model selection for predictive pattern mining model by safe pattern pruning
Source: Patterns (N Y). 2023 Dec 1;4(12):100890. doi: 10.1016/j.patter.2023.100890 (PMC10724371; doi:10.1016/j.patter.2023.100890)
Supplement: Document S1. Notes S1–S4 [file mmc1.pdf]

Patterns, Volume *4*

Supplemental information

Efficient model selection for predictive pattern mining model by safe pattern pruning

Takumi Yoshida, Hiroyuki Hanada, Kazuya Nakagawa, Kouichi Taji, Koji Tsuda, and Ichiro Takeuchi

Supplemental note S1: Proof of Lemma 2

Proofs of the following lemmas are similar to previous studies[1,2], however, we explicitly state the proof for our setup.

Before proving Lemma 2, we prove two other lemmas: Lemmas 7 and 8.

Definition 6. *For a convex function $f : \mathbb{R}^n \to \mathbb{R}$ and a vector $\boldsymbol{v} \in \mathbb{R}^n$, $\boldsymbol{g} \in \mathbb{R}^n$ is called a* subderivative *of f at $\boldsymbol{v}$ if the following condition is met:*

$$\forall \boldsymbol{z} : \quad f(\boldsymbol{z}) - f(\boldsymbol{v}) \geq \boldsymbol{g}^\top (\boldsymbol{z} - \boldsymbol{v}).$$

We denote by $\partial f(\boldsymbol{v})$ the set of all subderivatives of f at $\boldsymbol{v}$, since such $\boldsymbol{g}$ may not be unique.

Lemma 7. *For any pair of feasible solutions $(\tilde{\boldsymbol{\beta}}, \tilde{\boldsymbol{\alpha}})$, the dual optimal solution $\boldsymbol{\alpha}^*$ is within the intersection of a $\mathbb{R}^n$-ball $B(\tilde{\boldsymbol{\beta}}, \tilde{\boldsymbol{\alpha}})$ and a hyperplane H such that*

$$\begin{aligned} B(\tilde{\boldsymbol{\beta}}, \tilde{\boldsymbol{\alpha}}) &:= \{\boldsymbol{\alpha} \in \mathbb{R}^n \mid \|\boldsymbol{\alpha} - \tilde{\boldsymbol{\alpha}}\|_2 \leq r(\tilde{\boldsymbol{\beta}}, \tilde{\boldsymbol{\alpha}})\}, \\ H &:= \{\boldsymbol{\alpha} \in \mathbb{R}^n \mid \boldsymbol{\alpha}^\top \mathbf{1} = 0\}, \end{aligned} \tag{12}$$

where $r(\tilde{\boldsymbol{\beta}}, \tilde{\boldsymbol{\alpha}})$ and γ are defined in Lemma 2.

Proof. The constraint of H is just derived from (4), so we focus on B in the remainder of the proof.

First, since we assume that the loss function L is γ-Lipschitz continuous, $L^*(\boldsymbol{\alpha})$ is known to be $(1/\gamma)$-*strongly convex* and therefore $D(\boldsymbol{\alpha})$ is $(1/\gamma)$-*strongly concave*, that is, the following holds:

$$D(\boldsymbol{v}) - D(\boldsymbol{u}) \geq \boldsymbol{g}^\top (\boldsymbol{v} - \boldsymbol{u}) + \frac{1}{2\gamma}\|\boldsymbol{v} - \boldsymbol{u}\|_2^2 \tag{13}$$

for any $\boldsymbol{u}, \boldsymbol{v} \in \mathbb{R}^n$ and any subderivative $\boldsymbol{g} \in \partial D(\boldsymbol{v})$. See[3] for example.

Substituting $\boldsymbol{u} \leftarrow \tilde{\boldsymbol{\alpha}}$ and $\boldsymbol{v} \leftarrow \boldsymbol{\alpha}^*$, we have

$$\begin{aligned} D(\boldsymbol{\alpha}^*) - D(\tilde{\boldsymbol{\alpha}}) &\geq \boldsymbol{g}^\top (\boldsymbol{\alpha}^* - \tilde{\boldsymbol{\alpha}}) + \frac{1}{2\gamma}\|\boldsymbol{\alpha}^* - \tilde{\boldsymbol{\alpha}}\|_2^2 \\ &\geq \frac{1}{2\gamma}\|\boldsymbol{\alpha}^* - \tilde{\boldsymbol{\alpha}}\|_2^2. \end{aligned} \tag{14}$$

Here, (14) is obtained by the fact that $\boldsymbol{g}^\top (\boldsymbol{\alpha}^* - \tilde{\boldsymbol{\alpha}}) \geq 0$ (See Proposition B.24 of[4] for example).

Moreover, by the calculation of the dual problem by Fenchel's duality theorem, $P(\boldsymbol{\beta}^*) = D(\boldsymbol{\alpha}^*)$ holds for (2) and (4) (called the *strong duality*; See[3] for example). Therefore, from (14) we have

$$\begin{aligned} \frac{1}{2\gamma}\|\boldsymbol{\alpha}^* - \tilde{\boldsymbol{\alpha}}\|_2^2 &\leq D(\boldsymbol{\alpha}^*) - D(\tilde{\boldsymbol{\alpha}}) = P(\boldsymbol{\beta}^*) - D(\tilde{\boldsymbol{\alpha}}) \\ &\leq P(\tilde{\boldsymbol{\beta}}) - D(\tilde{\boldsymbol{\alpha}}) \quad (\because\ \boldsymbol{\beta}^* \text{ is the minimizer of } P). \end{aligned}$$

This proves the lemma. ∎

Lemma 8. *Under the condition of the dual optimal solution $\boldsymbol{\alpha}^*$ in Lemma 7, we can represent an upper bound of $|X_{:j}^\top \boldsymbol{\alpha}^*|$ as*

$$|X_{:j}^\top \boldsymbol{\alpha}^*| \leq \max_{\boldsymbol{\alpha} \in B(\tilde{\boldsymbol{\beta}}, \tilde{\boldsymbol{\alpha}}) \cap H} |X_{:j}^\top \boldsymbol{\alpha}| = |X_{:j}^\top \tilde{\boldsymbol{\alpha}}| + r(\tilde{\boldsymbol{\beta}}, \tilde{\boldsymbol{\alpha}})\|X_{:j} - \Pi_{\mathbf{1}}(X_{:j})\|_2, \tag{15}$$

where $\Pi_{\boldsymbol{u}}(\boldsymbol{v})$ is defined in Lemma 2.

Proof. We prove this via the method of Lagrange multiplier, that is,

$$\max_{\boldsymbol{\alpha}\in B(\tilde{\boldsymbol{\beta}},\tilde{\boldsymbol{\alpha}})\cap H} X_{:j}^{\top}\boldsymbol{\alpha} = \max_{\boldsymbol{\alpha}\in\mathbb{R}^n,\xi_1\in\mathbb{R},\xi_2\in\mathbb{R}} \mathcal{L}(\boldsymbol{\alpha},\xi_1,\xi_2),$$

$$\text{where} \qquad \mathcal{L}(\boldsymbol{\alpha},\xi_1,\xi_2) = X_{:j}^{\top}\boldsymbol{\alpha} - \xi_1\left(\|\boldsymbol{\alpha}-\tilde{\boldsymbol{\alpha}}\|_2^2 - r(\tilde{\boldsymbol{\beta}},\tilde{\boldsymbol{\alpha}})^2\right) - \xi_2\boldsymbol{\alpha}^{\top}\mathbf{1}. \tag{16}$$

Let $(\boldsymbol{\alpha}^{\#},\xi_1^{\#},\xi_2^{\#}) := \arg\max_{\boldsymbol{\alpha}\in\mathbb{R}^n,\xi_1\in\mathbb{R},\xi_2\in\mathbb{R}} \mathcal{L}(\boldsymbol{\alpha},\xi_1,\xi_2)$ be the optimal solution of the latter maximization. Then it is known to satisfy the following conditions (*Karush-Kuhn-Tucker condition*):

$$\nabla L(\boldsymbol{\alpha},\xi_1,\xi_2)|_{\boldsymbol{\alpha}^{\#},\xi_1^{\#},\xi_2^{\#}} = \mathbf{0} \tag{17}$$
$$\xi_1^{\#} \geq 0 \tag{18}$$
$$\|\boldsymbol{\alpha}^{\#}-\tilde{\boldsymbol{\alpha}}\|_2^2 - r(\tilde{\boldsymbol{\beta}},\tilde{\boldsymbol{\alpha}})^2 \leq 0 \tag{19}$$
$$\xi_1^{\#}(\|\boldsymbol{\alpha}^{\#}-\tilde{\boldsymbol{\alpha}}\|_2^2 - r(\tilde{\boldsymbol{\beta}},\tilde{\boldsymbol{\alpha}})^2) = 0 \tag{20}$$
$$\xi_2^{\#} \geq 0 \tag{21}$$
$$\boldsymbol{\alpha}^{\#\top}\mathbf{1} = 0 \tag{22}$$

In this setup, it is clear that the maximization of $\mathcal{L}$ must be infinite if $\xi_1 = 0$. So, assuming $\xi_1^{\#} \neq 0$ in (18), (19) and (20), we have

$$\|\boldsymbol{\alpha}^{\#}-\tilde{\boldsymbol{\alpha}}\|_2^2 - r(\tilde{\boldsymbol{\beta}},\tilde{\boldsymbol{\alpha}})^2 = 0. \tag{23}$$

In addition, (17) is computed as follows:

$$\nabla L(\boldsymbol{\alpha},\xi_1,\xi_2)|_{\boldsymbol{\alpha}^{\#},\xi_1^{\#},\xi_2^{\#}} = X_{:j} - 2\xi_1^{\#}(\boldsymbol{\alpha}^{\#}-\tilde{\boldsymbol{\alpha}}) - \xi_2^{\#}\mathbf{1} = \mathbf{0} \tag{24}$$
$$X_{:j}^{\top}\mathbf{1} - 2\xi_1^{\#}(\boldsymbol{\alpha}^{\#}-\tilde{\boldsymbol{\alpha}})^{\top}\mathbf{1} - \xi_2^{\#}\|\mathbf{1}\|_2^2 = 0$$
$$X_{:j}^{\top}\mathbf{1} = \xi_2^{\#}\|\mathbf{1}\|_2^2 \qquad (\because \boldsymbol{\alpha}^{\#\top}\mathbf{1} = \tilde{\boldsymbol{\alpha}}^{\top}\mathbf{1} = 0)$$
$$\therefore \xi_2^{\#} = \frac{X_{:j}^{\top}\mathbf{1}}{\|\mathbf{1}\|_2^2}.$$

Moreover, substituting $\xi_2^{\#}$ in (24) we have

$$X_{:j} - 2\xi_1^{\#}(\boldsymbol{\alpha}^{\#}-\tilde{\boldsymbol{\alpha}}) - \frac{X_{:j}^{\top}\mathbf{1}}{\|\mathbf{1}\|_2^2}\mathbf{1} = X_{:j} - 2\xi_1^{\#}(\boldsymbol{\alpha}^{\#}-\tilde{\boldsymbol{\alpha}}) - \Pi_{\mathbf{1}}(X_{:j}) = \mathbf{0} \tag{25}$$
$$4(\xi_1^{\#})^2\|\boldsymbol{\alpha}^{\#}-\tilde{\boldsymbol{\alpha}}\|_2^2 = 4(\xi_1^{\#})^2 r(\tilde{\boldsymbol{\beta}},\tilde{\boldsymbol{\alpha}})^2 = \|X_{:j} - \Pi_{\mathbf{1}}(X_{:j})\|_2^2 \qquad (\because \text{(23)})$$
$$\therefore \xi_1^{\#} = \frac{\|X_{:j} - \Pi_{\mathbf{1}}(X_{:j})\|_2}{2r(\tilde{\boldsymbol{\beta}},\tilde{\boldsymbol{\alpha}})}.$$

As a result, substituting $\xi_1^{\#}$ in (25) we have

$$\boldsymbol{\alpha}^{\#} = \tilde{\boldsymbol{\alpha}} + r(\tilde{\boldsymbol{\beta}},\tilde{\boldsymbol{\alpha}})\frac{X_{:j} - \Pi_{\mathbf{1}}(X_{:j})}{\|X_{:j} - \Pi_{\mathbf{1}}(X_{:j})\|_2},$$

and

$$\max_{\boldsymbol{\alpha}\in B(\tilde{\boldsymbol{\beta}},\tilde{\boldsymbol{\alpha}})\cap H} X_{:j}^{\top}\boldsymbol{\alpha} = L(\boldsymbol{\alpha}^{\#},\xi_1^{\#},\xi_2^{\#}) = X_{:j}^{\top}\boldsymbol{\alpha}^{\#}$$
$$= [X_{:j} - \Pi_{\mathbf{1}}(X_{:j})]^{\top}\boldsymbol{\alpha}^{\#} \qquad (\because [\Pi_{\mathbf{1}}(X_{:j})]^{\top}\boldsymbol{\alpha}^{\#} = 0 \text{ by (22)})$$
$$= [X_{:j} - \Pi_{\mathbf{1}}(X_{:j})]^{\top}\tilde{\boldsymbol{\alpha}} + r(\tilde{\boldsymbol{\beta}},\tilde{\boldsymbol{\alpha}})\|X_{:j} - \Pi_{\mathbf{1}}(X_{:j})\|_2$$
$$= X_{:j}^{\top}\tilde{\boldsymbol{\alpha}} + r(\tilde{\boldsymbol{\beta}},\tilde{\boldsymbol{\alpha}})\|X_{:j} - \Pi_{\mathbf{1}}(X_{:j})\|_2. \tag{26}$$

This concludes $\max_{\boldsymbol{\alpha}\in B(\tilde{\boldsymbol{\beta}},\tilde{\boldsymbol{\alpha}})\cap H} X_{:j}^\top \boldsymbol{\alpha} = X_{:j}^\top \tilde{\boldsymbol{\alpha}} + r(\tilde{\boldsymbol{\beta}},\tilde{\boldsymbol{\alpha}})\|X_{:j} - \Pi_{\mathbf{1}}(X_{:j})\|_2$. The result consequently proves that

$$\begin{aligned}
&\max_{\boldsymbol{\alpha}\in B(\tilde{\boldsymbol{\beta}},\tilde{\boldsymbol{\alpha}})\cap H} (-X_{:j})^\top \boldsymbol{\alpha} \\
&= -X_{:j}^\top \tilde{\boldsymbol{\alpha}} + r(\tilde{\boldsymbol{\beta}},\tilde{\boldsymbol{\alpha}})\| - X_{:j} - \Pi_{\mathbf{1}}(-X_{:j})\|_2 \quad (\because\ X_{:j} \leftarrow (-X_{:j}) \text{ in (26)}) \\
&= -X_{:j}^\top \tilde{\boldsymbol{\alpha}} + r(\tilde{\boldsymbol{\beta}},\tilde{\boldsymbol{\alpha}})\|X_{:j} - \Pi_{\mathbf{1}}(X_{:j})\|_2.
\end{aligned}$$

Combining them, we have $\max_{\boldsymbol{\alpha}\in B(\tilde{\boldsymbol{\beta}},\tilde{\boldsymbol{\alpha}})\cap H} |X_{:j}^\top \boldsymbol{\alpha}| = |X_{:j}^\top \tilde{\boldsymbol{\alpha}}| + r(\tilde{\boldsymbol{\beta}},\tilde{\boldsymbol{\alpha}})\|X_{:j} - \Pi_{\mathbf{1}}(X_{:j})\|_2$. ■

Finally, Lemma 2 is proved as follows:

Proof of Lemma 2. Suppose that $u_j(\tilde{\boldsymbol{\beta}},\tilde{\boldsymbol{\alpha}}) < \lambda_1$. Then, by Lemma 8 we have

$$\begin{aligned}
\lambda > u_j(\tilde{\boldsymbol{\beta}},\tilde{\boldsymbol{\alpha}}) &:= |X_{:j}^\top \tilde{\boldsymbol{\alpha}}| + r(\tilde{\boldsymbol{\beta}},\tilde{\boldsymbol{\alpha}})\|X_{:j} - \Pi_{\mathbf{1}}(X_{:j})\|_2 \\
&\geq \max_{\boldsymbol{\alpha}\in B(\tilde{\boldsymbol{\beta}},\tilde{\boldsymbol{\alpha}})\cap H} |X_{:j}^\top \boldsymbol{\alpha}| \geq |X_{:j}^\top \boldsymbol{\alpha}^*|.
\end{aligned}$$

By equation (8), $\beta_j^* = 0$ must hold. ■

Supplemental note S2: Proof of Theorem 4

Proof. First, we prove that the SPP-score in (11) is greater than or equal to the safe screening score in (9), i.e., $v_j(\tilde{\boldsymbol{\beta}},\tilde{\boldsymbol{\alpha}}) \geq u_j(\tilde{\boldsymbol{\beta}},\tilde{\boldsymbol{\alpha}})$. This can be shown as

$$\begin{aligned}
v_j(\tilde{\boldsymbol{\beta}},\tilde{\boldsymbol{\alpha}}) - u_j(\tilde{\boldsymbol{\beta}},\tilde{\boldsymbol{\alpha}}) &= \max\left\{\sum_{i:\tilde{\alpha}_i>0} x_{ij}\tilde{\alpha}_i, -\sum_{i:\tilde{\alpha}_i<0} x_{ij}\tilde{\alpha}_i\right\} - |X_{:j}^\top \tilde{\boldsymbol{\alpha}}| + r(\tilde{\boldsymbol{\beta}},\tilde{\boldsymbol{\alpha}})\left(\|X_{:j}\|_2 - \|X_{:j} - \Pi_{\mathbf{1}}(X_{:j})\|_2\right) \\
&\geq \max\left\{\sum_{i:\tilde{\alpha}_i>0} x_{ij}\tilde{\alpha}_i, -\sum_{i:\tilde{\alpha}_i<0} x_{ij}\tilde{\alpha}_i\right\} - \max\left\{\sum_{i\in[n]} x_{ij}\alpha_i, -\sum_{i\in[n]} x_{ij}\alpha_i\right\} \\
&\geq 0.
\end{aligned}$$

Therefore, using Lemma 2, we have

$$v_j(\tilde{\boldsymbol{\beta}},\tilde{\boldsymbol{\alpha}}) < \lambda \Rightarrow u_j(\tilde{\boldsymbol{\beta}},\tilde{\boldsymbol{\alpha}}) < \lambda \Rightarrow \beta_j^* = 0.$$

Next, we prove that, for pair of pattern p_j and p_k such that $p_k \sqsubset p_j$, the SPP score of p_j is grater than or equal to that of p_k, i.e., $v_j(\tilde{\boldsymbol{\beta}},\tilde{\boldsymbol{\alpha}}) \geq v_k(\tilde{\boldsymbol{\beta}},\tilde{\boldsymbol{\alpha}})$. To show this, we prove that each of the two terms of the SPP score satisfies the intended inequality relationship, thereby showing that the SPP score as a whole also satisfies the inequality relationship. The inequality relationship for the first term of the SPP score is shown as follows. From Lemma 3, it is clear that

$$\sum_{i:\tilde{\alpha}_i>0} x_{ij}\tilde{\alpha}_i \geq \sum_{i:\tilde{\alpha}_i>0} x_{ik}\tilde{\alpha}_i.$$

Therefore, we have

$$\max\left\{\sum_{i:\tilde{\alpha}_i>0} x_{ij}\tilde{\alpha}_i, -\sum_{i:\tilde{\alpha}_i<0} x_{ij}\tilde{\alpha}_i\right\} \geq \max\left\{\sum_{i:\tilde{\alpha}_i>0} x_{ik}\tilde{\alpha}_i, -\sum_{i:\tilde{\alpha}_i<0} x_{ik}\tilde{\alpha}_i\right\}. \tag{27}$$

The inequality relationship for the second term of the SPP score is easily shown by noting that

$$\|X_{:j}\|_2 \geq \|X_{:k}\|_2.$$

This means that

$$v_j(\tilde{\boldsymbol{\beta}}, \tilde{\boldsymbol{\alpha}}) < \lambda \;\Rightarrow\; v_k(\tilde{\boldsymbol{\beta}}, \tilde{\boldsymbol{\alpha}}) < \lambda \;\Rightarrow\; \beta_k^* = 0 \;\; \forall k \in [d] \text{ s.t. } p_k \sqsubset p_j.$$

■

Supplemental note S3: Proof of Theorem 5

In order to prove the theorem, we first prove the following lemma.

Lemma 9 (Union of two hyperspheres)**.** *Suppose that two hyperspheres in $\mathbb{R}^n$, denoted by $S_1 = \{\boldsymbol{v} \in \mathbb{R}^n \mid \|\boldsymbol{v} - \boldsymbol{c}_1\|_2 = r_1\}$ and $S_2 = \{\boldsymbol{v} \in \mathbb{R}^n \mid \|\boldsymbol{v} - \boldsymbol{c}_2\|_2 = r_2\}$, satisfies $S_1 \cap S_2 \not\equiv \emptyset$ and $S_1 \not\equiv S_2$, that is,*

$$\delta := \|\boldsymbol{c}_1 - \boldsymbol{c}_2\|_2 > 0, \tag{28}$$

$$r_1 + r_2 \geq \delta, \tag{29}$$

$$|r_1 - r_2| \leq \delta. \tag{30}$$

Then, the intersection of them $S_1 \cap S_2$ is identical to the intersection $S' \cap H'$ of the following hypersphere S' and hyperplane H':

$$S' = \{\boldsymbol{v} \in \mathbb{R}^n \mid \|\boldsymbol{v} - \boldsymbol{c}'\|_2 = r'\},$$
$$H' = \{\boldsymbol{v} \in \mathbb{R}^n \mid (\boldsymbol{v} - \boldsymbol{c}')^\top (\boldsymbol{c}_1 - \boldsymbol{c}_2) = 0\},$$

where $\boldsymbol{c}'$ (center of S') and r' (radius of S') are defined as follows:

$$\boldsymbol{c}' = t\boldsymbol{c}_1 + (1-t)\boldsymbol{c}_2,$$
$$r' = \sqrt{r_2^2 - t^2\delta^2},$$
$$t = \frac{1}{2}\left(1 + \frac{r_2^2 - r_1^2}{\delta^2}\right).$$

Proof of Lemma 9. Let $E : \mathbb{R}^n \to \mathbb{R}^n$ be an distance-preserving mapping such that

$$E\boldsymbol{c}_1 = \boldsymbol{0},$$
$$E\boldsymbol{c}_2 = [\delta, \underbrace{0, \ldots, 0}_{n-1}]^\top,$$

where $\delta = \|\boldsymbol{c}_1 - \boldsymbol{c}_2\|_2$. Note that such a mapping can be obtained as follows:

- Let $E\boldsymbol{v} := \Theta(\boldsymbol{v} - \boldsymbol{c}_1)$ ($\Theta \in \mathbb{R}^{n \times n}$).
- Set $\Theta_{1:} = \frac{1}{\delta^2}(\boldsymbol{c}_1 - \boldsymbol{c}_2)$.
- Set other rows of Θ so that Θ is an orthogonal matrix. This can be done by Gram-Schmidt algorithm.

Let $\boldsymbol{v} \in S_1 \cap S_2$, and $\boldsymbol{v}'$ be

$$E\boldsymbol{v} = \boldsymbol{v}' = [v'_1, \dots, v'_n]^\top.$$

Then, since E is distance-preserving, we have

$$\|E\boldsymbol{v} - E\boldsymbol{c}_1\|_2^2 = r_1^2 \iff \sum_{i=1}^{n} v_i'^2 = r_1^2, \tag{31}$$

$$\|E\boldsymbol{v} - E\boldsymbol{c}_2\|_2^2 = r_2^2 \iff \sum_{i=2}^{n} v_i'^2 + (v'_1 - \delta)^2 = r_2^2. \tag{32}$$

Taking the difference between equations (31) and (32), we have

$$v'_1 = \frac{r_1^2 - r_2^2 + \delta^2}{2\delta}, \tag{33}$$

$$\sum_{i=2}^{n} v_i'^2 = r_2^2 - (v'_1 - \delta)^2 = r_2^2 - \left(\frac{r_1^2 - r_2^2 - \delta^2}{2\delta}\right)^2 = r_2^2 - t^2\delta^2. \tag{34}$$

Note that the value (34) is nonnegative because

$$\begin{aligned} r_2^2 - \left(\frac{r_1^2 - r_2^2 - \delta^2}{2\delta}\right)^2 &= \frac{1}{2\delta}(2\delta r_2 + r_1^2 - r_2^2 - \delta^2)(2\delta r_2 - r_1^2 + r_2^2 + \delta^2) \\ &= \frac{1}{2\delta}[r_1^2 - (r_2 - \delta)^2][(r_2 + \delta)^2 - r_1^2] \\ &= \frac{1}{2\delta}\underbrace{(r_1 + r_2 - \delta)}_{\geq 0 \because (29)}\underbrace{(r_1 - r_2 + \delta)}_{\geq 0 \because (30)}\underbrace{(r_1 + r_2 + \delta)}_{> 0 \text{ clearly}}\underbrace{(-r_1 + r_2 + \delta)}_{\geq 0 \because (30)}. \end{aligned}$$

In summary, equation (33) implies that v'_1 of $\boldsymbol{v}'$ is constant. In addition, equation (34) implies that $[v'_2, \dots, v'_n]$ is on a hypersphere whose center is $[v'_2, \dots, v'_n]^\top = [\underbrace{0, \dots, 0}_{n-1}]^\top$. So, if we take a hypersphere whose center is $E\boldsymbol{c}' = [v'_1, \underbrace{0, \dots, 0}_{n-1}]^\top$ and whose radius is $r' = \sqrt{r_2^2 - t^2\delta^2}$, then the intersection of it and the hyperplane $v'_1 = \frac{r_1^2 - r_2^2 + \delta^2}{2\delta}$ composes $\boldsymbol{v}'$.

Finally we derive the center of the hypersphere in the original space $\boldsymbol{c}'$. (Note that r' is the same between in the original space and the space after applying E, since E is distance-preserving.) Again, in the space after applying E,

$$E\boldsymbol{c}_1 = [0, \underbrace{0, \dots, 0}_{n-1}]^\top, \quad E\boldsymbol{c}' = [v'_1, \underbrace{0, \dots, 0}_{n-1}]^\top, \quad E\boldsymbol{c}_2 = [\delta, \underbrace{0, \dots, 0}_{n-1}]^\top.$$

Since E is distance-preserving, $\boldsymbol{c}'$ in the original space can be computed as

$$\begin{aligned} \boldsymbol{c}' &= \boldsymbol{c}_1 + \frac{v'_1}{\delta}(\boldsymbol{c}_2 - \boldsymbol{c}_1) \\ &= \boldsymbol{c}_1 + \frac{r_1^2 - r_2^2 + \delta^2}{2\delta^2}(\boldsymbol{c}_2 - \boldsymbol{c}_1) \\ &= \frac{\delta^2 - r_1^2 + r_2^2}{2\delta^2}\boldsymbol{c}_1 + \left(1 - \frac{\delta^2 - r_1^2 + r_2^2}{2\delta^2}\right)\boldsymbol{c}_2 = t\boldsymbol{c}_1 + (1 - t)\boldsymbol{c}_2. \end{aligned}$$

This derives $\boldsymbol{c}'$ and t in the lemma. ■

Proof of Theorem 5. In order to prove

$$\max_{\boldsymbol{\alpha}\in B_1\cap B_2\cap H} |X_{:j}^\top \boldsymbol{\alpha}| = \max\{u_j^+, u_j^-\},$$

first we note that

$$\max_{\boldsymbol{\alpha}} |X_{:j}^\top \boldsymbol{\alpha}| = \max\{\max_{\boldsymbol{\alpha}} X_{:j}^\top \boldsymbol{\alpha}, \max_{\boldsymbol{\alpha}} (-X_{:j})^\top \boldsymbol{\alpha}\}.$$

The second expression can be obtained by just replacing $X_{:j}$ with $-X_{:j}$ in the first expression. So we discuss only the first expression, that is,

$$\max_{\boldsymbol{\alpha}\in B_1\cap B_2\cap H} X_{:j}^\top \boldsymbol{\alpha}. \tag{35}$$

The Lagrangian function of (35) is defined as

$$\mathcal{L}(\boldsymbol{\alpha}, \xi_1, \xi_2, \xi_3) = X_{:j}^\top \boldsymbol{\alpha} - \xi_1(\|\boldsymbol{\alpha} - \tilde{\boldsymbol{\alpha}}^{(1)}\|_2^2 - r(R_1)^2) - \xi_2(\|\boldsymbol{\alpha} - \tilde{\boldsymbol{\alpha}}^{(2)}\|_2^2 - r(R_2)^2) - \xi_3 \boldsymbol{\alpha}^\top \mathbf{1}.$$

Then, the optimal solution of (35), denoted by $\tilde{\boldsymbol{\alpha}}^*$, must satisfy the following conditions:

$$\nabla_{\boldsymbol{\alpha}} \mathcal{L}|_{\boldsymbol{\alpha}=\tilde{\boldsymbol{\alpha}}^*} = \mathbf{0}, \tag{36}$$

$$\xi_1 \geq 0, \tag{37}$$

$$\|\tilde{\boldsymbol{\alpha}}^* - \tilde{\boldsymbol{\alpha}}^{(1)}\|_2^2 - r(R_1)^2 \leq 0, \tag{38}$$

$$\xi_1(\|\tilde{\boldsymbol{\alpha}}^* - \tilde{\boldsymbol{\alpha}}^{(1)}\|_2^2 - r(R_1)^2) = 0, \tag{39}$$

$$\xi_2 \geq 0, \tag{40}$$

$$\|\tilde{\boldsymbol{\alpha}}^* - \tilde{\boldsymbol{\alpha}}^{(2)}\|_2^2 - r(R_2)^2 \leq 0, \tag{41}$$

$$\xi_2(\|\tilde{\boldsymbol{\alpha}}^* - \tilde{\boldsymbol{\alpha}}^{(2)}\|_2^2 - r(R_2)^2) = 0, \tag{42}$$

$$\boldsymbol{\alpha}^\top \mathbf{1} = 0. \tag{43}$$

Note that, if $\xi_1 = \xi_2 = 0$, (i.e., neither (38) nor (41) are active), then $\max_{\boldsymbol{\alpha}} \mathcal{L}$ cannot be finite. So we can assume that $(\xi_1, \xi_2) \neq (0, 0)$. From (36), we have

$$\nabla_{\boldsymbol{\alpha}} \mathcal{L} = X_{:j} - 2\xi_1(\boldsymbol{\alpha} - \tilde{\boldsymbol{\alpha}}^{(1)}) - 2\xi_2(\boldsymbol{\alpha} - \tilde{\boldsymbol{\alpha}}^{(2)}) - \xi_3 \mathbf{1}.$$

Since $\xi_1 + \xi_2 \neq 0$, we have

$$\tilde{\boldsymbol{\alpha}}^* = \frac{1}{2(\xi_1 + \xi_2)} \left(X_{:j} + 2\xi_1 \tilde{\boldsymbol{\alpha}}^{(1)} + 2\xi_2 \tilde{\boldsymbol{\alpha}}^{(2)} - \xi_3 \mathbf{1} \right).$$

Moreover, since $\mathbf{1}^\top \tilde{\boldsymbol{\alpha}}^{(1)} = \mathbf{1}^\top \tilde{\boldsymbol{\alpha}}^{(2)} = 0$ from (43), we have

$$\frac{1}{2(\xi_1 + \xi_2)} \left(X_{:j}^\top \mathbf{1} - \xi_3 \mathbf{1}^\top \mathbf{1} \right) = 0$$

$$\therefore \xi_3 = \frac{X_{:j}^\top \mathbf{1}}{\mathbf{1}^\top \mathbf{1}}.$$

Here we calculate the solution $\tilde{\boldsymbol{\alpha}}^*$ based on the values of ξ_1 and ξ_2. First, if $\xi_1 \neq 0$ and $\xi_2 = 0$, (i.e., (38) is active but not (41)), then we have $\|\boldsymbol{\alpha} - \tilde{\boldsymbol{\alpha}}^{(1)}\|_2^2 = r(R_1)^2$ and

$$\frac{1}{4\xi_1^2} \|X_{:j} - \Pi_{\mathbf{1}}(X_{:j})\|_2^2 = r(R_1)^2$$

$$\therefore \xi_1 = \frac{\|X_{:j} - \Pi_{\mathbf{1}}(X_{:j})\|_2}{r(R_1)},$$

then

$$\tilde{\boldsymbol{\alpha}}^* = \frac{r(R_1)}{\|X_{:j} - \Pi_{\mathbf{1}}(X_{:j})\|_2}\,(X_{:j} - \Pi_{\mathbf{1}}(X_{:j})) + \tilde{\boldsymbol{\alpha}}^{(1)},$$

and the maximized result is calculated as

$$\begin{aligned}\mathcal{L}(\tilde{\boldsymbol{\alpha}}^*, \xi_1, \xi_2, \xi_3) &= X_{:j}^\top \tilde{\boldsymbol{\alpha}}^* \\ &= (X_{:j} - \Pi_{\mathbf{1}}(X_{:j}))^\top \tilde{\boldsymbol{\alpha}}^* \\ &= X_{:j}^\top \tilde{\boldsymbol{\alpha}}^{(1)} + r(R_1)\|X_{:j} - \Pi_{\mathbf{1}}(X_{:j})\|_2. \end{aligned} \tag{44}$$

In this case $\tilde{\boldsymbol{\alpha}}^* \in B_2$ must hold, that is, $\|\tilde{\boldsymbol{\alpha}}^* - \tilde{\boldsymbol{\alpha}}^{(2)}\|_2^2 \leq r(R_2)^2$. So we have

$$\frac{X_{:j}^\top \boldsymbol{\delta}}{\|X_{:j} - \Pi_{\mathbf{1}}(X_{:j})\|_2} \leq \frac{r(R_2)^2 - r(R_1)^2 - \|\boldsymbol{\delta}\|_2^2}{2r(R_1)}.$$

If $\xi_1 = 0$ and $\xi_2 \neq 0$, the calculation can be done similarly: we can conclude that

$$\tilde{\boldsymbol{\alpha}}^* = \frac{r(R_2)}{\|X_{:j} - \Pi_{\mathbf{1}}(X_{:j})\|_2}\,(X_{:j} - \Pi_{\mathbf{1}}(X_{:j})) + \tilde{\boldsymbol{\alpha}}^{(2)}$$

and

$$\mathcal{L}(\tilde{\boldsymbol{\alpha}}^*, \xi_1, \xi_2, \xi_3) = X_{:j}^\top \tilde{\boldsymbol{\alpha}}^{(2)} + r(R_2)\|X_{:j} - \Pi_{\mathbf{1}}(X_{:j})\|_2. \tag{45}$$

Since $\tilde{\boldsymbol{\alpha}}^* \in B_1$, we also have

$$\frac{X_{:j}^\top \boldsymbol{\delta}}{\|X_{:j} - \Pi_{\mathbf{1}}(X_{:j})\|_2} \geq \frac{r(R_2)^2 - r(R_1)^2 + \|\boldsymbol{\delta}\|_2^2}{2r(R_2)}.$$

Finally we show the case of $\xi_1 \neq 0$ and $\xi_2 \neq 0$. In this case, since $\|\boldsymbol{\alpha} - \tilde{\boldsymbol{\alpha}}^{(1)}\|_2^2 = r(R_1)^2$ and $\|\boldsymbol{\alpha} - \tilde{\boldsymbol{\alpha}}^{(2)}\|_2^2 = r(R_2)^2$, the constraint can be represented as an intersection of two hyperspheres in $\mathbb{R}^n$. So we replace them with Lemma 9. Let S_1 and S_2 be the surfaces of B_1 and B_2, respectively. Then the problem is rewritten as:

$$\max_{\boldsymbol{\alpha} \in S_1 \cap S_2 \cap H} X_{:j}^\top \boldsymbol{\alpha} = \max_{\boldsymbol{\alpha} \in S' \cap H' \cap H} X_{:j}^\top \boldsymbol{\alpha},$$

where

$$\begin{aligned} S' &= \{\boldsymbol{v} \in \mathbb{R}^n \mid \|\boldsymbol{v} - \tilde{\boldsymbol{\alpha}}'\| < r'\}, \\ H' &= \{\boldsymbol{v} \in \mathbb{R}^n \mid (\boldsymbol{v} - \tilde{\boldsymbol{\alpha}}')^\top \boldsymbol{\delta} = 0\}, \end{aligned}$$

and $\tilde{\boldsymbol{\alpha}}', r', \boldsymbol{\delta}$ are the ones defined in Theorem 5. Its Lagrangian function $\mathcal{L}'$ is defined as

$$\mathcal{L}'(\boldsymbol{\alpha}, \xi_1', \xi_2', \xi_3') = X_{:j}^\top \boldsymbol{\alpha} - \xi_1'(\|\boldsymbol{\alpha} - \tilde{\boldsymbol{\alpha}}'\|_2^2 - r'^2) - \xi_2'(\boldsymbol{\alpha} - \tilde{\boldsymbol{\alpha}})^\top \boldsymbol{\delta} - \xi_3' \boldsymbol{\alpha}^\top \mathbf{1},$$

with the optimality conditions

$$\nabla_{\boldsymbol{\alpha}} \mathcal{L}|_{\boldsymbol{\alpha} = \tilde{\boldsymbol{\alpha}}^*} = \mathbf{0}, \tag{46}$$

$$\|\tilde{\boldsymbol{\alpha}}^* - \tilde{\boldsymbol{\alpha}}'\|_2^2 - r' = 0, \tag{47}$$

$$(\tilde{\boldsymbol{\alpha}}^* - \tilde{\boldsymbol{\alpha}}')^\top \boldsymbol{\delta} = 0, \tag{48}$$

$$\tilde{\boldsymbol{\alpha}}^{*\top} \mathbf{1} = 0. \tag{49}$$

Noticing that $\xi_1 \neq 0$ (otherwise $\max_{\boldsymbol{\alpha}} \mathcal{L}'$ is not bounded), from (46) we have

$$\nabla \mathcal{L} = X_{:j} - 2\xi_1'(\boldsymbol{\alpha} - \tilde{\boldsymbol{\alpha}}') - \xi_2' \boldsymbol{\delta} - \xi_3' \mathbf{1}$$

and

$$\tilde{\boldsymbol{\alpha}}^* = \tilde{\boldsymbol{\alpha}}' + \frac{1}{2\xi_1'} \left(\xi_2' \boldsymbol{\delta} + X_{:j} - \xi_3' \mathbf{1} \right).$$

Since $\mathbf{1}^\top \boldsymbol{\delta} = 0$, from (49) we have

$$\xi_3' = \frac{X_{:j}^\top \mathbf{1}}{\|\mathbf{1}\|_2^2}$$

and from (48) we have

$$\xi_2' = -\frac{X_{:j}^\top \boldsymbol{\delta}}{\|\boldsymbol{\delta}\|_2^2}.$$

Then, from (47) we have

$$\xi_1' = \frac{\|X_{:j} - \Pi_{\mathbf{1}}(X_{:j}) - \Pi_{\boldsymbol{\delta}}(X_{:j})\|_2^2}{2r'}.$$

Since $\boldsymbol{\alpha} \in H$ and $\boldsymbol{\alpha} \in H'$, we have $\boldsymbol{\alpha}^\top \Pi_{\mathbf{1}}(X_{:j}) = 0$ and $\boldsymbol{\alpha}^\top \Pi_{\boldsymbol{\delta}}(X_{:j}) = \tilde{\boldsymbol{\alpha}}^\top \Pi_{\boldsymbol{\delta}}(X_{:j})$, respectively. Thus we can conclude that

$$\begin{aligned}
\mathcal{L}^*(\tilde{\boldsymbol{\alpha}}^*, \xi_1', \xi_2', \xi_3') &= X_{:j}^\top \tilde{\boldsymbol{\alpha}}^* \\
&= (X_{:j} - \Pi_{\mathbf{1}}(X_{:j}) - \Pi_{\boldsymbol{\delta}}(X_{:j}))^\top \tilde{\boldsymbol{\alpha}}^* + \tilde{\boldsymbol{\alpha}}'^\top \Pi_{\mathbf{1}}(X_{:j}) \\
&= X_{:j}^\top \tilde{\boldsymbol{\alpha}}' + r' \|X_{:j} - \Pi_{\mathbf{1}}(X_{:j}) - \Pi_{\boldsymbol{\delta}}(X_{:j})\|_2^2.
\end{aligned}$$

■

Supplemental note S4: Algorithms

We present Algorithms 1 to 4 described in this paper.

Algorithm 1 Safe Pattern Pruning

Input: $X, \boldsymbol{y}, \lambda, \kappa, R = (\tilde{\boldsymbol{\beta}}, \tilde{\boldsymbol{\alpha}})$
Output: $\mathcal{A}$
  $\mathcal{A} \leftarrow \emptyset$
  $\mathcal{P} \leftarrow \{\emptyset\}$
  while $\mathcal{P} \neq \emptyset$ **do**
    Pop from the top of $\mathcal{P}$ as p
    Enumerate expanded patterns $\mathcal{P}'$ from p
    for $p_j \in \mathcal{P}'$ **do**
      if $v_j(R) < \lambda$ **then**
        comtinue
      end if
      if $u_j(R) \geq \lambda$ **then**
        $\mathcal{A} \leftarrow \mathcal{A} \cup \{j\}$
      end if
      Push p' into the top of $\mathcal{P}$
    end for
  end while

Algorithm 2 Pathwise optimization with SPP

Input: $X, \boldsymbol{y}, \{(\lambda^{(k)}, \kappa^{(k)})\}_{k \in [K]}, \epsilon$
Output: $\{\boldsymbol{\beta}^{*(k)}\}_{k \in [K]}$
  $\boldsymbol{\beta} \leftarrow \mathbf{0}$
  for $k \in [K]$ **do**
    $\lambda \leftarrow \lambda^{(k)}$
    $\kappa \leftarrow \kappa^{(k)}$
    Compute $\boldsymbol{\alpha}$ from $\boldsymbol{\beta}$ by the dual scaling
    $R \leftarrow (\boldsymbol{\beta}, \boldsymbol{\alpha})$
    $\mathcal{A} \leftarrow \mathrm{SafePatternPruning}(X, \boldsymbol{y}, \lambda, \kappa, R)$
    while $true$ **do**
      Update $\boldsymbol{\beta}$ using the sub-gradient of P
      Copute $\boldsymbol{\alpha}$ from $\boldsymbol{\beta}$ by hte dual scaling
      $R \leftarrow (\boldsymbol{\beta}, \boldsymbol{\alpha})$
      if $G(R) < \epsilon$ **then**
        $\boldsymbol{\beta}^{*(k)} \leftarrow \boldsymbol{\beta}$
        break
      end if
      Remove inactive patterns from $\mathcal{A}$ using safe screening
    end while
  end for

Algorithm 3 Pathwise optimization with multi-reference SPP

Input: $X, \boldsymbol{y}, \{\lambda^{(k)}\}_{k\in[K]}, \{\kappa^{(k')}\}_{k'\in[K']}, \epsilon, M$
Output: $\{R^{(k,k')}\}_{(k,k')\in[K]\times[K']}$

```
for k ∈ [K] do
  for k' ∈ [K'] do
    λ ← λ^(k)
    κ ← κ^(k')
    𝓡 ← ∅
    if k > 1 then
      𝓡 ← 𝓡 ∪ {R^(k−1,k')}
    end if
    if k' > 1 then
      𝓡 ← 𝓡 ∪ {R^(k,k'−1)}
    end if
    if 𝓡 = ∅ then
      𝓡 ← 𝓡 ∪ {(0, 0)}
    end if
    for R ∈ 𝓡 do
      β, α ← R
      Update α from β by dual scaling
    end for
    𝓐 ← MultiSafePatternPruning(X, y, λ, κ, 𝓡)
    for m ∈ {1, 2, ...} do
      for R ∈ 𝓡 do
        β, α ← R
        Update β of R using the sub-gradient of P
        Update α from β using the dual scaling
        R ← (β, α)
      end for
      if min_{R∈𝓡} G(R) < ε then
        β*^(k,k') ← β
        break
      end if
      Remove inactive patterns from 𝓐 using multi safe screening
      if m ≥ M then
        𝓡 ← {argmin_{R∈𝓡} G(R)}
      end if
    end for
  end for
end for
```

Algorithm 4 Pathwise optimization in cross-validation with multi-reference SPP

Input: $X, \boldsymbol{y}, \{\mathcal{I}^{(k)}\}_{k\in[K]}, \{\lambda^{(k')}\}_{k'\in[K']}, \kappa, \epsilon, M$
Output: $\{R^{(k,k')}\}_{(k,k')\in[K]\times[K']}$
  for $k \in [K]$ **do**
    $X' \leftarrow (X^\top_{\mathcal{I}^{(k)}})^\top$
    $\boldsymbol{y}' \leftarrow \boldsymbol{y}_{\mathcal{I}^{(k)}}$
    for $k' \in [K']$ **do**
      $\lambda \leftarrow \lambda^{(k')}$
      $\mathcal{R} \leftarrow \emptyset$
      if $k > 1$ **then**
        $\mathcal{R} \leftarrow \mathcal{R} \cup \{R^{(1,k')}\}$
      end if
      if $k' > 1$ **then**
        $\mathcal{R} \leftarrow \mathcal{R} \cup \{R^{(k,k'-1)}\}$
      end if
      if $\mathcal{R} = \emptyset$ **then**
        $\mathcal{R} \leftarrow \mathcal{R} \cup \{(\mathbf{0}, \mathbf{0})\}$
      end if
      for $R \in \mathcal{R}$ **do**
        $\boldsymbol{\beta}, \boldsymbol{\alpha} \leftarrow R$
        Update $\boldsymbol{\alpha}$ from $\boldsymbol{\beta}$ by dual scaling
      end for
      $\mathcal{A} \leftarrow \mathrm{MultiSafePatternPruning}(X', \boldsymbol{y}', \lambda, \kappa, \mathcal{R})$
      for $m \in \{1, 2, \ldots\}$ **do**
        for $R \in \mathcal{R}$ **do**
          $\boldsymbol{\beta}, \boldsymbol{\alpha} \leftarrow R$
          Update $\boldsymbol{\beta}$ of R using the sub-gradient of P
          Update $\boldsymbol{\alpha}$ from $\boldsymbol{\beta}$ using the dual scaling
          $R \leftarrow (\boldsymbol{\beta}, \boldsymbol{\alpha})$
        end for
        if $\min_{R\in\mathcal{R}} G(R) < \epsilon$ **then**
          $\boldsymbol{\beta}^{*(k,k')} \leftarrow \boldsymbol{\beta}$
          break
        end if
        Remove inactive patterns from $\mathcal{A}$ using multi safe screening
        if $m \geq M$ **then**
          $\mathcal{R} \leftarrow \{\mathrm{argmin}_{R\in\mathcal{R}} G(R)\}$
        end if
      end for
    end for
  end for

References

1. Fercoq, O., Gramfort, A., and Salmon, J. Mind the duality gap: safer rules for the lasso. In: *International Conference on Machine Learning*. PMLR (2015):(333–342). doi:https://dl.acm.org/doi/10.5555/3045118.3045155.

2. Ndiaye, E., Fercoq, O., Gramfort, A., and Salmon, J. (2017). Gap safe screening rules for sparsity enforcing penalties. The Journal of Machine Learning Research *18*, 4671–4703. doi:https://dl.acm.org/doi/abs/10.5555/3122009.3208009.

3. Rockafellar, R. T., and Wets, R. J.-B. Variational analysis chap. §11.H “Dual Problems of Optimization”, §11.I “Lagrangian Functions”, §12.H “Strong Monotonicity and Strong Convexity”. Springer Science & Business Media (2009):.

4. Bertsekas, D. P. Nonlinear Programming chap. §B: “Convex Analysis”. Athena Scientific 2nd ed. (1999):.
