# Supplementary material for: Efficient model selection for predictive pattern mining model by safe pattern pruning
Source: Patterns (N Y). 2023 Dec 1;4(12):100890. doi: 10.1016/j.patter.2023.100890 (PMC10724371; doi:10.1016/j.patter.2023.100890)
Supplement: Document S2. Article plus supplemental information [file mmc2.pdf]

Patterns

Article

Efficient model selection for predictive pattern mining model by safe pattern pruning

Graphical abstract

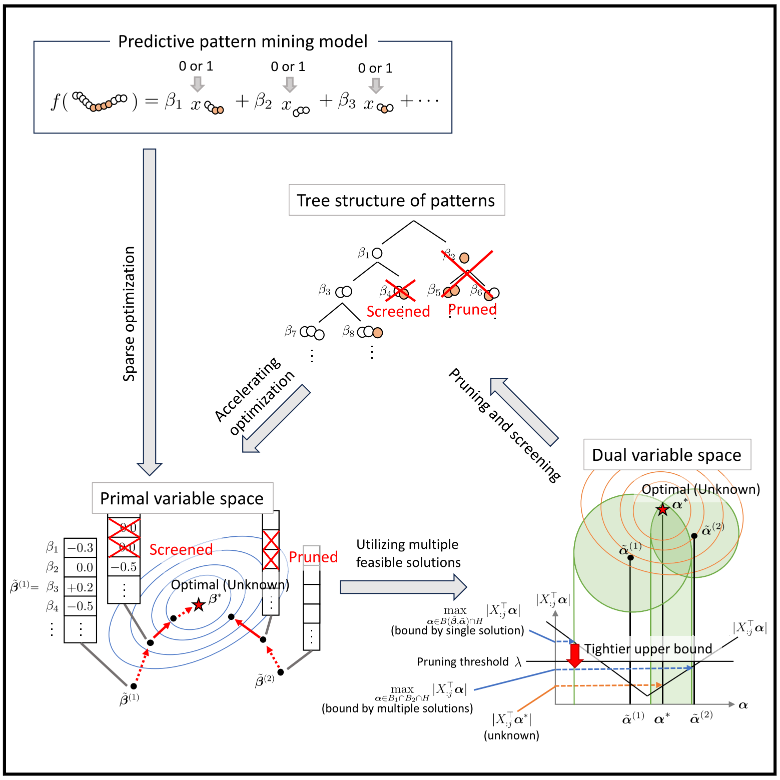

Highlights

- Predictive pattern mining is used for interpretable modeling of complex structures
- Safe pattern pruning (SPP) can solve this problem efficiently
- Utilizing multiple feasible solutions reduces further computational costs of SPP

Authors

Takumi Yoshida, Hiroyuki Hanada, Kazuya Nakagawa, Kouichi Taji, Koji Tsuda, Ichiro Takeuchi

Correspondence

hiroyuki.hanada@riken.jp (H.H.),
ichiro.takeuchi@mae.nagoya-u.ac.jp (I.T.)

In brief

Predictive pattern mining is an approach to modeling the relationship between complex structures such as amino acid sequences or molecular graphs and their numerical properties or class labels using their unified inconsistent notation sub-structures. Yoshida et al. present a method (safe pattern pruning [SPP]) that uses safe screening and the tree structure of patterns. SPP overcomes the computational drawback of existing strategies and reduces further computation costs by utilizing multiple feasible solutions from the optimization problem for obtaining the predictive model.

Yoshida et al., 2023, Patterns *4*, 100890
December 8, 2023 © 2023 The Author(s).
https://doi.org/10.1016/j.patter.2023.100890

CellPress

Patterns

CellPress
OPEN ACCESS

Article

Efficient model selection for predictive pattern mining model by safe pattern pruning

Takumi Yoshida,[1,5] Hiroyuki Hanada,[2,*] Kazuya Nakagawa,[1] Kouichi Taji,[3] Koji Tsuda,[2,4] and Ichiro Takeuchi[2,3,6,*]
[1]Department of Engineering, Nagoya Institute of Technology, Nagoya, Aichi 466-8555, Japan
[2]Center for Advanced Intelligence Project, RIKEN, Tokyo 103-0027, Japan
[3]Department of Mechanical Systems Engineering, Nagoya University, Nagoya, Aichi 464-8603, Japan
[4]Department of Bioinformatics and Systems Biology, The University of Tokyo, Bunkyo-ku, Tokyo 113-0033, Japan
[5]Present address: Department of Mechanical Systems Engineering, Nagoya University, Nagoya, Aichi 464-8603, Japan
[6]Lead contact
*Correspondence: hiroyuki.hanada@riken.jp (H.H.), ichiro.takeuchi@mae.nagoya-u.ac.jp (I.T.)
https://doi.org/10.1016/j.patter.2023.100890

THE BIGGER PICTURE Data-driven approaches to predicting properties or classifying instances represented as complex structures (e.g., sequences and graphs) are highly important. For example, the relationship between organic molecules and their chemical properties can be modeled using graphs as inputs, where nodes represent atoms and edges represent chemical bonds. Similarly, the classification of proteins is achieved by models that predict class probabilities from sequences of amino acid residues. While deep neural networks have been widely studied recently, they often lack explainability; that is, they are difficult to interpret and monitor by humans. Thus, the development of more interpretable and resource efficient methods for such data remains an essential challenge.

1 2 3 4 5 **Concept:** Basic principles of a new data science output observed and reported

SUMMARY

Predictive pattern mining is an approach used to construct prediction models when the input is represented by structured data, such as sets, graphs, and sequences. The main idea behind predictive pattern mining is to build a prediction model by considering unified inconsistent notation sub-structures, such as subsets, subgraphs, and subsequences (referred to as patterns), present in the structured data as features of the model. The primary challenge in predictive pattern mining lies in the exponential growth of the number of patterns with the complexity of the structured data. In this study, we propose the safe pattern pruning method to address the explosion of pattern numbers in predictive pattern mining. We also discuss how it can be effectively employed throughout the entire model building process in practical data analysis. To demonstrate the effectiveness of the proposed method, we conduct numerical experiments on regression and classification problems involving sets, graphs, and sequences.

INTRODUCTION

In various practical problems, it is necessary to handle structure data such as sets, graphs, and sequences. For example, in the field of life sciences, interactions between different genes are represented as sets, drug-able chemical compounds are represented as graphs, and amino acid sequences that make up proteins are represented as sequence data. In this paper, we consider prediction problems such as regression and classification when the input is structure data. In the aforementioned cases, problems such as predicting the presence or absence of a disease based on interactions between genes, predicting the effectiveness of drugs based on chemical compound structures, and predicting allergic reactions based on the amino acid sequences of food proteins are examples of our target applications. In predictive modeling for structure data, the challenge is how to represent the structure data so that they can be fed into machine learning framework. In this paper, we consider a class of machine learning models called predictive pattern mining.[1–14]

There are mainly three types of machine learning approaches for structure data. The first approach is the kernel-based approach. In this approach, a kernel function that can quantify

 © 2023 The Author(s).
This is an open access article under the CC BY license (http://creativecommons.org/licenses/by/4.0/).

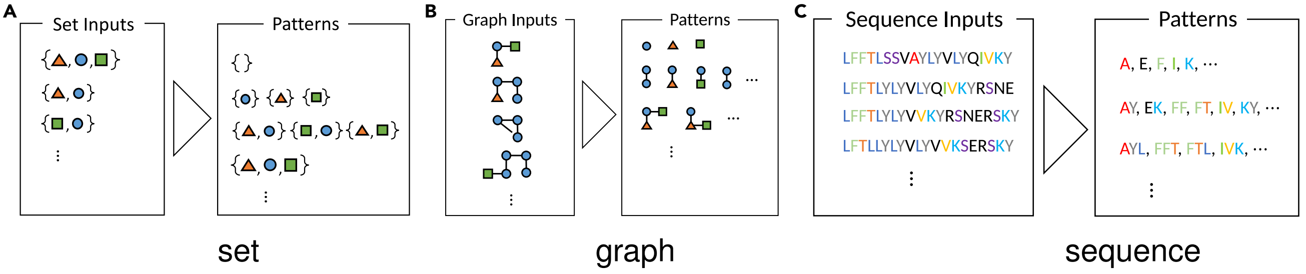

Figure 1. Examples of patterns for each type of structure
Examples of patterns (sub-structures) of (A) set data, (B) graph data, and (C) sequence data.

similarities between sets, graphs, sequences, and other structures are introduced, and it is used together with kernel-based machine learning methods such as support vector machines and Gaussian processes. A variety of kernel functions specialized for each type of structures have been proposed and used in practical problems.[15–21] The second approach is deep learning-based approach. This approach employs neural network models with special types of input and hidden layers that are designed to handle the inputs in the form of sets, graphs, sequences, and other structures. For example, PointNet is a neural network especially developed for set data,[22,23] graph neural networks are used for graph data,[24–28] and there are many neural network architectures for sequence data such as recurrent neural networks and LSTM.[29–32] These two approaches are effective when we are only interested in prediction. In many practical problems, however, simply having good predictive performance is not enough. In prediction modeling for structure data, knowledge extraction such as identifying the sub-structures that contribute to the prediction, is required for the explanation and interpretation.

The third approach is predictive pattern mining, which is the subject of this paper. In contrast with the two aforementioned approaches, knowledge extraction is possible in a predictive pattern mining approach. A common feature among many types of structure data such as sets, graphs, and sequences is that they can be decomposed into sub-structures. For example, considering a set of three genes $\{g_A, g_B, g_C\}$ as a set data, it has the following sub-structures:

$$\emptyset, \{g_A\}, \{g_B\}, \{g_C\}, \{g_A, g_B\}, \{g_A, g_C\}, \{g_B, g_C\}, \{g_A, g_B, g_C\},$$

where $\emptyset$ is the empty set. If we consider a predictive model that takes a set as an input, it is possible to extract knowledge by knowing which sub-structures contribute significantly to the prediction. In this paper, these unified inconsistent notation sub-structures are called patterns. Figure 1 shows examples of patterns for set, graph, and sequence data.

The difficulty in predictive pattern mining lies in the computational complexity of efficiently handling an exponentially increasing number of patterns. In any of the set, graph, or sequence structure data discussed so far, the number of all possible sub-structures (patterns) are huge, making it difficult to consider a machine learning model that naively treats all possible patterns as features. Since the patterns that affect predictions are often only a small part of the vast number of pattern features, the basic strategy for predictive pattern mining is to efficiently identify relevant pattern features and remove irrelevant pattern features that do not affect predictions. In the field of pattern mining, algorithms utilizing the fact that patterns can be represented in a tree structure have been exploited for tasks such as enumerating frequently occurring patterns (Figure 2). Our main contribution in this paper is to propose a method for efficiently finding patterns that significantly contribute to predictions by using the tree representation of patterns, similar to other pattern mining methods.

To this end, we propose a technique called safe pattern pruning (SPP) by combining safe screening, which has been developed in the field of sparse modeling, and pattern mining that utilizes tree-based pattern representations. We show the overview of our method in Figure 3. In the SPP method, we consider the sparse estimation of linear models that can have any pattern as a feature for predictive pattern mining model and identify a specific set of patterns where the coefficients become zero in the optimal solution.

This paper is an extended version of the preliminary conference proceeding presented by a part of the authors.[33] The

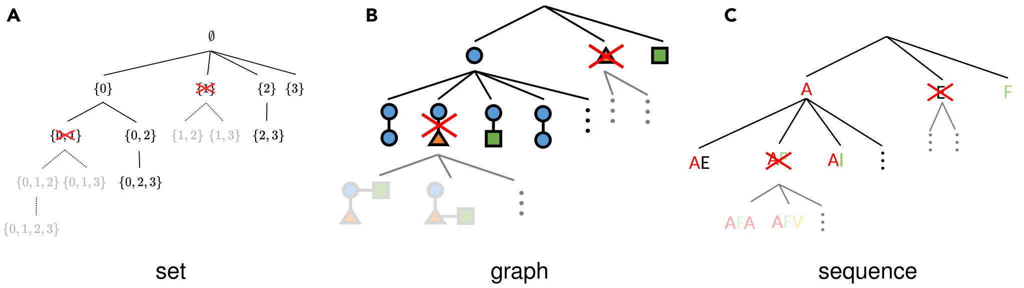

Figure 2. Pruning in the search for patterns of each type of structure
Conceptual diagram of pruning in the search for patterns of (A) sets, (B) graphs, and (C) sequences represented by a tree.

CellPress
OPEN ACCESS

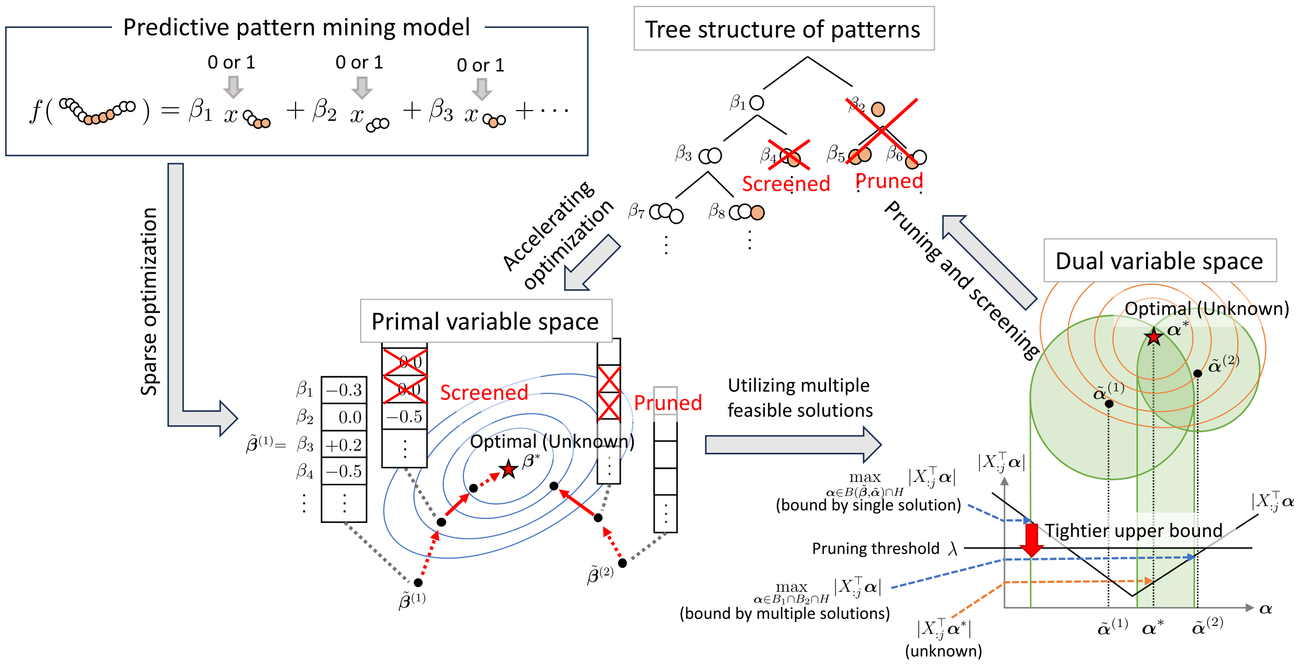

Figure 3. Overview of our method

It aims to solve predictive pattern mining problems utilizing safe screening. We enhanced the screening performance by exploiting the tree structure of patterns and the multiple feasible solutions of the optimization problem.

entire content of "SPP for model selection" is an addition to this paper, and a significant portion of the experiments described in "Numerical experiments" were newly conducted in this paper. In this paper, we newly introduce methods to effectively perform model selection by effectively using the SPP; provide software that can comprehensively handle regression and classification problems with sets, graphs, and sequences; and present new numerical experimental results.

RESULTS

Notations

We use the following notations in the rest of the paper. For any natural number n, we define $[n] := \{1,\ldots,n\}$. For a matrix $Z \in \mathbb{R}^{n\times m}$, its i-th row and j-th column are denoted as $Z_{i:}$ and $Z_{:j}$, respectively, for $i \in [n]$ and $j \in [m]$. The L_1 norm, the L_2 norm of a vector $\boldsymbol{v} \in \mathbb{R}^n$ are defined as $\|\boldsymbol{v}\|_1 := \sum_{i\in[n]}|v_i|$ and $\|\boldsymbol{v}\|_2 := \sqrt{\sum_{i\in[n]}|v_i|^2}$, respectively. In addition, the symbols $\mathbf{0}$ and $\mathbf{1}$ denote vectors of suitable dimensionality, where all of their elements are equal to 0 and 1, respectively.

Problem setup: Predictive pattern mining

Let $\mathcal{S}$ be the input space for structure data, e.g., $\mathcal{S}$ is a collection of sets, graphs, and sequences. We assume that a certain partial order $\sqsubset$ on $\mathcal{S}$ is defined, which represents an inclusion relationship. For example, in the case of subset mining, we can use well known subset operator $\subset$ as $\sqsubset$. Let $\mathcal{Y}$ be the space of response (i.e., output space). We consider $\mathcal{Y} \subseteq \mathbb{R}$ for regression problems and $\mathcal{Y} = \{-1, +1\}$ for binary classification problems.

We denote the training dataset with n instances as $\mathcal{D} = \{(S_i, y_i)\}_{i\in[n]}$, where $S_i \in \mathcal{S}$ and $y_i \in \mathcal{Y}$ are the pair of structure input and response for the i-th training instance. Let $\mathcal{P}$ be the set of all sub-structures (patterns) contained in $\{S_i\}_{i\in[n]}$ and $p_1, p_2, \ldots, p_d \in \mathcal{P}$ be the elements of $\mathcal{P}$. Here, d is the total number of patterns, which increases exponentially with the complexity of structure inputs. In predictive pattern mining, we consider a generalized linear model in the form of

$$g(y_i) = \beta_0 + \beta_1 x_{i1} + \beta_2 x_{i2} + \cdots + \beta_d x_{id}, i \in [n], \quad \text{(Equation 1)}$$

where x_{ij} equals 1 if the i-th input structure has the j-th pattern and 0 otherwise, β_0 and $\beta_j \in \mathbb{R}, j \in [d]$ are the model coefficient, and g is the link function for generalized linear model. With this notation, the training set for the predictive pattern mining is also represented as $(X, \boldsymbol{y})$, where $X \in \{0,1\}^{n\times(d+1)}$ is the matrix with the (i,j)-th element x_{ij} for $j \in [n]$ and 0-th column as the intercept, i.e., $x_{i0} = 1$, and $\boldsymbol{y} \in \mathbb{R}^n$ is the vector with y_i being the i-th response. Furthermore, we define a vector of coefficients as $\boldsymbol{\beta} = [\beta_0, \beta_1, \ldots, \beta_d] \in \mathbb{R}^{d+1}$. Then, the linear model in Equation 1 is simply written as $g(\boldsymbol{y}) = X\boldsymbol{\beta}$, where g is assumed to be applied to $\boldsymbol{y}$ in an element-wise manner.

The goal of predictive pattern mining is to find the vector of coefficients parameters $\boldsymbol{\beta} \in \mathbb{R}^{d+1}$ that minimizes the following class of loss function:

$$\begin{aligned} \boldsymbol{\beta} &:= \underset{\boldsymbol{\beta}\in\mathbb{R}^{d+1}}{\text{argmin}}\, P(\boldsymbol{\beta}), \\ P(\boldsymbol{\beta}) &:= L_{\boldsymbol{y}}(X\boldsymbol{\beta}) + \Omega_{(\lambda,\kappa)}(\boldsymbol{\beta}), \end{aligned} \quad \text{(Equation 2)}$$

where $L_{\boldsymbol{y}} : \mathbb{R}^n \to \mathbb{R}$ is a convex loss function with Lipschitz continuous gradient, and $\Omega_{(\lambda,\kappa)}$ is a convex regularization function. In

this paper, we focus on the following regularization function as $\Omega_{(\lambda,\kappa)}$, which is called elastic net regularization[34]:

$$\Omega_{(\lambda,\kappa)}(\boldsymbol{\beta}) = \lambda \sum_{j \in [d]} \left(|\beta_j| + \frac{\kappa}{2}\beta_j^2 \right), \quad \text{(Equation 3)}$$

where $\lambda > 0$ and $\kappa \geq 0$ are the hyperparameters for tuning the strength of regularization. Note that we assume that the intercept β_0 of $\boldsymbol{\beta}$ is not regularized. When using this regularization function, a sparse solution is obtained, meaning that many coefficients β_j^* shrink to zero in the optimal solution.

The dual problem of Equation 2, introduced by Fenchel's duality theorem (see Corollary 31.2.1 of Rockafellar,[35] for example), is written as

$$\begin{aligned} \boldsymbol{\alpha}^* &:= \max_{\boldsymbol{\alpha} \in \mathbb{R}^n} D(\boldsymbol{\alpha}), \\ D(\boldsymbol{\alpha}) &:= -L_{\boldsymbol{y}}^*(-\boldsymbol{\alpha}) - \Omega_{(\lambda,\kappa)}^*(X^\top \boldsymbol{\alpha}), \end{aligned} \quad \text{(Equation 4)}$$

where f^* indicates the convex conjugate of a convex function f, which is defined as follows.

Definition 1 (convex conjugate): Let $f : \mathbb{R}^n \to \mathbb{R}$ be a convex function, the convex conjugate $f^* : \mathbb{R}^n \to \mathbb{R}$ is defined as

$$f^*(\boldsymbol{v}) = \sup_{\boldsymbol{u} \in \mathbb{R}^n} \{\boldsymbol{u}^\top \boldsymbol{v} - f(\boldsymbol{u})\}.$$

In this paper, we efficiently solve the predictive pattern mining problem by effectively combining the primal and the dual problems.

Sparse learning and safe screening

A class of methods for obtaining sparse solutions by using a sparsity-inducing regularization term such as Equation 3 is called sparse learning. In sparse learning, the set of features whose optimal solution is non-zero is called the active set, and is denoted by

$$\mathcal{A}^* := \left\{ j \in [d] \,\middle|\, \beta_j^* \neq 0 \right\}.$$

One characteristic of sparse learning is that the optimal solution for a dataset containing the features in the active set gives the same optimal solution obtained for a dataset containing all features. Concretely, let us consider a superset $\mathcal{A} \supseteq \mathcal{A}^*$ of the active set and a dataset $(X_{\mathcal{A} \cup \{0\}}, \boldsymbol{y})$ containing only the features belonging to $\mathcal{A}$. Then, the optimal solution for this dataset

$$\boldsymbol{\beta}^*(\mathcal{A}) := \underset{\boldsymbol{\beta} \in \mathbb{R}^{|\mathcal{A}|+1}}{\operatorname{argmin}} L_{\boldsymbol{y}}\left(X_{\mathcal{A} \cup \{0\}} \boldsymbol{\beta}\right) + \Omega_{(\lambda,\kappa)}(\boldsymbol{\beta})$$

has a property that

$$\beta_j^*(\mathcal{A}) = \beta_j^*, \forall j \in \mathcal{A} \cup \{0\}.$$

This property implies that, if we can obtain a superset that contains the active set, it is sufficient to solve the optimization problem for a smaller dataset with smaller number of features.

In general, an active set cannot be obtained until the optimization problem is solved. However, by using an approach called safe screening, there is a case where it is possible to identify features that cannot be active in the optimal solution, i.e., features for which $\beta_j^* = 0, j \in [d]$, before solving the optimization problem.

Specifically, by using theorems 23.5 and 31.3 of,[35] it can be shown that the following relationship exists between the optimal solutions of the primal problem Equation 2 and the dual problem Equation 4, expressed as

$$X\boldsymbol{\beta}^* \in \partial L_{\boldsymbol{y}}^*(-\boldsymbol{\alpha}^*) \Leftrightarrow -\boldsymbol{\alpha}^* \in \partial L_{\boldsymbol{y}}(X\boldsymbol{\beta}^*), \quad \text{(Equation 5)}$$

$$X^\top \boldsymbol{\alpha}^* \in \partial \Omega_{(\lambda,\kappa)}(\boldsymbol{\beta}^*) \Leftrightarrow \boldsymbol{\beta}^* \in \partial \Omega_{(\lambda,\kappa)}^*(X^\top \boldsymbol{\alpha}^*), \quad \text{(Equation 6)}$$

where ∂f is the subgradient of f. In case of elastic net regularization, Ω^* is written as follows

$$\Omega^*(X^\top \boldsymbol{\alpha}) = \sum_{j \in [d] \cup \{0\}} \omega_j^*\left(X_{:j}^\top \boldsymbol{\alpha}\right),$$

$$\omega_0^*(v) = \begin{cases} 0 & (v = 0) \\ +\infty & (v \neq 0) \end{cases},$$

$$\omega_j^*(v) = \begin{cases} 0 & (|v| \leq \lambda) \\ (|v| - \lambda)^2 / (2\kappa\lambda) & (\kappa > 0 \text{ and } |v| > \lambda) \\ +\infty & (\kappa = 0 \text{ and } |v| > \lambda) \end{cases}, \forall j \in [d],$$

and noting that subgradients are defined only on their feasible regions, i.e., $v \in \mathbb{R}$ such that $\omega_j^*(v) \neq +\infty$, $\partial\omega_j^*$ is also written down as

$$\partial\omega_0^*(0) = \mathbb{R},$$

$$\partial\omega_j^*(v) = \begin{cases} \{0\} & (|v| < \lambda) \\ \{\mathrm{sign}(v)(|v| - \lambda)/(\kappa\lambda)\} & (\kappa > 0 \text{ and } |v| \geq \lambda) \\ [0, +\infty) & (\kappa = 0 \text{ and } v = \lambda) \\ (-\infty, 0] & (\kappa = 0 \text{ and } v = -\lambda) \end{cases}, \forall j \in [d]. \quad \text{(Equation 7)}$$

From the right-hand side of Equation 6 and Equation 7, we can obtain the following simple relationship:

$$\left|X_{:j}{}^\top \boldsymbol{\alpha}^*\right| < \lambda \Rightarrow \beta_j^* = 0, \forall j \in [d]. \quad \text{(Equation 8)}$$

The basic idea of safe screening is to compute an upper bound on $\left|X_{:j}{}^\top \boldsymbol{\alpha}^*\right|$ in Equation 8. If the upper bound is smaller than λ, the condition in Equation 8 is satisfied, meaning that the optimal solution of the corresponding primal problem is $\beta_j^* = 0$ and this feature can be removed beforehand. Since safe screening was first proposed, serveral improvements have been made,[36–41] and its range of applications has been expanded.[42–48] Especially, it is known that GAP safe screening[38,39] has good performance of screening, and there are several studies that have utilized GAP safe screening in recent years. Extensions to specialized regularizers have been proposed.[49,50] There is a method alleviates the assumption that the dual problem is strongly convex.[51] Furthermore, there are studies on GAP safe screening for optimization algorithm based on stochastic gradient descent.[52,53]

In this paper, we also employ GAP safe screening. The basic idea of GAP safe screening is to use an arbitrary primal feasible solution $\tilde{\boldsymbol{\beta}} \in \mathbb{R}^{d+1}$ and an arbitrary dual feasible solution $\tilde{\boldsymbol{\alpha}} \in \mathbb{R}^n$ to compute an upper bound on $\left|X_{:j}{}^\top \boldsymbol{\alpha}^*\right|$. The following lemma states that, given a pair of primal and dual feasible solutions $(\tilde{\boldsymbol{\beta}},$

CellPress
OPEN ACCESS

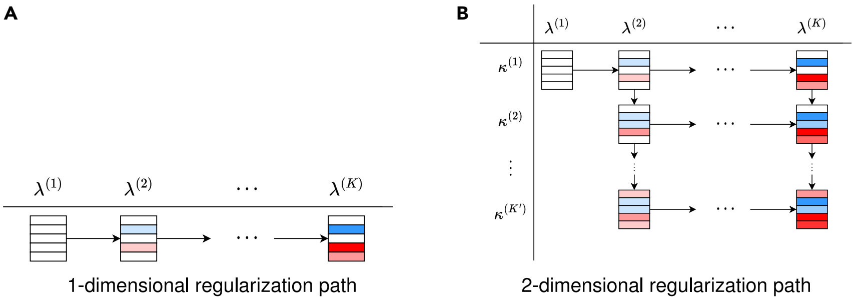

Figure 4. Regularization path for the one-dimensional and two-dimensional regularization parameter

Schematic illustration of (A) regularization path for the L_1-norm regularization parameter λ and (B) two-dimensional regularization path for the $L1$-norm regularization parameter λ and the relative regularization parameter κ for the $L2$-norm in the elastic net. The rectangles in each cell represent the β_j^* at the corresponding regularization parameter. The color of each rectangle indicates the value of β_j^* where red/blue shows its signs while the thickness of the color indicates the absolute value (illustrating the increase of active (non-zero) coefficients and their absolute values as λ decreases). Note that, in (B), when $\lambda = \lambda^{(1)}$ and $\boldsymbol{\beta}^* = 0$, it is not necessary to change κ.

$\tilde{\boldsymbol{\alpha}}$), it is possible to determine the range of the dual optimal solution $\boldsymbol{\alpha}^*$.

Lemma 2 (GAP safe screening rule): Suppose that ∇L is Lipschitz continuous with constant $\gamma > 0$. For any pair of feasible solution $(\tilde{\boldsymbol{\beta}}, \tilde{\boldsymbol{\alpha}})$ and $j \in [d]$, let us define, what is called, safe screening score as follows:

$$u_j(\tilde{\boldsymbol{\beta}}, \tilde{\boldsymbol{\alpha}}) := \left|X_{:j}{}^\top \tilde{\boldsymbol{\alpha}}\right| + r(\tilde{\boldsymbol{\beta}}, \tilde{\boldsymbol{\alpha}}) \|X_{:j} - \Pi_{\mathbf{1}}(X_{:j})\|_2. \qquad \text{(Equation 9)}$$

Then,

$$u_j(\tilde{\boldsymbol{\beta}}, \tilde{\boldsymbol{\alpha}}) < \lambda \Rightarrow \beta_j^* = 0, \qquad \text{(Equation 10)}$$

where

$$r(\tilde{\boldsymbol{\beta}}, \tilde{\boldsymbol{\alpha}}) := \sqrt{2\gamma(P(\tilde{\boldsymbol{\beta}}) - D(\tilde{\boldsymbol{\alpha}}))},$$

$$\Pi_{\boldsymbol{u}}(\boldsymbol{v}) := \frac{\boldsymbol{u}^\top \boldsymbol{v}}{\boldsymbol{u}^\top \boldsymbol{u}} \boldsymbol{u}, \boldsymbol{u}, \boldsymbol{v} \in \mathbb{R}^n.$$

($\Pi_{\boldsymbol{u}}(\boldsymbol{v})$ is known as the projection of $\boldsymbol{v}$ onto $\boldsymbol{u}$.)

The proof of lemma 2 is presented in Note S1. This lemma indicates that, given a pair of primal and dual feasible solutions $(\tilde{\boldsymbol{\beta}}, \tilde{\boldsymbol{\alpha}})$, we can first compute the upper bound $u_j(\tilde{\boldsymbol{\beta}}, \tilde{\boldsymbol{\alpha}})$ for each $j \in [d]$, and remove the feature if $u_j(\tilde{\boldsymbol{\beta}}, \tilde{\boldsymbol{\alpha}}) < \lambda$.

Note that this lemma requires both the primal feasible solution $\tilde{\boldsymbol{\beta}}$ and the dual feasible solution $\tilde{\boldsymbol{\alpha}}$; however, most algorithms that solve problem Equation 2 update only $\tilde{\boldsymbol{\beta}}$ and hence $\tilde{\boldsymbol{\alpha}}$ is not available. To obtain $\tilde{\boldsymbol{\alpha}}$, we assume that the relationship on the right-hand side of Equation 5 holds even if $\tilde{\boldsymbol{\beta}}$ is not optimal

$$-\overline{\boldsymbol{\alpha}} = \nabla L(X\tilde{\boldsymbol{\beta}}),$$

where we assume that L is differentiable in this paper, and hence ∂L is equivalent to ∇L. Since there is no guarantee that $\overline{\boldsymbol{\alpha}}$ calculated in this way is feasible, a process to move it into the feasible region is required (referred to as dual scaling). The detailed computation depends on L and Ω. For instance, if L is the squared loss, i.e., $L(\boldsymbol{v}) = \|\boldsymbol{v} - \boldsymbol{y}\|_2^2/(2n)$ and $\kappa > 0$ for elastic net, the feasible region is determined solely by the feasiblity condition of ω_0^*, i.e., $\mathbf{1}^\top \boldsymbol{\alpha} = 0$. Thus, it is enough to compute $\tilde{\boldsymbol{\alpha}}$ as $\tilde{\boldsymbol{\alpha}} = \overline{\boldsymbol{\alpha}} - \overline{\boldsymbol{\alpha}}^\top \mathbf{1}$. If $\kappa = 0$, there is a feasible condition on ω_j^*, i.e., $\left|X_{:j}^\top \boldsymbol{\alpha}\right| \le \lambda$ for all $j \in [d]$ or $\max_{j \in [d]} \left|X_{:j}^\top \boldsymbol{\alpha}\right|$. Taking feasiblity of ω_0^* into account, we need to compute $\tilde{\boldsymbol{\alpha}}$ such that $\tilde{\boldsymbol{\alpha}} = \mu(\overline{\boldsymbol{\alpha}} - \overline{\boldsymbol{\alpha}}^\top \mathbf{1})$, where

$$\mu = \min\left\{\max\left\{\frac{\overline{\overline{\boldsymbol{\alpha}}}^\top \boldsymbol{y}}{\|\overline{\overline{\boldsymbol{\alpha}}}\|_2^2}, -\frac{\lambda}{\max_{j \in [d]} \left|X_{:j}^\top \overline{\overline{\boldsymbol{\alpha}}}\right|}\right\}, \frac{\lambda}{\max_{j \in [d]} \left|X_{:j}^\top \overline{\overline{\boldsymbol{\alpha}}}\right|}\right\},$$

$$\overline{\overline{\boldsymbol{\alpha}}} = \overline{\boldsymbol{\alpha}} - \overline{\boldsymbol{\alpha}}^\top \mathbf{1},$$

which is calculated to minimize duality gap $P(\boldsymbol{\beta}) - D(\boldsymbol{\alpha})$ within the feasible region for faster convergence. Another example considered in this paper is the squared hinge loss, i.e., $L(\boldsymbol{v}) = \sum_{i=1}^n \max\{1 - y_i v_i, 0\}^2/(2n)$. In this case, there is a feasiblity condition on $L_{\boldsymbol{y}}^*$ such that $y_i \alpha_i < 0$. To satisfy the feasiblity conditions of both $L_{\boldsymbol{y}}^*$ and Ω^*, we apply different scaling for positive instances and negative instances, i.e., $\tilde{\alpha}_i = \mu_+ \overline{\alpha}_i$ for $y_i = +1$ and $\tilde{\alpha}_i = \mu_- \overline{\alpha}_i$ for $y_i = -1$. We compute the ratio between the scaling factors as $\mu_+/\mu_- = -\sum_{i:y_i=-1} \overline{\alpha}_i / \sum_{i:y_i=+1} \overline{\alpha}_i$. Then we compute μ_- in the manner similar to the squared loss case,

$$\mu_- = \min\left\{\max\left\{\frac{\overline{\overline{\boldsymbol{\alpha}}}^\top \boldsymbol{y}}{\|\overline{\overline{\boldsymbol{\alpha}}}\|_2^2}, 0\right\}, \frac{\lambda}{\max_{j \in [d]} \left|X_{:j}^\top \overline{\overline{\boldsymbol{\alpha}}}\right|}\right\},$$

$$\overline{\overline{\boldsymbol{\alpha}}} = \begin{cases} \mu_+/\mu_- \overline{\alpha}_i & (y_i = +1) \\ \overline{\alpha}_i & (y_i = -1) \end{cases}, \tilde{\boldsymbol{\alpha}} = \mu_- \overline{\overline{\boldsymbol{\alpha}}}.$$

If the ratio μ_+/μ_- is unavailable due to $\sum_{i:y_i=+1} \overline{\alpha}_i = 0$, then we compute the ratio μ_-/μ_+ and determine μ_+ instead.

Our basic idea is to apply this GAP safe screening to predictive pattern mining. However, since the number of all possible features d is exponentially increasing, it is impossible to compute the upper bound $u_j(\tilde{\boldsymbol{\beta}}, \tilde{\boldsymbol{\alpha}})$ for each pattern. To address this challenge, in the next section, we extend the safe screening rule so that it can identify a group of removable patterns at once.

SPP

The basic idea of SPP is to represent the relationship among patterns in a tree (Figure 2) and identify a group of patterns for which the optimal coefficients satisfy $\beta_j^* = 0$ by pruning the tree. To obtain the pruning rule, we exploit the monotonicity of patterns, i.e., the occurrence of patterns decreases monotonically as pattern grows in the tree. We describe this property in the following lemma more specifically.

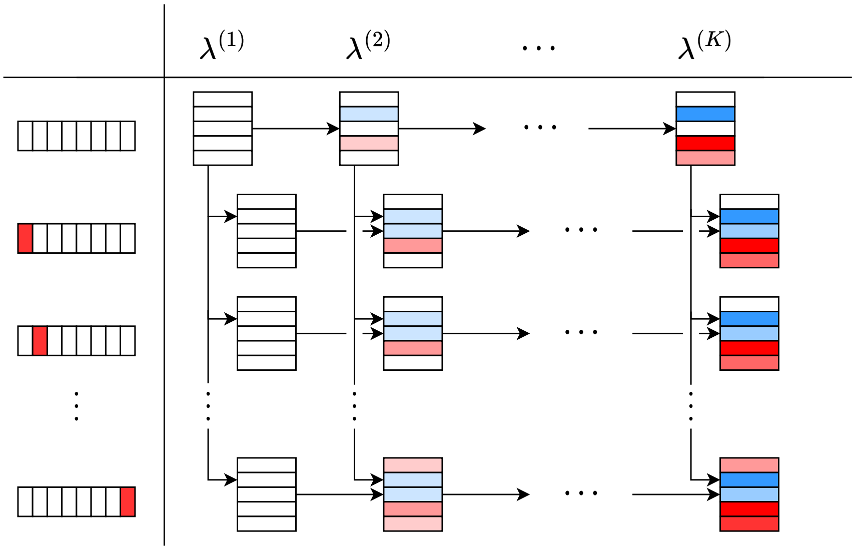

Figure 5. A schematic illustration of how to use feasible solutions in hyperparameter selection based on CV

The left rectangle represents the training data, with white corresponding with the training data and red with the validation data. In using multiple solutions for CV setting, we not only use the optimal solution at the previous regularization parameter, but also use the optimal solution trained with the entire data.

Lemma 3 (monotonicity of patterns): Let $p_j, p_k \in \mathcal{S}$ be patterns in $\{S_i\}_{i \in [n]}$ such that $p_k \sqsubset p_j$.

Then, for any $i \in [n]$,

$$x_{ik} = 1 \Rightarrow x_{ij} = 1.$$

This is obvious because, if an input instance S_i has p_k (i.e., $p_k \sqsubset S_i$), then $p_k \sqsubset p_j \sqsubset S_i$ also holds. Using this lemma, we derive the following theorem.

Theorem 4 (SPP rule): For any pair of feasible solution $(\tilde{\boldsymbol{\beta}}, \tilde{\boldsymbol{\alpha}})$ and $j \in [d]$, let us define, what we call the SPP score, as follows:

$$v_j(\tilde{\boldsymbol{\beta}}, \tilde{\boldsymbol{\alpha}}) := \max\left\{ \sum_{i:\tilde{\alpha}_i > 0} x_{ij}\tilde{\alpha}_i, -\sum_{i:\tilde{\alpha}_i < 0} x_{ij}\tilde{\alpha}_i \right\} + r(\tilde{\boldsymbol{\beta}}, \tilde{\boldsymbol{\alpha}}) \| X_{:j} \|_2. \quad \text{(Equation 11)}$$

Then,

$$v_j\left(\tilde{\boldsymbol{\beta}}, \tilde{\boldsymbol{\alpha}}\right) < \lambda \Rightarrow \beta_k^* = 0 \ \forall k \in [d] \ s.t. \ \mathrm{p_k} \sqsubset \mathrm{p_j}.$$

The proof is presented in Note S2. Using theorem 4, it is possible to screen a group of patterns at once during the process of searching in the tree that represents the relationships between patterns. Specifically, when searching for the screen-able patterns in the tree from the root node to descent nodes, if the SPP score of a pattern p_j corresponding with a certain node of the tree satisfies $v_j(\tilde{\boldsymbol{\beta}}, \tilde{\boldsymbol{\alpha}}) < \lambda$, all patterns p_k corresponding with its descendant nodes satisfies $p_k \sqsubset p_j$, so they can be screened out as unnecessary patterns. Algorithm 1 in Note S4 shows the pseudo-code of the SPP.

To apply the SPP to actual predictive pattern mining, a pair of feasible solutions $(\tilde{\boldsymbol{\beta}}, \tilde{\boldsymbol{\alpha}})$ for the primal and dual problems is necessary. Although the SPP rules hold for any feasible solutions $(\tilde{\boldsymbol{\beta}}, \tilde{\boldsymbol{\alpha}})$, the tightness of the bound depends on the choice of feasible solutions. Specifically, because the tightness of the SPP bound is determined by the duality gap $P(\tilde{\boldsymbol{\beta}}) - D(\tilde{\boldsymbol{\alpha}})$ of the feasible solution, the closer the pair of feasible solutions $(\tilde{\boldsymbol{\beta}}, \tilde{\boldsymbol{\alpha}})$ is to the (unknown) pair of optimal solutions $(\boldsymbol{\beta}^*, \boldsymbol{\alpha}^*)$, the tighter the SPP bound will be. Therefore, when applying the SPP to actual predictive pattern mining, it is important to obtain feasible solutions that are sufficiently close to the optimal solutions.

SPP for model selection

As mentioned in the previous section, to screen out features using the SPP, a feasible pair of solutions $(\tilde{\boldsymbol{\beta}}, \tilde{\boldsymbol{\alpha}})$ for the primal and dual problems, respectively, that are sufficiently close to the optimal solutions $(\boldsymbol{\beta}^*, \boldsymbol{\alpha}^*)$ is necessary. In practical data analysis, it is often necessary to learn multiple models rather than just obtaining a single predictive pattern mining model, e.g., in selecting hyperparameters λ, κ, or evaluating the generalization performance through cross-validation (CV). In this section, we discuss how to apply the SPP for a series of model fittings in model selection. Our main idea is to use the optimal solutions of models fitted in slightly different problem settings (e.g., with similar hyperparameter values or with only a part of the data being different in CV) as reference feasible solutions for the SPP. Furthermore, we propose an extension of the SPP that enables more effective utilization of multiple reference feasible solutions in practical model fitting scenarios where multiple reference feasible solutions are naturally available.

First, in "SPP with multiple pairs of feasible solutions," we describe an extension of safe screening using two different reference feasible solutions. Next, in "Multiple dynamic screening with SPP," we introduce an approach called dynamic screening in which the solutions obtained during learning process are used as reference feasible solutions for the SPP. Furthermore, in "SPP with multiple hyperparameter selection," we consider how to apply the SPP in model selection process in which two hyperparameters λ and κ are optimized. Finally, in "SPP with hyperparameter selection by CV," we discuss how to apply the SPP when selecting hyperparameters using CV.

SPP with multiple pairs of feasible solutions

Let us consider the case where two feasible solutions $R_1 := (\tilde{\boldsymbol{\beta}}^{(1)}, \tilde{\boldsymbol{\alpha}}^{(1)})$, $R_2 := (\tilde{\boldsymbol{\beta}}^{(2)}, \tilde{\boldsymbol{\alpha}}^{(2)})$ are available. From lemma 3, the optimal solution $\boldsymbol{\alpha}^*$ must be contained in both of the two hyperspheres $B_1 := B(R_1)$ and $B_2 := B(R_2)$. Therefore, it is possible to further narrow down the range of $\boldsymbol{\alpha}^*$ to $B_1 \cap B_2$, and consider a tighter upper bound

$$\max_{\boldsymbol{\alpha} \in B_1 \cap B_2 \cap H} \left| X_{:j}{}^\top \boldsymbol{\alpha} \right| \leq \min\left\{ \max_{\boldsymbol{\alpha} \in B_1 \cap H} \left| X_{:j}{}^\top \boldsymbol{\alpha} \right|, \max_{\boldsymbol{\alpha} \in B_2 \cap H} \left| X_{:j}{}^\top \boldsymbol{\alpha} \right| \right\},$$

where $H = \{\boldsymbol{\alpha} \in \mathbb{R}^n | \mathbf{1}^\top \boldsymbol{\alpha} = 0\}$ is introduced by the feasiblity of ω_0^*. By using this tighter upper bound in the safe screening, we

expect that more inactive patterns can be screened out. For a pattern j, if $\max_{\alpha \in B_1 \cap B_2 \cap H} |X_{:j}{}^\top \boldsymbol{\alpha}| < \lambda \le \min\{\max_{\alpha \in B_1 \cap H} |X_{:j}{}^\top \boldsymbol{\alpha}|, \max_{\alpha \in B_2 \cap H} |X_{:j}{}^\top \boldsymbol{\alpha}|\}$, then it is not possible to remove the pattern j using either B_1 or B_2 alone, but it becomes possible to remove it by using the intersection of B_1 and B_2. The following theorem indicates that $\max_{\alpha \in B_1 \cap B_2 \cap H} |X_{:j}{}^\top \boldsymbol{\alpha}|$ can be expressed in a closed form and can be computed in $\mathcal{O}(n)$ time.

Theorem 5 (multiple safe screening rule): For any pair of primal-dual feasible solutions $R_1 = (\tilde{\boldsymbol{\beta}}^{(1)}, \tilde{\boldsymbol{\alpha}}^{(1)}), R_2 = (\tilde{\boldsymbol{\beta}}^{(2)}, \tilde{\boldsymbol{\alpha}}^{(2)})$, and for any $j \in [d]$, it holds that

$$u'_j(R_1, R_2) := \max_{\alpha \in B_1 \cap B_2 \cap H} |X_{:j}{}^\top \boldsymbol{\alpha}| = \max\left\{u_j^+, u_j^-\right\} < \lambda \Rightarrow \beta_j^* = 0,$$

where

$$u_j^+ := \begin{cases} X_{:j}{}^\top \tilde{\boldsymbol{\alpha}}^{(1)} + r(R_1)\|X_{:j} - \Pi_{\mathbf{1}}(X_{:j})\|_2, & X_{:j} \in C_1, \\ X_{:j}{}^\top \tilde{\boldsymbol{\alpha}}^{(2)} + r(R_2)\|X_{:j} - \Pi_{\mathbf{1}}(X_{:j})\|_2, & X_{:j} \in C_2, \\ X_{:j}{}^\top \tilde{\boldsymbol{\alpha}}' + r'\|X_{:j} - \Pi_{\mathbf{1}}(X_{:j}) - \Pi_{\boldsymbol{\delta}}(X_{:j})\|_2, & \text{otherwise}, \end{cases}$$

$$u_j^- := \begin{cases} -X_{:j}{}^\top \tilde{\boldsymbol{\alpha}}^{(1)} + r(R_1)\|X_{:j} - \Pi_{\mathbf{1}}(X_{:j})\|_2, & -X_{:j} \in C_1, \\ -X_{:j}{}^\top \tilde{\boldsymbol{\alpha}}^{(2)} + r(R_2)\|X_{:j} - \Pi_{\mathbf{1}}(X_{:j})\|_2, & -X_{:j} \in C_2, \\ -X_{:j}{}^\top \tilde{\boldsymbol{\alpha}}' + r'\|X_{:j} - \Pi_{\mathbf{1}}(X_{:j}) - \Pi_{\boldsymbol{\delta}}(X_{:j})\|_2, & \text{otherwise}, \end{cases}$$

and

$$\boldsymbol{\delta} := \tilde{\boldsymbol{\alpha}}^{(1)} - \tilde{\boldsymbol{\alpha}}^{(2)},$$

$$\tilde{\boldsymbol{\alpha}}' := t\tilde{\boldsymbol{\alpha}}^{(1)} + (1 - t)\tilde{\boldsymbol{\alpha}}^{(2)},$$

$$r' := \sqrt{r(R_2)^2 - t^2\|\boldsymbol{\delta}\|_2^2},$$

$$t := \frac{1}{2}\left(1 + \frac{r(R_2)^2 - r(R_1)^2}{\|\boldsymbol{\delta}\|_2^2}\right),$$

$$C_1 := \left\{\boldsymbol{a} \in \mathbb{R}^n \middle| \frac{\boldsymbol{a}^\top \boldsymbol{\delta}}{\|\boldsymbol{a} - \Pi_{\mathbf{1}}(\boldsymbol{a})\|_2} \le \frac{r(R_2)^2 - r(R_1)^2 - \|\boldsymbol{\delta}\|_2^2}{2r(R_1)}\right\},$$

$$C_2 := \left\{\boldsymbol{a} \in \mathbb{R}^n \middle| \frac{\boldsymbol{a}^\top \boldsymbol{\delta}}{\|\boldsymbol{a} - \Pi_{\mathbf{1}}(\boldsymbol{a})\|_2} \ge \frac{r(R_2)^2 - r(R_1)^2 + \|\boldsymbol{\delta}\|_2^2}{2r(R_2)}\right\}.$$

The proof of this theorem is presented in Note S3.

Instead of the safe screening rule in theorem 5, we may consider the safe pruning rule for two reference solutions. However, unlike the safe screening case above, we conjecture that the pruning conditions cannot be written in a closed form. So, for the safe pruning with two reference solutions, we just apply two pruning rules derived by each of the solutions:

$$v'_j(R_1, R_2) := \min\{v_j(R_1), v_j(R_2)\} < \lambda \Rightarrow \forall p_j \sqsubset p_k, \beta_k^* = 0.$$

In addition, if we have three or more reference solutions, we expect that we can screen out more features. However, we conjecture that the screening conditions become very complicated as the number of reference solutions increases. □

Multiple dynamic screening with SPP

In this section, we describe the extension of multiple safe screening to dynamic screening.[41] Dynamic screening is a method of performing safe screening using feasible solutions obtained during optimization. Because the performance of safe screening depends on how close the feasible solution is to the optimal solution, more patterns tend to be removed with updated solutions. This means that, if the update to the solution is not substantial enough, the performance of safe screening may not differ significantly between before and after the updates. In the case where multiple feasible solutions are available, all of the solutions must be sufficiently updated. However, updating multiple solutions needs additional computational costs, so it is necessary to consider the trade-off between the cost of updating multiple solutions and the number of patterns that can be removed by safe screening.

To reduce the cost of updating multiple solutions, we restrict the number of multiple solutions updates to $M \in \mathbb{N}$. Specifically, we repeat updates and screening for multiple solutions up to M iterations, and then keep the single solution that has the smallest duality gap, which is an indicator of proximity to the optimal solution, while the others are discarded. Although there are no theoretical indicators to select M, we demonstrate that the optimizations with $M = 1$ show better results, and setting M larger than 1 does not have much of an effect in numerical experiments.

SPP with multiple hyperparameter selection

In this section, we describe a method for accelerating the computation of regularization paths for multiple hyperparameters using screening and pruning with multiple reference feasible solutions. Specifically, we consider the regularization paths for the two hyperparameters of the elastic net. When there are two hyperparameters, we can consider a two-dimensional regularization path as shown in Figure 4, where the sequence of regularization parameters for the L_1 norm is represented by $\lambda^{(1)}, \lambda^{(2)}, \ldots$, and the sequence of regularization parameters that adjust the relative strength of the L_2 norm is represented by $\kappa^{(1)}, \kappa^{(2)}, \ldots$. For a given set of hyperparameters $(\lambda^{(t)}, \kappa^{(t')})$, there are two feasible solutions that can be used as reference solutions for optimization, i.e., the optimal solution at $(\lambda^{(t-1)}, \kappa^{(t')})$ and the optimal solution at $(\lambda^{(t)}, \kappa^{(t'-1)})$.

For the hyperparameters λ and κ, we considered the following sequences of candidates: $\lambda^{(k)}, k \in [K]$ such that $\lambda^{(1)} > \lambda^{(2)} > \cdots > \lambda^{(K)}$ and $\kappa^{(k')}, k' \in K'$ such that $\kappa^{(1)} < \kappa^{(2)} < \cdots < \kappa^{(K')}$, respectively. In many cases, as κ increases for a fixed $\lambda^{(k)}$, the number of patterns for which $\beta_j^* \neq 0$ decreases. Therefore, by setting $\kappa^{(k')}$ in this way, the number of patterns for which $\beta_j^* \neq 0$ increases as k' becomes larger. Furthermore, we set $\lambda^{(1)}$ as the smallest λ value such that $\boldsymbol{\beta}^* = \mathbf{0}$ and set $\kappa^{(1)} = 0$.

In the case of having two regularization parameters, iterative optimization is performed in a manner analogous to the case of a single parameter. There are multiple possible options to optimize the regularization parameter sequence. In this paper, we adopt an option to optimize them in the order of $(\lambda^{(1)}, \kappa^{(1)})$, $(\lambda^{(1)}, \kappa^{(2)})$, ..., $(\lambda^{(1)}, \kappa^{(K')})$, $(\lambda^{(2)}, \kappa^{(1)})$, ..., $(\lambda^{(2)}, \kappa^{(K')})$, ..., $(\lambda^{(K)}, \kappa^{(K')})$.

Let $\mathcal{R}$ denote the set of feasible solutions. During optimization at $(\lambda^{(k)}, \kappa^{(k')})$, if $k > 1$, the optimal solution at $(\lambda^{(k-1)}, \kappa^{(k')})$ is appended to $\mathcal{R}$. On the other hand, if $k' > 1$, the optimal solution at $(\lambda^{(k)}, \kappa^{(k'-1)})$ is appended to $\mathcal{R}$. When $|\mathcal{R}| = 1$, we execute safe pruning and screening using a single feasible solution in conventional way. In contrast, when $|\mathcal{R}| = 2$, we execute pruning and screening using two feasible solutions. The detailed algorithm is described in Algorithms 2 and 3 in Note S4. The former is for one-dimensional settings and the latter is for two-dimensional

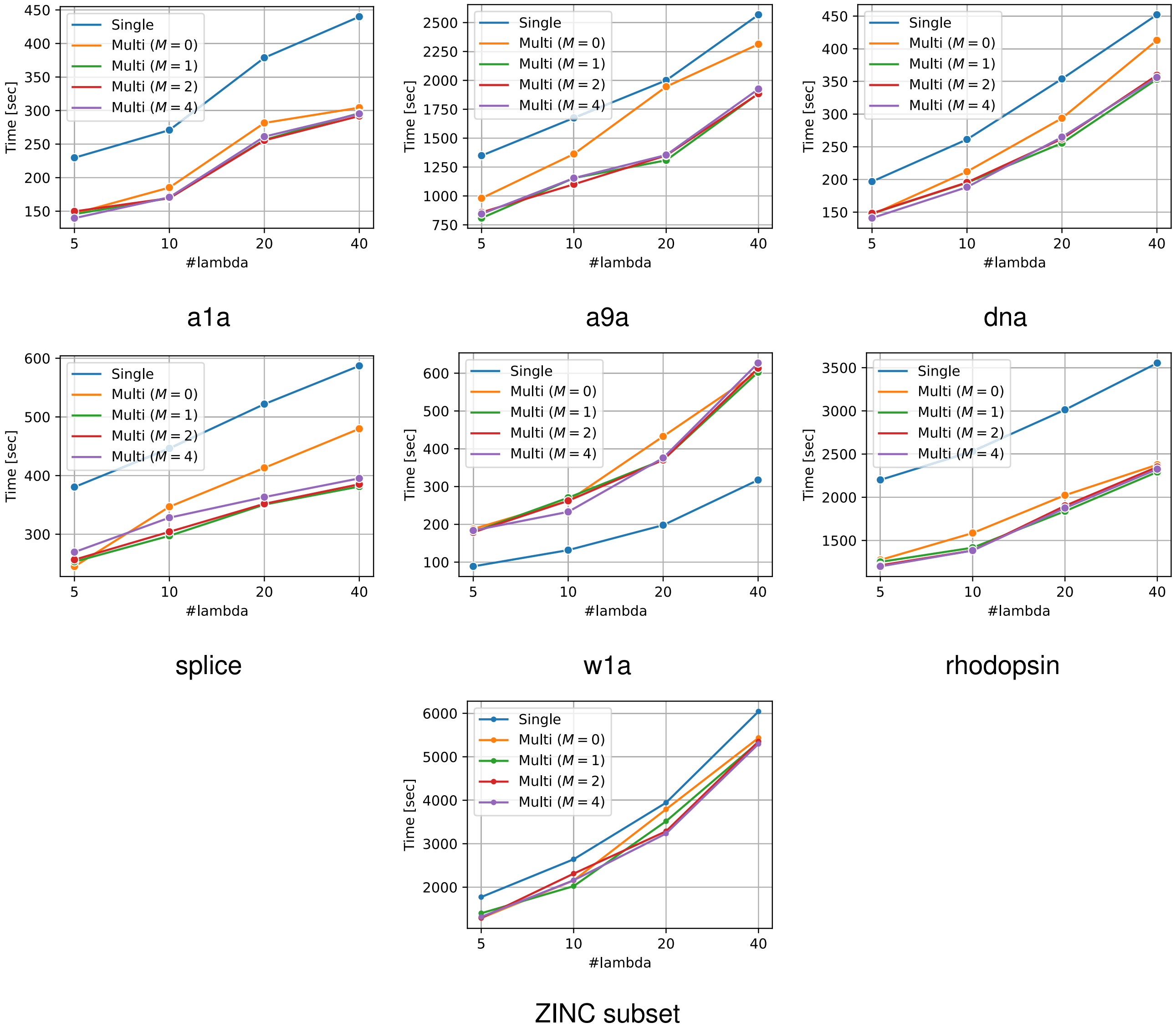

Figure 6. Computation time for the entire regularization path for each dataset

The horizontal axis shows how many partitions of λ were made. It can be confirmed that the use of multiple solutions is effective in most cases. In addition, the multi-dynamic screening also often leads to a reduction in computation time.

ones. In exploiting theorem 5 in the algorithm, we require two pairs of feasible solutions. It is obvious that the optimal solution at $(\lambda^{(k-1)}, \kappa^{(k')})$ and $(\lambda^{(k)}, \kappa^{(k'-1)})$ may not be feasible for optimization at $(\lambda^{(k)}, \kappa^{(k)})$. To address this issue, we utilize dual scaling as described in "Sparse learning and safe screening" for both solutions. Note that this approach can be easily extended to cases with three or more hyperparameters although it is not explicitly described in this paper.

SPP with hyperparameter selection by CV

CV is commonly used for determining hyperparameters. In CV, the following steps are taken to determine hyperparameters. First, the given dataset is divided into several groups. Then, one of the groups is used as the validation set, while the remaining groups are used for model training. Performance metrics such as prediction errors and classification accuracy are calculated for each hyperparameter(s) candidate using the validation data. This process is repeated by sequentially swapping the validation group, and the metrics are averaged for each hyperparameter(s) candidate. The hyperparameter candidate(s) with the highest average score is selected as the best hyperparameter(s). In such a CV process, a sequence of optimization problems with slightly different training set are solved one by one for each hyperparameter.

Our idea here is to use optimal solutions obtained at different steps of CV as another reference feasible solutions. Specifically, we use two reference feasible solutions obtained as the optimal solutions at different hyperparameters and at different CV steps and perform safe screening and pruning using these two reference feasible solutions as described in "SPP with multiple pairs of feasible solutions.. Let $\mathcal{I}^{(1)} = [n]$ denote the set of indices of the entire dataset, and consider a sequence of its subset,

CellPress
OPEN ACCESS

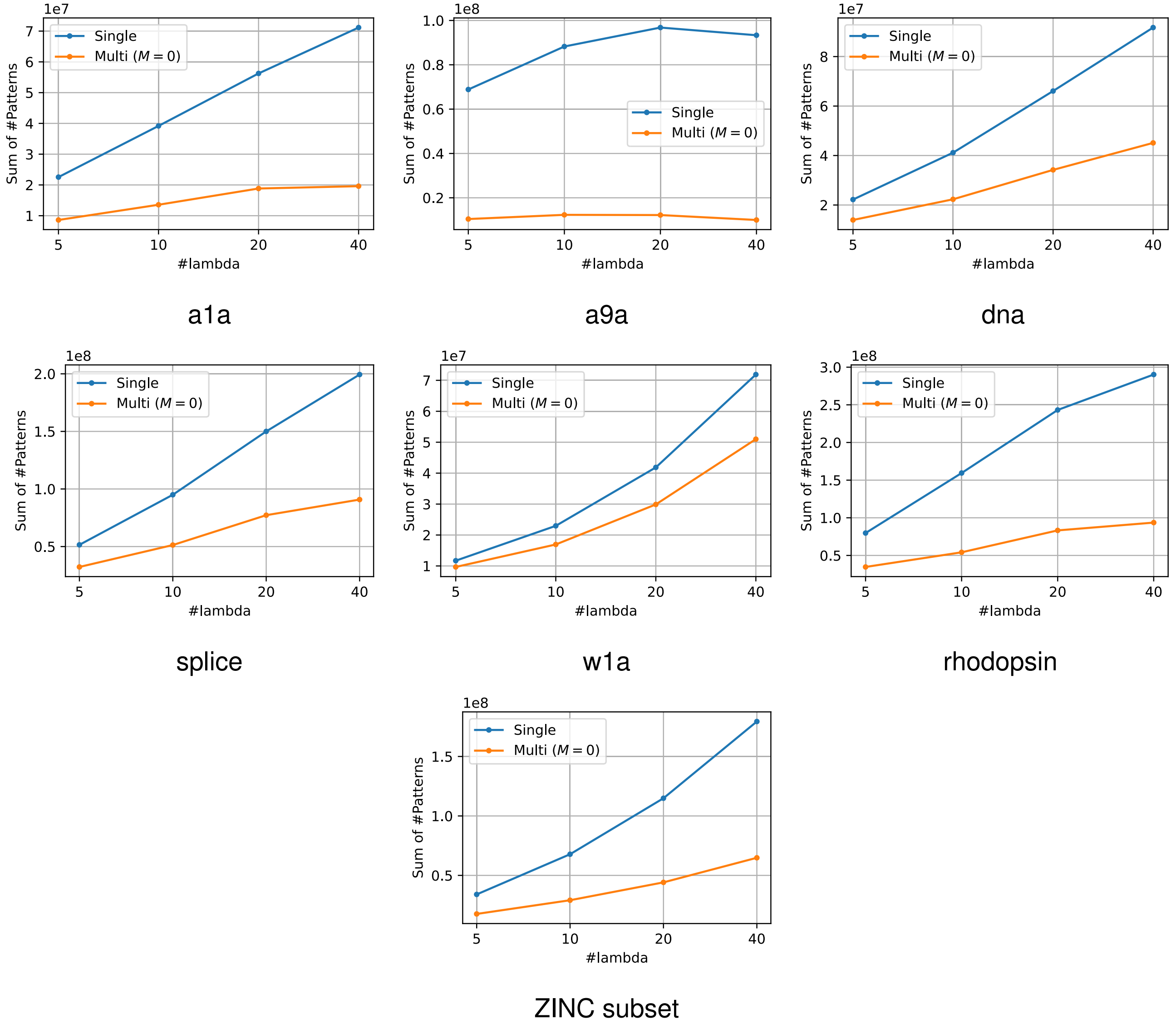

Figure 7. The number of patterns after SPP before optimization

These values represent the sums over the entire regularization path for each number of λs. Our multiple feasible solution approach significantly reduces the number of patterns in most datasets. Note that the number of patterns after pruning is irrelevant to M, hence we compared only the single reference case and multiple reference case with $M = 0$.

denoted by $\mathcal{I}^{(2)}, \mathcal{I}^{(3)}, \ldots, \mathcal{I}^{(K)} \subsetneq \mathcal{I}^{(1)}$. Figure 5 shows a schematic diagram of this procedure, and the details are described in Algorithm 4 in Note S4. Given a sequence of subscript sets $\{\mathcal{I}^{(k)}\}_{k \in [K]}$ and a sequence of hyperparameters $\{\lambda^{k'}\}_{k' \in [K']}$, we use the reference feasible solutions of the optimal solutions at $(\mathcal{I}^{(1)}, \lambda^{(k')})$ and $(\mathcal{I}^{(k)}, \lambda^{(k'-1)})$ during optimization of $(\mathcal{I}^{(k)}, \lambda^{(k')})$. Similar to the case of multiple hyperparameter selection, we apply dual scaling to both solutions to ensure their feasibility. Note that, in Algorithm 3, κ is fixed for simplicity, but it is possible to extend it to select both λ and κ.

Numerical experiments

In this section, we describe numerical experiments that verify the effectiveness of the proposed SPP method and its extension in model selection scenario.

SPP with multiple hyperparameter selection

We first conducted a comparison of computation times for two-dimensional regularization path calculations for the two regularization parameters, λ and κ, in the elastic net. We compared the performances of Single-SPP, which utilizes only a single reference feasible solution, and multi-SPP, which uses multiple reference feasible solutions. In the case of multi-SPP, experiments were performed for $M \in \{0, 1, 2, 4\}$, where M represents the number of times that multiple dynamic screening is executed (note that $M = 0$ signifies the use of multiple solutions solely for screening and pruning at the start of optimization, with dynamic screening performed using only a single solution thereafter). Regarding λ, we investigated cases where the number of partitions from $\lambda_{\max}$ to $0.01\lambda_{\max}$ was 5, 10, 20, and 40, where $\lambda_{\max}$ is the smallest λ that makes all patterns inactive

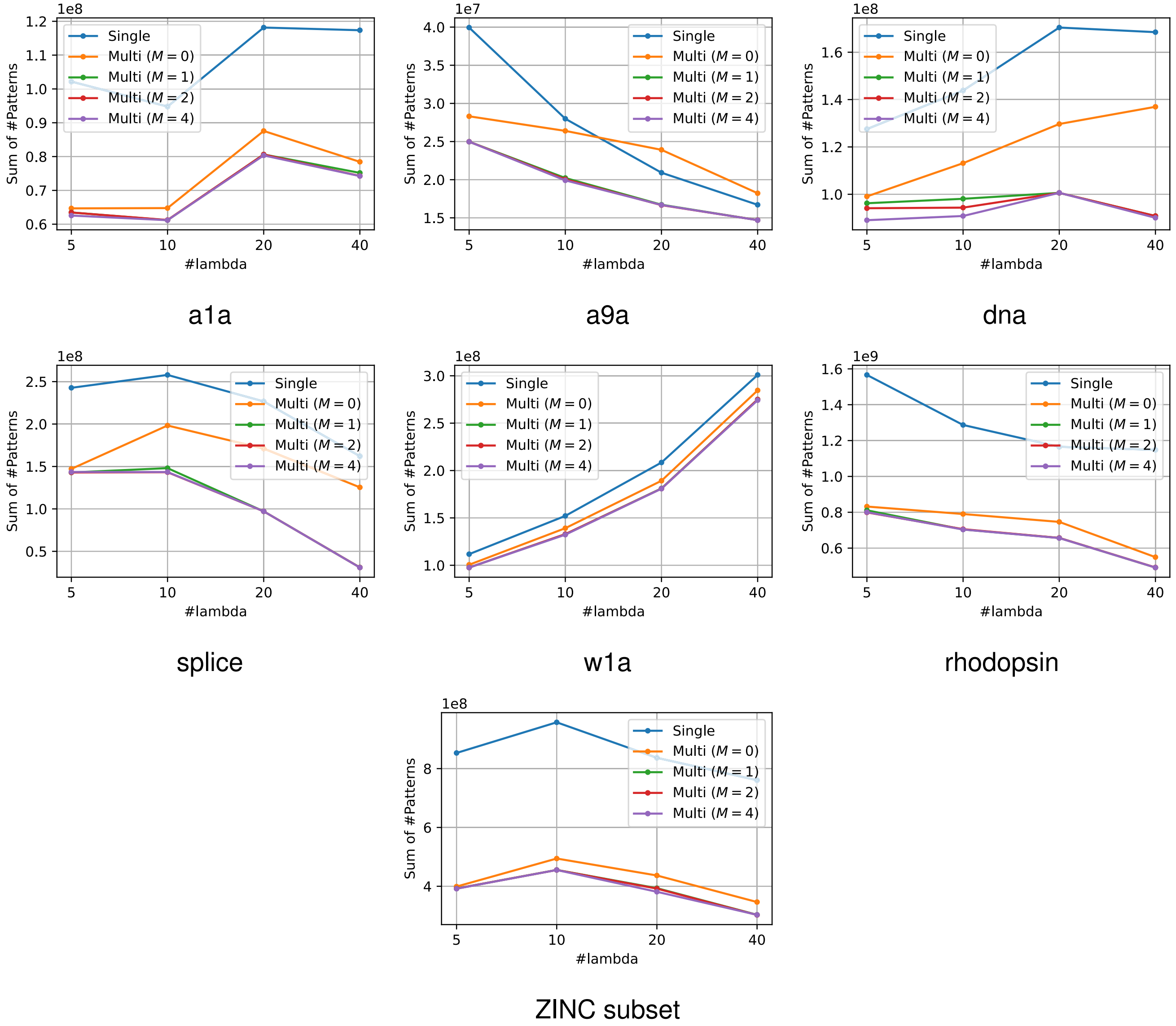

Figure 8. The number of patterns after safe screening during optimization

These values represent the sums over the iterations until convergence across the entire regularization path. Note that for fair comparison, we exclude iterations from the sum where optimization has already converged with some M. Multiple reference screening reduces numerous patterns and multiple dynamic screening (i.e., $M > 0$) reduces the number of patterns further in some cases.

(see "Experimental setup"). As for κ sequence, we investigated the cases with $\kappa \in \{0, 0.01, 0.1, 1.0, 10.0, 100.0\}$. For both single-SPP and multi-SPP, the optimization was performed based on Algorithm 3 in Note S4. In single-SPP, only the optimal solution from the previous λ was used as a reference feasible solution.

The experimental results are presented in Figure 6. From this figure, it can be confirmed that the use of multiple reference solutions is effective in many cases. Moreover, in multi-SPP, dynamic screening can improve performance to some extent even for small values of M. The increase in M did not result in a considerable increase in computational time. The number of patterns remaining after SPP is also displayed in Figure 7. It is confirmed that the use of multiple feasible solutions leads to a significant reduction of the number of patterns. Note that the value of M is irrelevant to pruning, therefore we compared only the single feasible case and multiple feasible case with $M = 0$. Furthermore, to demonstrate the significance of multiple dynamic screening, we show the number of patterns after safe screening during optimization in Figure 8. For fair comparison, we summed the number of patterns over the iterations until convergence, except for the iterations where optimization has already converged with some M. We observe that multiple dynamic screening reduces the number by more than one-half in some cases. Although no improvement in computation speed was observed for w1a, we confirmed that the number of patterns is reduced by our method. Therefore, this may be due to the fact that the additional cost of computing multiple solutions

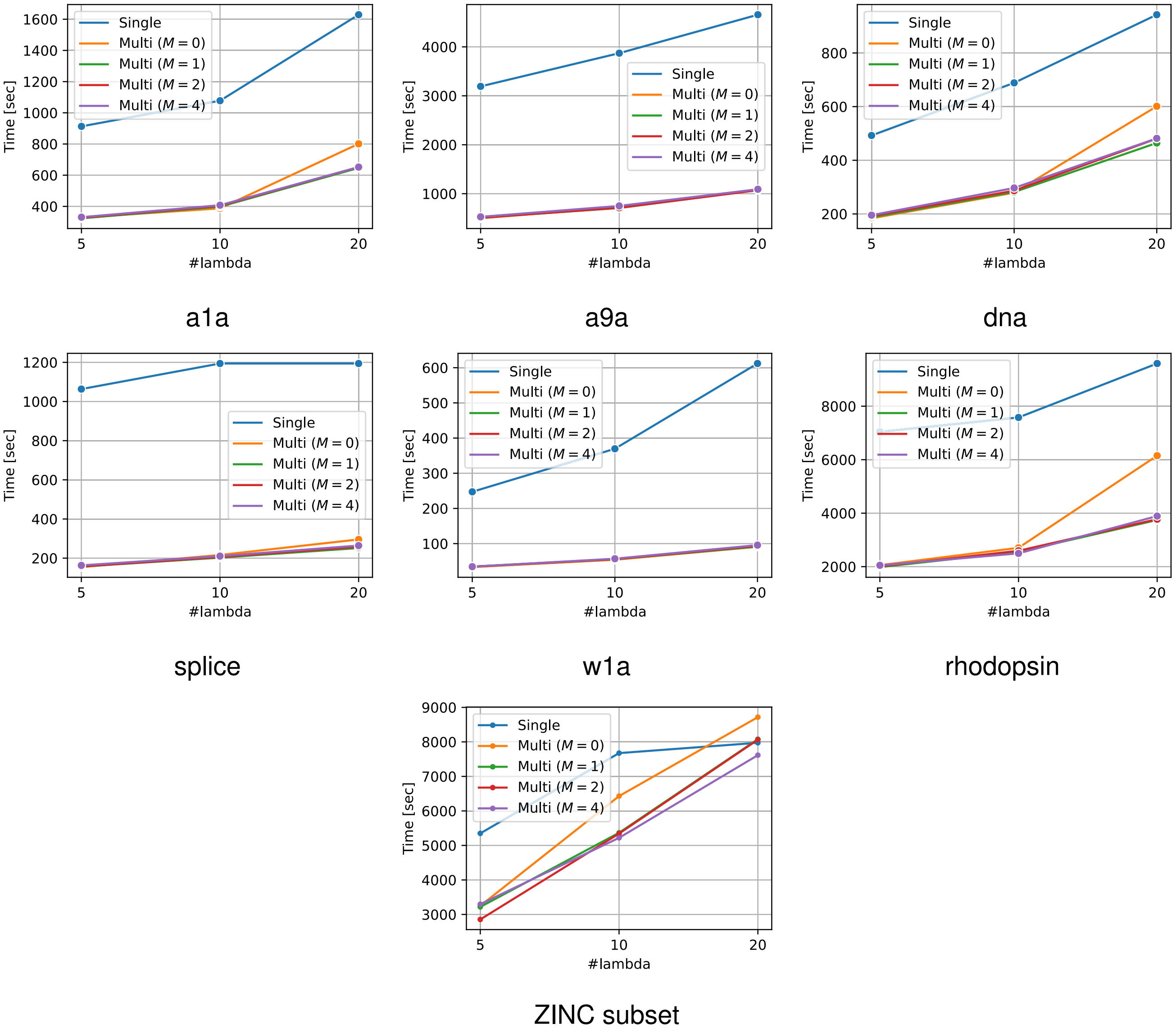

Figure 9. The computation time for leave-one-out CV for each dataset

The use of multiple solutions is effective for all the cases. The multi-dynamic screening is effective in settings where the number of λ is large.

outweighs the benefits of reducing the computation cost with multiple solutions.

SPP with hyperparameter selection by CV

Next, we conducted experiments to investigate the use of reference feasible solutions in the hyperparameter selection process based on CV. To compare the computational costs, we investigated the case where one of the two hyperparameters, κ, was fixed at 0, and only λ was varied as in "SPP with multiple hyperparameter selection." In terms of CV configuration, we compared the computation time of leave one out CV. Specifically, we constructed 10 leave-one-out datasets at random, and compared the relative computational costs of each method option and problem setting. For a single-SPP with one reference feasible solution, we used the optimal solution from the previous λ, as in "SPP with multiple hyperparameter selection."

Figure 9 shows the experimental results. In multi-SPP, a significant reduction in overall computation time can be achieved by using the optimal solution with the entire dataset as a reference feasible solution. The number of patterns after SPP is displayed in Figure 10 and safe screening in Figure 11. From these figures, we can confirm a reduction in the number of patterns in most cases. While there were some cases where dynamic screening in multi-SPP showed some effectiveness, no significant changes in performance were observed in many other cases. We conjecture that this is due to a trade-off between the reduction in computation time resulting from the effectiveness of screening with increasing M and the increase in computation time necessary for updating multiple optimal solutions.

Comparison with boosting-based methods

Finally, we compared the computational costs of the proposed SPP and the existing boosting-based approach in

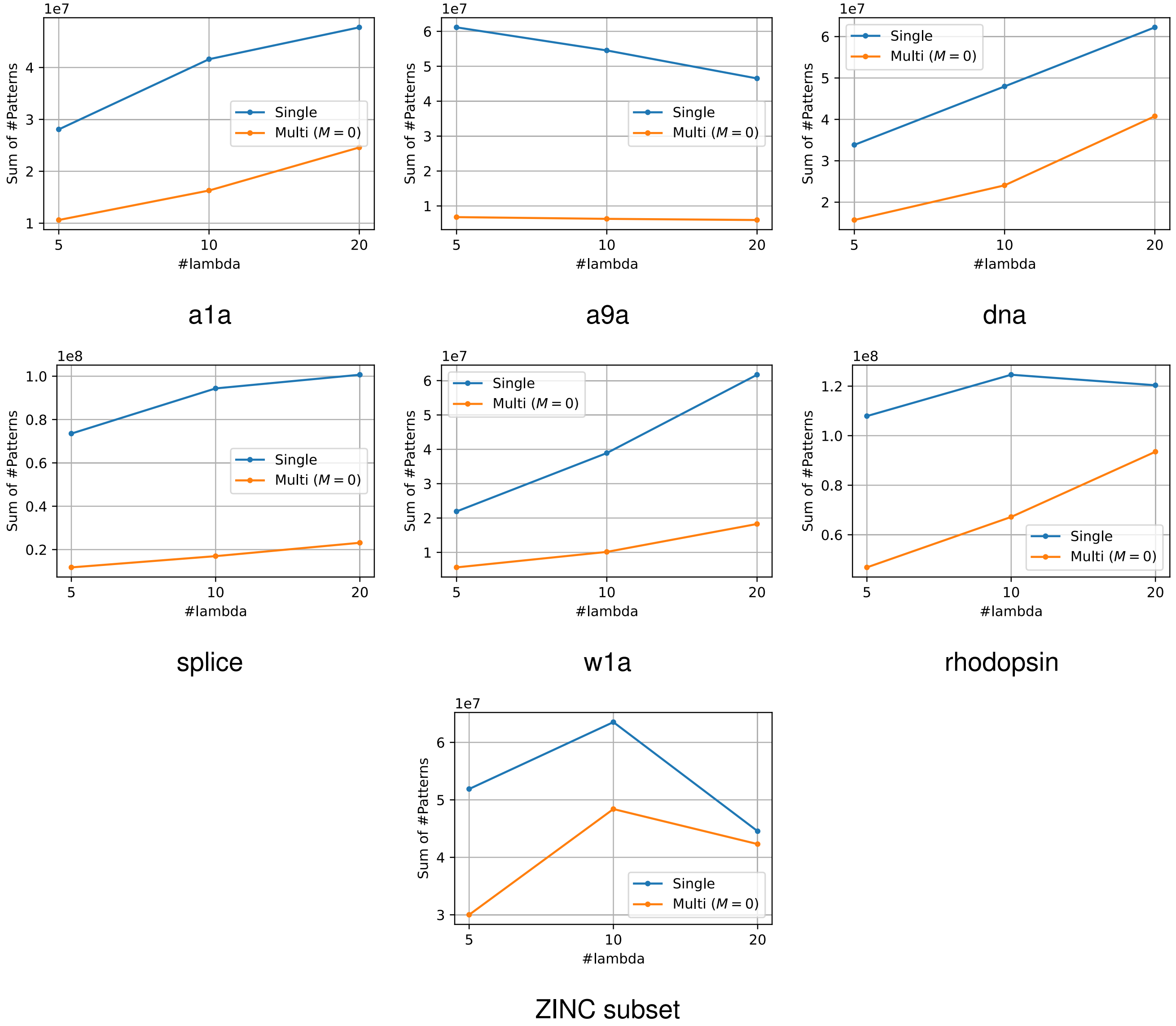

Figure 10. The number of patterns remaining after SPP, before optimization for leave-one-out CV

Similar to the case of multiple hyperparameter selection, our method can eliminate numerous patterns.

predictive pattern mining.[1,7,8] The boosting-based approach involves sequential addition of patterns to the prediction model, necessitating tree traverse search at each step. In contrast, the SPP requires only a single tree traverse search (under fixed regularization parameter), thereby exhibiting a computational advantage.

To conduct fair comparisons, we set the problem such that both methods start from λ_{max} and seek the optimal solution at $0.01\lambda_{max}$. For the boosting-based method, we measured the computational cost taken from the optimal solution at λ_{max}, which does not include any patterns, to adding one pattern at a time until arriving at the optimal solution at $0.01\lambda_{max}$. For the SPP, we considered the one-dimensional regularization path from λ_{max} to $0.01\lambda_{max}$ and measured the computational cost when performing the same process as in "SPP with multiple hyperparameter selection." Both methods used the coordinate gradient descent method[54] for optimization, and the another hyperparameter κ was set to 0.

First, we considered the problems of graph classification and graph regression with chemical compound datasets as examples in predictive graph mining. Specifically, we used two datasets for graph classification: CPDB ("Helma CPDB Mutagenicity Subset," $n = 684$) and Mutagenicity ("Bursi Mutagenicity Dataset," $n = 4337$), and two datasets for graph regression: Bergstrom ("Bergstrom Melting Point Dataset," $n = 185$) and Karthikeyan ("Karthikeyan Melting Point Dataset," $n = 4450$). All datasets were retrieved from http://cheminformatics.org/datasets/. Note that, since these datasets were downloaded when our preliminary work[33] was conducted, and the websites above were closed later, we also present the link to the archived website: http://web.archive.org/web/20150503130239/http://cheminformatics.org/datasets/. In addition, since the number

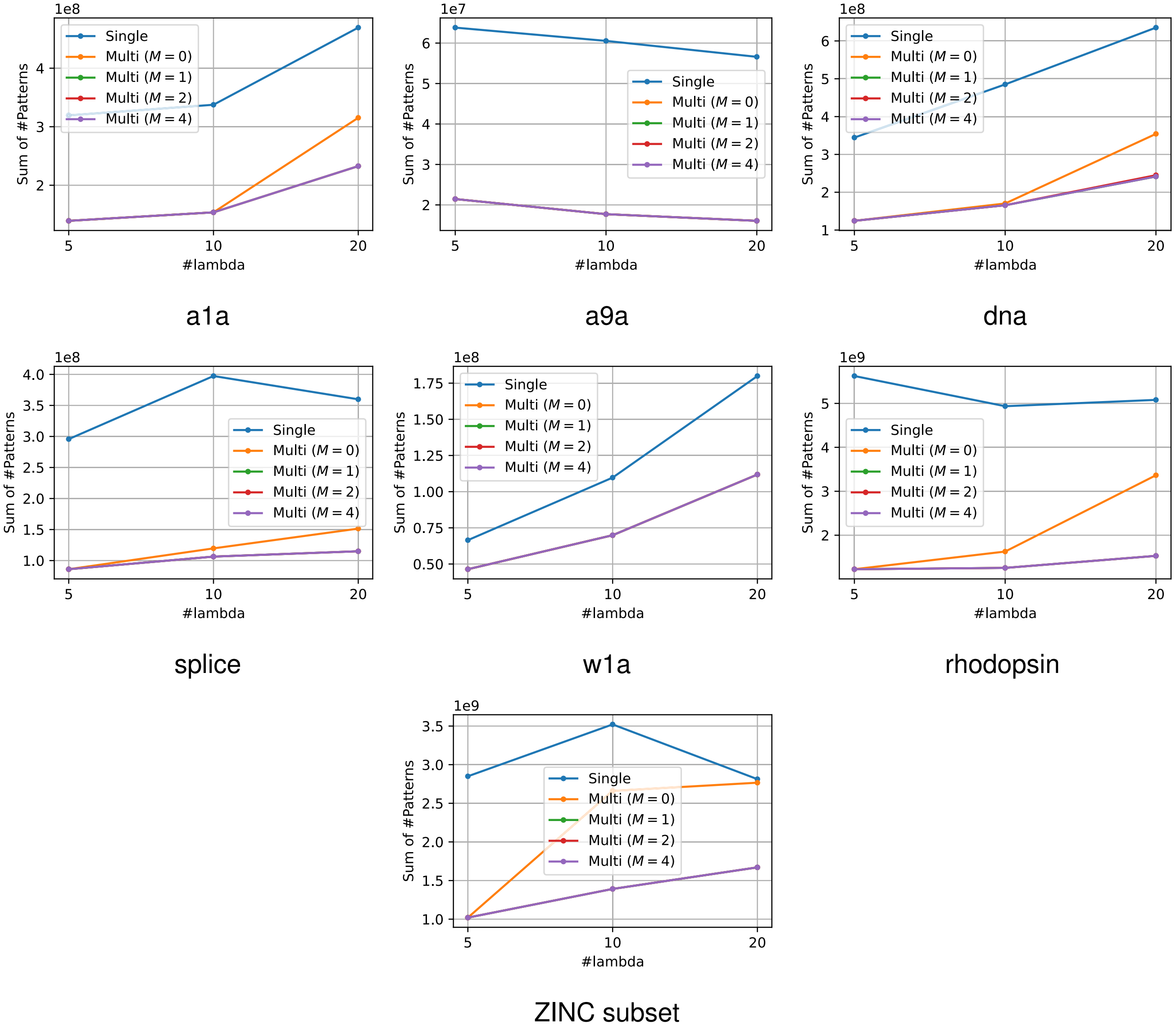

Figure 11. The number of patterns remaining after safe screening during optimization for leave-one-out CV

Similar to Figure 8, multiple feasible screening eliminates numerous patterns, while multiple dynamic screening reduces the number of patterns further, particularly in cases where the number of λ s is smaller in some datasets.

of instances n were mistakenly noted in the preliminary work, we noted collect numbers. Figure 12 shows the computational cost of the boosting-based method (boosting) and the SPP. In all the cases, SPP is faster than boosting, and the differences become more significant as the maximum length of patterns increases. The results also indicate that the tree traverse time are not so different between the two methods. We conjecture that this is because the most time-consuming part of gSpan is the isomorphism check, which is required to avoid enumerating duplicated graphs.

Next, we considered classification and regression in item set predictive mining. We used two datasets for classification: splice ($n = 1000$) and a9a ($n = 32561$), and two datasets for regression: dna ($n = 2000$) and protein ($n = 6621$). The results are shown in Figure 13. In all the cases, SPP was faster than boosting. Unlike graph mining cases, the tree traverse time of the SPP was smaller than that of boosting-based method. We conjecture that this is because boosting-based methods require multiple enumeration of patterns, while the SPP requires only one enumeration.

DISCUSSION

Structured data such as sets, graphs, and sequences are common in many fields, and it is necessary to develop machine learning methods to handle such structure data. Although neural networks for structured data have seen significant recent development and can achieve good predictive performances, practical problems often require interpretation and explanation of

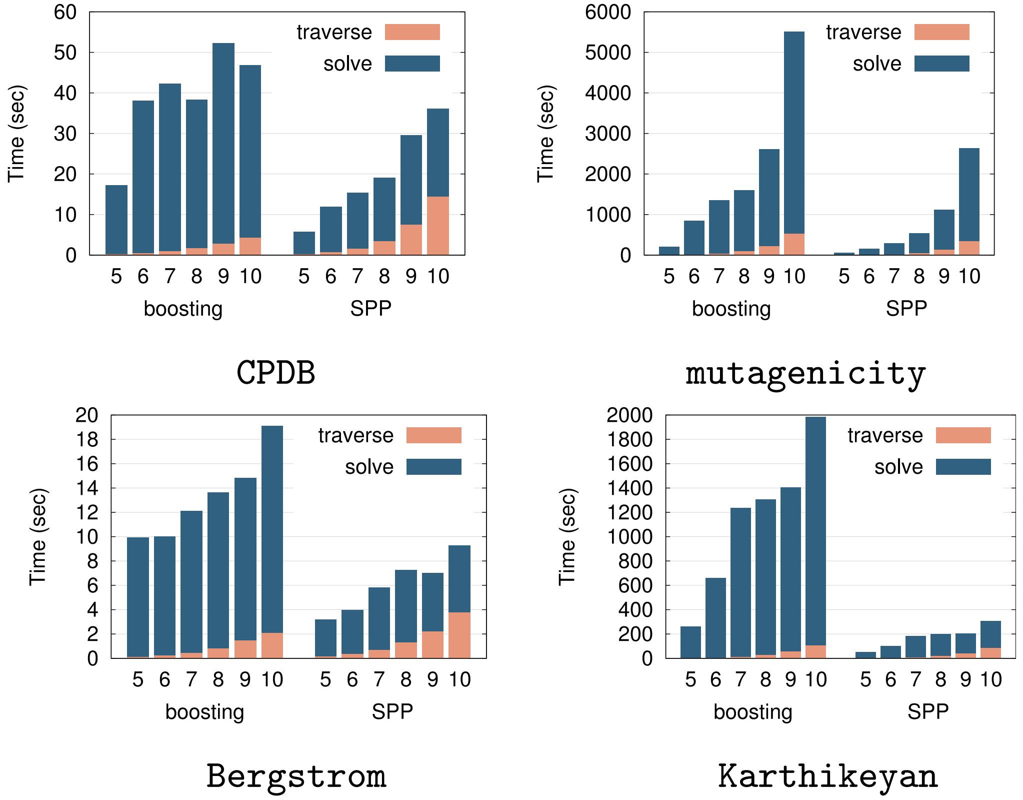

Figure 12. Computational time comparison for graph classification and regression

The horizontal axis represents the maximum length of patterns that are mined. Each bar contains computational time taken in the tree traverse (traverse) and the optimization procedure (solve) respectively. Same figure as our preliminary work.[33]

the model behavior and extracting important features. In this study, we propose a pattern mining approach for machine learning modeling of structured data that can both prediction ability and explainability.

The challenge of extracting features from structural data lies in the exponential increase in the number of potential unified inconsistent notation sub-structures that can serve as features. This difficulty has led to the development of various pattern mining algorithms, particularly in the task of enumeration. However, research that integrates pattern mining with predictive modeling, such as regression or classification, is limited. To our knowledge, only boosting-based approaches have been proposed, which are inefficient due to the requirement of a tree traverse for each additional feature.

In this study, we addressed a common problem in the field of pattern mining by introducing safe screening. Safe screening is a technique developed in the field of sparse modeling that allows for the identification of redundant features before solving the optimization problem, which can significantly reduce the computational cost. However, as noted in the "Introduction" and "SPP," applying conventional safe screening to many patterns is not feasible. To address this, we proposed the SPP method, which efficiently handles multiple features in a single tree traverse, as opposed to a boosting-based approach. By leveraging the monotonicity of patterns, as described in lemma 3, the proposed SPP method can safely eliminate a subset of patterns with only a moderate increase in computational cost.

For a comparison with recent methods, there are several papers which utilize GAP safe screening as described in "Sparse learning and safe screening." The extension to special regularizers such as non-convex penalty are proposed.[49,50] Our concept of using multiple solutions can be incorporated into these methods and can accelerate their optimizations. Several studies exist in the paradigm of stochastic gradient method.[52,53] Although it is not clear that our method can be directly applied to them, this topic is worth discussing in future work. Additionally, the sphere refinement technique[51] can further accelerate SPP more in some loss functions. We would like to demonstrate its effectiveness in the context of predictive pattern mining in the future.

Finally, we demonstrated the effectiveness of the proposed SPP method in the entire model building process, including hyperparameter selection and CV in this paper. Especially, it has been observed that the SPP method works more efficiently if two reference feasible solutions are available, as experimentally demonstrated in "SPP with multiple hyperparameter selection" and "SPP with hyperparameter selection by CV." Additionally, in "Multiple dynamic screening with SPP," we discussed that the parameter M, which determines the number of dynamic safe screening applications with multiple solutions, needs to be appropriately chosen. The experiments in "SPP with multiple hyperparameter selection" and "SPP with hyperparameter selection by CV" have shown that not only small but also large values of M may increase computational costs. In our

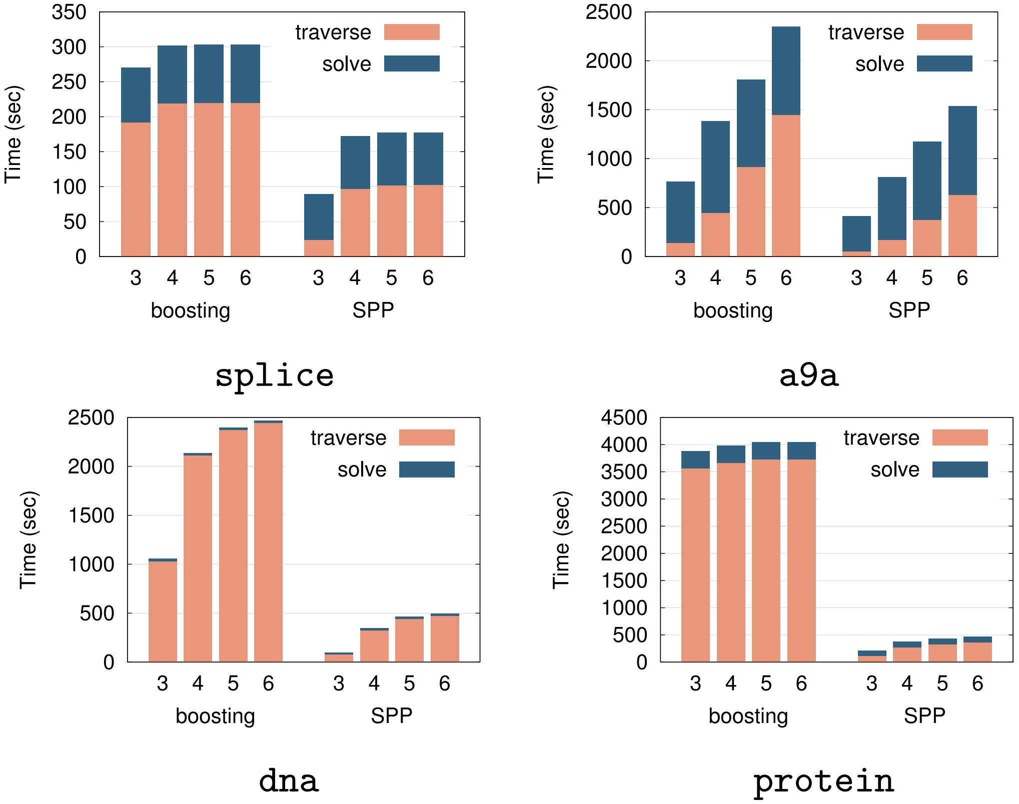

Figure 13. Computational time comparison for item set classification and regression

The horizontal axis represents the maximum length of patterns that are mined. Each bar contains computational time taken in the tree traverse (traverse) and the optimization procedure (solve) respectively. Same figure as our preliminary work.[33]

experiments, it is demonstrated that our method performed well at $M = 1$; however, a more general selection criterion is a topic for future work.

EXPERIMENTAL PROCEDURES

Resource availability

Lead contact

Further information and requests for resources and reagents should be directed to and will be fulfilled by the lead contact, Ichiro Takeuchi (ichiro.takeuchi@mae.nagoya-u.ac.jp).

Materials availability

This study did not generate new unique reagents.

Data and code availability

The code for "SPP with multiple hyperparameter selection" and "SPP with hyperparameter selection by CV" has been deposited at Zenodo under the DOI https://doi.org/10.5281/zenodo.10017314, and the code for "Comparison with boosting-based methods" (same as our preliminary work[33]) is at Zenodo under the DOI https://doi.org/10.5281/zenodo.10017836. They are publicly available as of the date of publication.

Any additional information required to reanalyze the data reported in this paper is available from the lead contact upon request.

Experimental setup

First, we describe the settings common to all the experiments. We compared the computation time of the entire or partial regularization path with respect to the hyperparameters λ and/or κ. Regarding λ, we defined λ_{max} as the largest value of λ for which $\exists j \in [d], \beta_j^* \neq 0$, and constructed a sequence of λs by partitioning the interval from λ_{max} to $0.01\lambda_{max}$ into equally spaced values on a logarithmic scale, where the number of partitions is varied depending on the experimental options. The datasets used in the experiments in "SPP with multiple hyperparameter selection" and "SPP with hyperparameter selection by CV" are presented in Table 1. We used the squared hinge loss function as L for classification problem, and the squared loss function for regression. The coordinate descent method was employed for optimization, with a convergence criterion $\epsilon = 10^{-4}$. During optimization, dynamic screening was performed every other iteration for the first $T = 5$ cycles, and subsequently executed once every ten iterations. PrefixSpan[55] was employed as the mining algorithm for both set and sequence mining tasks, while gSpan[56] was used for graph mining tasks.

SUPPLEMENTAL INFORMATION

Supplemental information can be found online at https://doi.org/10.1016/j.patter.2023.100890.

ACKNOWLEDGMENTS

This work was partially supported by MEXT KAKENHI (20H00601), JST CREST (JPMJCR21D3 and JPMJCR22N2), JST Moonshot R&D (JPMJMS2033-05), JST AIP Acceleration Research (JPMJCR21U2), NEDO (JPNP18002 and JPNP20006), and the RIKEN Center for Advanced Intelligence Project.

AUTHOR CONTRIBUTIONS

Conceptualization, T.Y., K.N., K. Tsuda, and I.T.; methodology, T.Y., H.H., K.N., K. Taji, and I.T.; software, T.Y. and K.N.; validation, T.Y. and H.H.; formal analysis, T.Y., H.H., and I.T.; writing – original draft, T.Y., H.H., K.N., and I.T.; writing – review and editing, H.H., K. Taji, K. Tsuda, and I.T.; visualization,

Table 1. The list of datasets used in the experiments

Dataset	No.	Structure type	Maximum length of patterns	Problem
a1a	1,605	item set	5	classification
a9a	32,561	item set	5	classification
dna	2,000	item set	3	regression
splice	1,000	item set	3	classification
w1a	2,477	item set	3	classification
rhodopsin	1,162	sequence	50	regression
ZINC subset	500	graph	10	regression

Datasets used in the experiments in "Numerical experiments." The dataset rhodopsin is studied in Inoue et al.[57] and Karasuyama et al.[58] The dataset ZINC subset is a random sample from ZINC database[59] with molecular weights between 450 and 500, and the objective variable is "logP" (logarithm of partition coefficient). The remaining datasets are listed in Chang and Lin.[60]

T.Y. and K.N.; supervision, H.H. and I.T.; project administration, H.H. and I.T.; and funding acquisition, I.T.

DECLARATION OF INTERESTS

The authors declare no competing interests.

DECLARATION OF GENERATIVE AI AND AI-ASSISTED TECHNOLOGIES IN THE WRITING PROCESS

During the preparation of this work the authors used ChatGPT to make English representation more appropriate. After using this tool, the authors reviewed and edited the content as needed and take full responsibility for the content of the publication.

Received: April 28, 2023
Revised: November 2, 2023
Accepted: November 9, 2023
Published: December 1, 2023

REFERENCES

1. Saigo, H., Uno, T., and Tsuda, K. (2007). Mining complex genotypic features for predicting hiv-1 drug resistance. Bioinformatics *23*, 2455–2462.
2. Le Duy, V.N., Sakuma, T., Ishiyama, T., Toda, H., Arai, K., Karasuyama, M., Okubo, Y., Sunaga, M., Hanada, H., Tabei, Y., and Takeuchi, I. (2022). Stat-dsm: Statistically discriminative sub-trajectory mining with multiple testing correction. IEEE Trans. Knowl. Data Eng. *34*, 1477–1488.
3. Hazimeh, H., and Mazumder, R. (2020). Learning hierarchical interactions at scale: A convex optimization approach. In Proceedings of the Twenty Third International Conference on Artificial Intelligence and Statistics (PMLR), pp. 1833–1843.
4. Bunker, R., Fujii, K., Hanada, H., and Takeuchi, I. (2021). Supervised sequential pattern mining of event sequences in sport to identify important patterns of play: an application to rugby union. PLoS One *16*, e0256329.
5. Das, D., Le Duy, V.N., Hanada, H., Tsuda, K., and Takeuchi, I. (2022). Fast and more powerful selective inference for sparse high-order interaction model. In Proceedings of the AAAI Conference on Artificial Intelligence, K. Sycara, V. Honaver, and M. Spaan, eds., pp. 9999–10007.
6. Kato, H., Hanada, H., and Takeuchi, I. (2022). Safe rulefit: Learning optimal sparse rule model by meta safe screening. In IEEE Transactions on Pattern Analysis and Machine Intelligence, pp. 2330–2343.
7. Saigo, H., Kadowaki, T., and Tsuda, K. (2006). A linear programming approach for molecular qsar analysis. In Proceedings of the International Workshop on Mining and Learning with Graphs 2006 (MLG 2006), pp. 85–96.
8. Saigo, H., Nowozin, S., Kadowaki, T., Kudo, T., and Tsuda, K. (2009). gboost: a mathematical programming approach to graph classification and regression. Mach. Learn. *75*, 69–89.
9. Duverle, D.A., Takeuchi, I., Murakami-Tonami, Y., Kadomatsu, K., and Tsuda, K. (2013). Discovering combinatorial interactions in survival data. Bioinformatics *29*, 3053–3059.
10. Suzumura, S., Nakagawa, K., Umezu, Y., Tsuda, K., and Takeuchi, I. (2017). Selective inference for sparse high-order interaction models. In ICML '17: Proceedings of the 34th International Conference on Machine Learning, D. Precup and Y.W. Teh, eds. (PMLR), pp. 3338–3347.
11. Takayanagi, M., Tabei, Y., and Saigo, H. (2018). Entire regularization path for sparse nonnegative interaction model. In IEEE International Conference on Data Mining (ICDM) (IEEE), pp. 1254–1259.
12. Yoshida, T., Takeuchi, I., and Karasuyama, M. (2018). Safe triplet screening for distance metric learning. In KDD '18: Proceedings of the 24th ACM SIGKDD International Conference on Knowledge Discovery & Data Mining, Y. Guo and F. Farooq, eds. (Association for Computing Machinery), pp. 2653–2662.
13. Le Morvan, M., and Vert, J.-P. (2018). Whinter: A working set algorithm for high-dimensional sparse second order interaction models. In Proccedings of the 35th International Conference on Machine Learning (PMLR), pp. 3635–3644.
14. Yoshida, T., Takeuchi, I., and Karasuyama, M. (2019). Learning interpretable metric between graphs: Convex formulation and computation with graph mining. In KDD '19: Proceedings of the 25th ACM SIGKDD International Conference on Knowledge Discovery & Data Mining, A. Teredesai and V. Kumar, eds. (Association for Computing Machinery), pp. 1026–1036.
15. Gärtner, T., Flach, P., and Wrobel, S. (2003). On graph kernels: Hardness results and efficient alternatives. In Learning Theory and Kernel Machines. Lecture Notes in Computer Science, B. Schölkopf and M.K. Warmuth, eds. (Springer), pp. 129–143.
16. Kashima, H., Tsuda, K., and Inokuchi, A. (2003). Marginalized kernels between labeled graphs. In ICML '03: Proceedings of the Twentieth International Conference on Cachine Learning, T. Fawcett and N. Mishra, eds. (AAAI Press), pp. 321–328.
17. Vishwanathan, S.V.N., Schraudolph, N.N., Kondor, R., and Borgwardt, K.M. (2010). Graph kernels. J. Mach. Learn. Res. *11*, 1201–1242.
18. Shervashidze, N., Vishwanathan, S., Petri, T., Mehlhorn, K., and Borgwardt, K. (2009). Efficient graphlet kernels for large graph comparison. In Proceedings of the Twelfth International Conference on Artificial Intelligence and Statistics (PMLR), pp. 488–495.
19. Tsuda, K., Kin, T., and Asai, K. (2002). Marginalized kernels for biological sequences. Bioinformatics *18*, S268–S275.
20. Lodhi, H., Saunders, C., Shawe-Taylor, J., Cristianini, N., and Watkins, C. (2002). Text classification using string kernels. J. Mach. Learn. Res. *2*, 419–444.
21. Leslie, C., Eskin, E., and Noble, W.S. (2001). The spectrum kernel: A string kernel for svm protein classification. In Pacific Symposium on Biocomputing 2002, R.B. Altman, A.K. Dunker, L. Hunter, K. Lauderdale, and T.E. Klein, eds. (World Scientific), pp. 564–575.
22. Qi, C.R., Su, H., Mo, K., and Guibas, L.J. (2017). Pointnet: Deep learning on point sets for 3d classification and segmentation. In 2017 IEEE Conference on Computer Vision and Pattern Recognition CVPR, pp. 77–85.
23. Lee, J., Lee, Y., Kim, J., Kosiorek, A., Choi, S., and Teh, Y.W. (2019). Set transformer: A framework for attention-based permutation-invariant neural networks. In Proceedings of the 36th International Conference on Machine Learning (PMLR), pp. 3744–3753.
24. Scarselli, F., Gori, M., Tsoi, A.C., Hagenbuchner, M., and Monfardini, G. (2009). The graph neural network model. IEEE Trans. Neural Netw. *20*, 61–80.

CellPress
OPEN ACCESS

25. Kipf, T.N., and Welling, M. (2016). Semi-supervised classification with graph convolutional networks. Preprint at arXiv.
26. Veličković, P., Cucurull, G., Casanova, A., Romero, A., Lio, P., and Bengio, Y. (2017). Graph attention networks. Preprint at openreview.net.
27. Zhou, J., Cui, G., Hu, S., Zhang, Z., Yang, C., Liu, Z., Wang, L., Li, C., and Sun, M. (2020). Graph neural networks: A review of methods and applications. AI open *1*, 57–81.
28. Xu, K., Hu, W., Leskovec, J., and Jegelka, S. (2018). How powerful are graph neural networks?. Preprint at arXiv.
29. Hochreiter, S., and Schmidhuber, J. (1997). Long short-term memory. Neural Comput. *9*, 1735–1780.
30. Graves, A. (2013). Generating sequences with recurrent neural networks. Preprint at arXiv.
31. Sutskever, I., Vinyals, O., and Le, Q.V. (2014). Sequence to sequence learning with neural networks. In NIPS '14: Proceedings on the 27th International Conference on Neural Information Processing Systems, Z. Ghahramani, M. Welling, C. Cortes, N.D. Lawrence, and K.Q. Weinberger, eds. (MIT Press), pp. 3104–3112.
32. Vaswani, A., Shazeer, N., Parmar, N., Uszkoreit, J., Jones, L., Gomez, A.N., Kaiser, Ł., and Polosukhin, I. (2017). Attention is all you need. In NIPS '17: Proceedings of the 31st International Conference on Neural Information Processing Systems, U. von Luxburg and I. Guyon, eds. (Curran Associates Inc.), pp. 6000–6010.
33. Nakagawa, K., Suzumura, S., Karasuyama, M., Tsuda, K., and Takeuchi, I. (2016). Safe pattern pruning: An efficient approach for predictive pattern mining. In KDD '16: Proceedings of the 22nd ACM SIGKDD International Conference on Knowledge Discovery and Data Mining, B. Krishnapuram and M. Shah, eds. (Association for Computing Machinery), pp. 1785–1794.
34. Zou, H., and Hastie, T. (2005). Regularization and variable selection via the elastic net. J. Roy. Stat. Soc. B *67*, 301–320.
35. Rockafellar, R.T. (1997). Convex Analysis (Princeton University Press).
36. Ghaoui, L.E., Viallon, V., and Rabbani, T. (2010). Safe feature elimination for the lasso and sparse supervised learning problems. Preprint at arXiv.
37. Wang, J., Zhou, J., Wonka, P., and Ye, J. (2013). Lasso screening rules via dual polytope projection. In NIPS '13: Proceedings of the 26th International Conference on Neural Information Processing Systems, C.J.C. Burges, L. Bottou, M. Welling, Z. Ghahramani, and K.Q. Weinberger, eds. (Curran Associates Inc.), pp. 1070–1078.
38. Fercoq, O., Gramfort, A., and Salmon, J. (2015). Mind the duality gap: safer rules for the lasso. In ICML '15: Proceedings of the 32nd International Conference on Machine Learning, F. Bach and D. Bei, eds. (PMLR), pp. 333–342.
39. Ndiaye, E., Fercoq, O., Gramfort, A., and Salmon, J. (2017). Gap safe screening rules for sparsity enforcing penalties. J. Mach. Learn. Res. *18*, 4671–4703.
40. Xiang, Z.J., Wang, Y., and Ramadge, P.J. (2017). Screening tests for lasso problems. IEEE Trans. Pattern Anal. Mach. Intell. *39*, 1008–1027.
41. Bonnefoy, A., Emiya, V., Ralaivola, L., and Gribonval, R. (2015). Dynamic screening: Accelerating first-order algorithms for the lasso and group-lasso. IEEE Trans. Signal Process. *63*, 5121–5132.
42. Ogawa, K., Suzuki, Y., and Takeuchi, I. (2013). Safe screening of non-support vectors in pathwise svm computation. In ICML '13: Proceedings of the 30th International Conference on Machine Learning, S. Dasgupta and D. McAllester, eds. (PMLR), pp. 1382–1390.
43. Shibagaki, A., Suzuki, Y., Karasuyama, M., and Takeuchi, I. (2015). Regularization path of cross-validation error lower bounds. In NIPS '15: Proceedings on the 28th International Conference on Neural Information Processing Systems, C. Cortes, D.D. Lee, M. Sugiyama, and R. Garnett, eds. (MIT Press), pp. 1675–1683.
44. Shibagaki, A., Karasuyama, M., Hatano, K., and Takeuchi, I. (2016). Simultaneous safe screening of features and samples in doubly sparse modeling. In ICML '16: Proceedings of the 33rd International Conference on Machine Learning, M.F. Balcan and K.Q. Weinberger, eds. (PMLR), pp. 1577–1586.
45. Hanada, H., Shibagaki, A., Sakuma, J., and Takeuchi, I. (2018). Efficiently monitoring small data modification effect for large-scale learning in changing environment. In AAAI '18/IAAI '18/EAAI '18: Proceedings of the Thirty-Second AAAI Conference on Artificial Intelligence and Thirtieth Innovative Applications of Artificial Intelligence Conference and EigghthAAAI Symposium on Educational Advances in Artificial Intelligence, S.A. McIlraith and K.Q. Weinberger, eds. (AAAI Press), pp. 1314–1321.
46. Okumura, S., Suzuki, Y., and Takeuchi, I. (2015). Quick sensitivity analysis for incremental data modification and its application to leave-one-out cv in linear classification problems. In KDD '15: Proceedings of the 21th ACM SIGKDD International Conference on Knowledge Discovery and Data Mining, L. Cao and C. Zhang, eds. (Association for Computing Machinery), pp. 885–894.
47. Ndiaye, E., Le, T., Fercoq, O., Salmon, J., and Takeuchi, I. Safe grid search with optimal complexity. Preprint at arXiv.
48. Takada, T., Hanada, H., Yamada, Y., Sakuma, J., and Takeuchi, I. Secure approximation guarantee for cryptographically private empirical risk minimization. Preprint at arXiv.
49. Rakotomamonjy, A., Gasso, G., and Salmon, J. Screening rules for lasso with non-convex sparse regularizers. Preprint at arXiv.
50. Bao, R., Gu, B., and Huang, H. (2020). Fast oscar and owl regression via safe screening rules. In ICML '20: Proceedings of the 37th International Conference on Machine Learning, H. Duame and A. Singh, eds. (PMLR), pp. 653–663.
51. Dantas, C.F., Soubies, E., and Févotte, C. (2021). Expanding boundaries of gap safe screening. J. Mach. Learn. Res. *22*, 10665–10721.
52. Bao, R., Gu, B., and Huang, H. (2022). An accelerated doubly stochastic gradient method with faster explicit model identification. In CIKM '22: Proceedings of the 31st ACM International Conference on Information & Knowledge Management, M. Al Hasan and L. Xiong, eds. (Association for Computing Machinery), pp. 57–66.
53. Bao, R., Wu, X., Xian, W., and Huang, H. (2022). Doubly sparse asynchronous learning for stochastic composite optimization. In Proceedings of the Thirty-First International Joint Conference on Artificial Intelligence (IJCAI), pp. 1916–1922.
54. Tseng, P., and Yun, S. (2009). A coordinate gradient descent method for nonsmooth separable minimization. Math. Program. *117*, 387–423.
55. Pei, J., Han, J., Mortazavi-Asl, B., Pinto, H., Chen, Q., Dayal, U., and Hsu, M. (2001). Prefixspan: Mining sequential patterns efficiently by prefix-projected pattern growth. In Proceedings of the 17th International Conference on Data Engineering (IEEE), pp. 215–224.
56. Yan, X., and Han, J. (2002). Gspan: Graph-based substructure pattern mining. In 2002 IEEE International Conference on Data Mining (IEEE), pp. 721–724.
57. Inoue, K., Karasuyama, M., Nakamura, R., Konno, M., Yamada, D., Mannen, K., Nagata, T., Inatsu, Y., Yawo, H., Yura, K., et al. (2021). Exploration of natural red-shifted rhodopsins using a machine learning-based bayesian experimental design. Commun. Biol. *4*, 362.
58. Karasuyama, M., Inoue, K., Nakamura, R., Kandori, H., and Takeuchi, I. (2018). Understanding colour tuning rules and predicting absorption wavelengths of microbial rhodopsins by data-driven machine-learning approach. Sci. Rep. *8*, 15580.
59. Sterling, T., and Irwin, J.J. (2015). Zinc 15–ligand discovery for everyone. J. Chem. Inf. Model. *55*, 2324–2337.
60. Chang, C.-C., and Lin, C.-J. (2011). Libsvm: a library for support vector machines. ACM Trans. Intell. Syst. Technol. *2*, 1–27.

Patterns, Volume *4*

Supplemental information

Efficient model selection for predictive pattern mining model by safe pattern pruning

Takumi Yoshida, Hiroyuki Hanada, Kazuya Nakagawa, Kouichi Taji, Koji Tsuda, and Ichiro Takeuchi

Supplemental note S1: Proof of Lemma 2

Proofs of the following lemmas are similar to previous studies[1,2], however, we explicitly state the proof for our setup.

Before proving Lemma 2, we prove two other lemmas: Lemmas 7 and 8.

Definition 6. *For a convex function $f : \mathbb{R}^n \to \mathbb{R}$ and a vector $\boldsymbol{v} \in \mathbb{R}^n$, $\boldsymbol{g} \in \mathbb{R}^n$ is called a* subderivative *of f at $\boldsymbol{v}$ if the following condition is met:*

$$\forall \boldsymbol{z} : \quad f(\boldsymbol{z}) - f(\boldsymbol{v}) \geq \boldsymbol{g}^\top (\boldsymbol{z} - \boldsymbol{v}).$$

We denote by $\partial f(\boldsymbol{v})$ the set of all subderivatives of f at $\boldsymbol{v}$, since such $\boldsymbol{g}$ may not be unique.

Lemma 7. *For any pair of feasible solutions $(\tilde{\boldsymbol{\beta}}, \tilde{\boldsymbol{\alpha}})$, the dual optimal solution $\boldsymbol{\alpha}^*$ is within the intersection of a $\mathbb{R}^n$-ball $B(\tilde{\boldsymbol{\beta}}, \tilde{\boldsymbol{\alpha}})$ and a hyperplane H such that*

$$\begin{aligned} B(\tilde{\boldsymbol{\beta}}, \tilde{\boldsymbol{\alpha}}) &:= \{\boldsymbol{\alpha} \in \mathbb{R}^n \mid \|\boldsymbol{\alpha} - \tilde{\boldsymbol{\alpha}}\|_2 \leq r(\tilde{\boldsymbol{\beta}}, \tilde{\boldsymbol{\alpha}})\}, \\ H &:= \{\boldsymbol{\alpha} \in \mathbb{R}^n \mid \boldsymbol{\alpha}^\top \mathbf{1} = 0\}, \end{aligned} \tag{12}$$

where $r(\tilde{\boldsymbol{\beta}}, \tilde{\boldsymbol{\alpha}})$ and γ are defined in Lemma 2.

Proof. The constraint of H is just derived from (4), so we focus on B in the remainder of the proof.

First, since we assume that the loss function L is γ-Lipschitz continuous, $L^*(\boldsymbol{\alpha})$ is known to be $(1/\gamma)$-*strongly convex* and therefore $D(\boldsymbol{\alpha})$ is $(1/\gamma)$-*strongly concave*, that is, the following holds:

$$D(\boldsymbol{v}) - D(\boldsymbol{u}) \geq \boldsymbol{g}^\top (\boldsymbol{v} - \boldsymbol{u}) + \frac{1}{2\gamma} \|\boldsymbol{v} - \boldsymbol{u}\|_2^2 \tag{13}$$

for any $\boldsymbol{u}, \boldsymbol{v} \in \mathbb{R}^n$ and any subderivative $\boldsymbol{g} \in \partial D(\boldsymbol{v})$. See[3] for example.

Substituting $\boldsymbol{u} \leftarrow \tilde{\boldsymbol{\alpha}}$ and $\boldsymbol{v} \leftarrow \boldsymbol{\alpha}^*$, we have

$$\begin{aligned} D(\boldsymbol{\alpha}^*) - D(\tilde{\boldsymbol{\alpha}}) &\geq \boldsymbol{g}^\top (\boldsymbol{\alpha}^* - \tilde{\boldsymbol{\alpha}}) + \frac{1}{2\gamma} \|\boldsymbol{\alpha}^* - \tilde{\boldsymbol{\alpha}}\|_2^2 \\ &\geq \frac{1}{2\gamma} \|\boldsymbol{\alpha}^* - \tilde{\boldsymbol{\alpha}}\|_2^2. \end{aligned} \tag{14}$$

Here, (14) is obtained by the fact that $\boldsymbol{g}^\top (\boldsymbol{\alpha}^* - \tilde{\boldsymbol{\alpha}}) \geq 0$ (See Proposition B.24 of[4] for example).

Moreover, by the calculation of the dual problem by Fenchel's duality theorem, $P(\boldsymbol{\beta}^*) = D(\boldsymbol{\alpha}^*)$ holds for (2) and (4) (called the *strong duality*; See[3] for example). Therefore, from (14) we have

$$\begin{aligned} \frac{1}{2\gamma} \|\boldsymbol{\alpha}^* - \tilde{\boldsymbol{\alpha}}\|_2^2 &\leq D(\boldsymbol{\alpha}^*) - D(\tilde{\boldsymbol{\alpha}}) = P(\boldsymbol{\beta}^*) - D(\tilde{\boldsymbol{\alpha}}) \\ &\leq P(\tilde{\boldsymbol{\beta}}) - D(\tilde{\boldsymbol{\alpha}}) \quad (\because\ \boldsymbol{\beta}^* \text{ is the minimizer of } P). \end{aligned}$$

This proves the lemma. ■

Lemma 8. *Under the condition of the dual optimal solution $\boldsymbol{\alpha}^*$ in Lemma 7, we can represent an upper bound of $|X_{:j}^\top \boldsymbol{\alpha}^*|$ as*

$$|X_{:j}^\top \boldsymbol{\alpha}^*| \leq \max_{\boldsymbol{\alpha} \in B(\tilde{\boldsymbol{\beta}}, \tilde{\boldsymbol{\alpha}}) \cap H} |X_{:j}^\top \boldsymbol{\alpha}| = |X_{:j}^\top \tilde{\boldsymbol{\alpha}}| + r(\tilde{\boldsymbol{\beta}}, \tilde{\boldsymbol{\alpha}}) \|X_{:j} - \Pi_{\mathbf{1}}(X_{:j})\|_2, \tag{15}$$

where $\Pi_{\boldsymbol{u}}(\boldsymbol{v})$ is defined in Lemma 2.

Proof. We prove this via the method of Lagrange multiplier, that is,

$$\max_{\boldsymbol{\alpha}\in B(\tilde{\boldsymbol{\beta}},\tilde{\boldsymbol{\alpha}})\cap H} X_{:j}^\top \boldsymbol{\alpha} = \max_{\boldsymbol{\alpha}\in\mathbb{R}^n,\xi_1\in\mathbb{R},\xi_2\in\mathbb{R}} \mathcal{L}(\boldsymbol{\alpha},\xi_1,\xi_2),$$

$$\text{where} \qquad \mathcal{L}(\boldsymbol{\alpha},\xi_1,\xi_2) = X_{:j}^\top \boldsymbol{\alpha} - \xi_1\left(\|\boldsymbol{\alpha}-\tilde{\boldsymbol{\alpha}}\|_2^2 - r(\tilde{\boldsymbol{\beta}},\tilde{\boldsymbol{\alpha}})^2\right) - \xi_2 \boldsymbol{\alpha}^\top \mathbf{1}. \tag{16}$$

Let $(\boldsymbol{\alpha}^\#,\xi_1^\#,\xi_2^\#) := \arg\max_{\boldsymbol{\alpha}\in\mathbb{R}^n,\xi_1\in\mathbb{R},\xi_2\in\mathbb{R}} \mathcal{L}(\boldsymbol{\alpha},\xi_1,\xi_2)$ be the optimal solution of the latter maximization. Then it is known to satisfy the following conditions (*Karush-Kuhn-Tucker condition*):

$$\nabla L(\boldsymbol{\alpha},\xi_1,\xi_2)|_{\boldsymbol{\alpha}^\#,\xi_1^\#,\xi_2^\#} = \mathbf{0} \tag{17}$$

$$\xi_1^\# \geq 0 \tag{18}$$

$$\|\boldsymbol{\alpha}^\# - \tilde{\boldsymbol{\alpha}}\|_2^2 - r(\tilde{\boldsymbol{\beta}},\tilde{\boldsymbol{\alpha}})^2 \leq 0 \tag{19}$$

$$\xi_1^\#(\|\boldsymbol{\alpha}^\# - \tilde{\boldsymbol{\alpha}}\|_2^2 - r(\tilde{\boldsymbol{\beta}},\tilde{\boldsymbol{\alpha}})^2) = 0 \tag{20}$$

$$\xi_2^\# \geq 0 \tag{21}$$

$$\boldsymbol{\alpha}^{\#\top}\mathbf{1} = 0 \tag{22}$$

In this setup, it is clear that the maximization of $\mathcal{L}$ must be infinite if $\xi_1 = 0$. So, assuming $\xi_1^\# \neq 0$ in (18), (19) and (20), we have

$$\|\boldsymbol{\alpha}^\# - \tilde{\boldsymbol{\alpha}}\|_2^2 - r(\tilde{\boldsymbol{\beta}},\tilde{\boldsymbol{\alpha}})^2 = 0. \tag{23}$$

In addition, (17) is computed as follows:

$$\begin{aligned}
&\nabla L(\boldsymbol{\alpha},\xi_1,\xi_2)|_{\boldsymbol{\alpha}^\#,\xi_1^\#,\xi_2^\#} = X_{:j} - 2\xi_1^\#(\boldsymbol{\alpha}^\# - \tilde{\boldsymbol{\alpha}}) - \xi_2^\#\mathbf{1} = \mathbf{0} \qquad (24)\\
&X_{:j}^\top\mathbf{1} - 2\xi_1^\#(\boldsymbol{\alpha}^\# - \tilde{\boldsymbol{\alpha}})^\top\mathbf{1} - \xi_2^\#\|\mathbf{1}\|_2^2 = 0\\
&X_{:j}^\top\mathbf{1} = \xi_2^\#\|\mathbf{1}\|_2^2 \qquad (\because \boldsymbol{\alpha}^{\#\top}\mathbf{1} = \tilde{\boldsymbol{\alpha}}^\top\mathbf{1} = 0)\\
&\therefore \xi_2^\# = \frac{X_{:j}^\top\mathbf{1}}{\|\mathbf{1}\|_2^2}.
\end{aligned}$$

Moreover, substituting $\xi_2^\#$ in (24) we have

$$\begin{aligned}
&X_{:j} - 2\xi_1^\#(\boldsymbol{\alpha}^\# - \tilde{\boldsymbol{\alpha}}) - \frac{X_{:j}^\top\mathbf{1}}{\|\mathbf{1}\|_2^2}\mathbf{1} = X_{:j} - 2\xi_1^\#(\boldsymbol{\alpha}^\# - \tilde{\boldsymbol{\alpha}}) - \Pi_{\mathbf{1}}(X_{:j}) = \mathbf{0} \qquad (25)\\
&4(\xi_1^\#)^2\|\boldsymbol{\alpha}^\# - \tilde{\boldsymbol{\alpha}}\|_2^2 = 4(\xi_1^\#)^2 r(\tilde{\boldsymbol{\beta}},\tilde{\boldsymbol{\alpha}})^2 = \|X_{:j} - \Pi_{\mathbf{1}}(X_{:j})\|_2^2 \qquad (\because \text{(23)})\\
&\therefore \xi_1^\# = \frac{\|X_{:j} - \Pi_{\mathbf{1}}(X_{:j})\|_2}{2r(\tilde{\boldsymbol{\beta}},\tilde{\boldsymbol{\alpha}})}.
\end{aligned}$$

As a result, substituting $\xi_1^\#$ in (25) we have

$$\boldsymbol{\alpha}^\# = \tilde{\boldsymbol{\alpha}} + r(\tilde{\boldsymbol{\beta}},\tilde{\boldsymbol{\alpha}})\frac{X_{:j} - \Pi_{\mathbf{1}}(X_{:j})}{\|X_{:j} - \Pi_{\mathbf{1}}(X_{:j})\|_2},$$

and

$$\begin{aligned}
&\max_{\boldsymbol{\alpha}\in B(\tilde{\boldsymbol{\beta}},\tilde{\boldsymbol{\alpha}})\cap H} X_{:j}^\top\boldsymbol{\alpha} = L(\boldsymbol{\alpha}^\#,\xi_1^\#,\xi_2^\#) = X_{:j}^\top\boldsymbol{\alpha}^\#\\
&= [X_{:j} - \Pi_{\mathbf{1}}(X_{:j})]^\top\boldsymbol{\alpha}^\# \qquad (\because [\Pi_{\mathbf{1}}(X_{:j})]^\top\boldsymbol{\alpha}^\# = 0 \text{ by (22)})\\
&= [X_{:j} - \Pi_{\mathbf{1}}(X_{:j})]^\top\tilde{\boldsymbol{\alpha}} + r(\tilde{\boldsymbol{\beta}},\tilde{\boldsymbol{\alpha}})\|X_{:j} - \Pi_{\mathbf{1}}(X_{:j})\|_2\\
&= X_{:j}^\top\tilde{\boldsymbol{\alpha}} + r(\tilde{\boldsymbol{\beta}},\tilde{\boldsymbol{\alpha}})\|X_{:j} - \Pi_{\mathbf{1}}(X_{:j})\|_2. \qquad (26)
\end{aligned}$$

This concludes $\max_{\boldsymbol{\alpha}\in B(\tilde{\boldsymbol{\beta}},\tilde{\boldsymbol{\alpha}})\cap H} X_{:j}^\top \boldsymbol{\alpha} = X_{:j}^\top \tilde{\boldsymbol{\alpha}} + r(\tilde{\boldsymbol{\beta}},\tilde{\boldsymbol{\alpha}})\|X_{:j} - \Pi_{\mathbf{1}}(X_{:j})\|_2$. The result consequently proves that

$$
\begin{aligned}
&\max_{\boldsymbol{\alpha}\in B(\tilde{\boldsymbol{\beta}},\tilde{\boldsymbol{\alpha}})\cap H} (-X_{:j})^\top \boldsymbol{\alpha} \\
&= -X_{:j}^\top \tilde{\boldsymbol{\alpha}} + r(\tilde{\boldsymbol{\beta}},\tilde{\boldsymbol{\alpha}})\| - X_{:j} - \Pi_{\mathbf{1}}(-X_{:j})\|_2 \quad (\because\ X_{:j} \leftarrow (-X_{:j}) \text{ in (26)}) \\
&= -X_{:j}^\top \tilde{\boldsymbol{\alpha}} + r(\tilde{\boldsymbol{\beta}},\tilde{\boldsymbol{\alpha}})\|X_{:j} - \Pi_{\mathbf{1}}(X_{:j})\|_2.
\end{aligned}
$$

Combining them, we have $\max_{\boldsymbol{\alpha}\in B(\tilde{\boldsymbol{\beta}},\tilde{\boldsymbol{\alpha}})\cap H} |X_{:j}^\top \boldsymbol{\alpha}| = |X_{:j}^\top \tilde{\boldsymbol{\alpha}}| + r(\tilde{\boldsymbol{\beta}},\tilde{\boldsymbol{\alpha}})\|X_{:j} - \Pi_{\mathbf{1}}(X_{:j})\|_2$. ■

Finally, Lemma 2 is proved as follows:

Proof of Lemma 2. Suppose that $u_j(\tilde{\boldsymbol{\beta}},\tilde{\boldsymbol{\alpha}}) < \lambda_1$. Then, by Lemma 8 we have

$$
\begin{aligned}
\lambda > u_j(\tilde{\boldsymbol{\beta}},\tilde{\boldsymbol{\alpha}}) &:= |X_{:j}^\top \tilde{\boldsymbol{\alpha}}| + r(\tilde{\boldsymbol{\beta}},\tilde{\boldsymbol{\alpha}})\|X_{:j} - \Pi_{\mathbf{1}}(X_{:j})\|_2 \\
&\geq \max_{\boldsymbol{\alpha}\in B(\tilde{\boldsymbol{\beta}},\tilde{\boldsymbol{\alpha}})\cap H} |X_{:j}^\top \boldsymbol{\alpha}| \geq |X_{:j}^\top \boldsymbol{\alpha}^*|.
\end{aligned}
$$

By equation (8), $\beta_j^* = 0$ must hold. ■

Supplemental note S2: Proof of Theorem 4

Proof. First, we prove that the SPP-score in (11) is greater than or equal to the safe screening score in (9), i.e., $v_j(\tilde{\boldsymbol{\beta}},\tilde{\boldsymbol{\alpha}}) \geq u_j(\tilde{\boldsymbol{\beta}},\tilde{\boldsymbol{\alpha}})$. This can be shown as

$$
\begin{aligned}
v_j(\tilde{\boldsymbol{\beta}},\tilde{\boldsymbol{\alpha}}) - u_j(\tilde{\boldsymbol{\beta}},\tilde{\boldsymbol{\alpha}}) &= \max\left\{\sum_{i:\tilde{\alpha}_i>0} x_{ij}\tilde{\alpha}_i, -\sum_{i:\tilde{\alpha}_i<0} x_{ij}\tilde{\alpha}_i\right\} - |X_{:j}^\top \tilde{\boldsymbol{\alpha}}| + r(\tilde{\boldsymbol{\beta}},\tilde{\boldsymbol{\alpha}})\left(\|X_{:j}\|_2 - \|X_{:j} - \Pi_{\mathbf{1}}(X_{:j})\|_2\right) \\
&\geq \max\left\{\sum_{i:\tilde{\alpha}_i>0} x_{ij}\tilde{\alpha}_i, -\sum_{i:\tilde{\alpha}_i<0} x_{ij}\tilde{\alpha}_i\right\} - \max\left\{\sum_{i\in[n]} x_{ij}\alpha_i, -\sum_{i\in[n]} x_{ij}\alpha_i\right\} \\
&\geq 0.
\end{aligned}
$$

Therefore, using Lemma 2, we have

$$
v_j(\tilde{\boldsymbol{\beta}},\tilde{\boldsymbol{\alpha}}) < \lambda \Rightarrow u_j(\tilde{\boldsymbol{\beta}},\tilde{\boldsymbol{\alpha}}) < \lambda \Rightarrow \beta_j^* = 0.
$$

Next, we prove that, for pair of pattern p_j and p_k such that $p_k \sqsubset p_j$, the SPP score of p_j is grater than or equal to that of p_k, i.e., $v_j(\tilde{\boldsymbol{\beta}},\tilde{\boldsymbol{\alpha}}) \geq v_k(\tilde{\boldsymbol{\beta}},\tilde{\boldsymbol{\alpha}})$. To show this, we prove that each of the two terms of the SPP score satisfies the intended inequality relationship, thereby showing that the SPP score as a whole also satisfies the inequality relationship. The inequality relationship for the first term of the SPP score is shown as follows. From Lemma 3, it is clear that

$$
\sum_{i:\tilde{\alpha}_i>0} x_{ij}\tilde{\alpha}_i \geq \sum_{i:\tilde{\alpha}_i>0} x_{ik}\tilde{\alpha}_i.
$$

Therefore, we have

$$
\max\left\{\sum_{i:\tilde{\alpha}_i>0} x_{ij}\tilde{\alpha}_i, -\sum_{i:\tilde{\alpha}_i<0} x_{ij}\tilde{\alpha}_i\right\} \geq \max\left\{\sum_{i:\tilde{\alpha}_i>0} x_{ik}\tilde{\alpha}_i, -\sum_{i:\tilde{\alpha}_i<0} x_{ik}\tilde{\alpha}_i\right\}. \quad (27)
$$

The inequality relationship for the second term of the SPP score is easily shown by noting that

$$\|X_{:j}\|_2 \geq \|X_{:k}\|_2.$$

This means that

$$v_j(\tilde{\boldsymbol{\beta}}, \tilde{\boldsymbol{\alpha}}) < \lambda \;\Rightarrow\; v_k(\tilde{\boldsymbol{\beta}}, \tilde{\boldsymbol{\alpha}}) < \lambda \;\Rightarrow\; \beta_k^* = 0 \;\; \forall k \in [d] \text{ s.t. } p_k \sqsubset p_j.$$

■

Supplemental note S3: Proof of Theorem 5

In order to prove the theorem, we first prove the following lemma.

Lemma 9 (Union of two hyperspheres)**.** *Suppose that two hyperspheres in $\mathbb{R}^n$, denoted by $S_1 = \{\boldsymbol{v} \in \mathbb{R}^n \mid \|\boldsymbol{v} - \boldsymbol{c}_1\|_2 = r_1\}$ and $S_2 = \{\boldsymbol{v} \in \mathbb{R}^n \mid \|\boldsymbol{v} - \boldsymbol{c}_2\|_2 = r_2\}$, satisfies $S_1 \cap S_2 \not\equiv \emptyset$ and $S_1 \not\equiv S_2$, that is,*

$$\delta := \|\boldsymbol{c}_1 - \boldsymbol{c}_2\|_2 > 0, \tag{28}$$
$$r_1 + r_2 \geq \delta, \tag{29}$$
$$|r_1 - r_2| \leq \delta. \tag{30}$$

Then, the intersection of them $S_1 \cap S_2$ is identical to the intersection $S' \cap H'$ of the following hypersphere S' and hyperplane H':

$$S' = \{\boldsymbol{v} \in \mathbb{R}^n \mid \|\boldsymbol{v} - \boldsymbol{c}'\|_2 = r'\},$$
$$H' = \{\boldsymbol{v} \in \mathbb{R}^n \mid (\boldsymbol{v} - \boldsymbol{c}')^\top(\boldsymbol{c}_1 - \boldsymbol{c}_2) = 0\},$$

where $\boldsymbol{c}'$ (center of S') and r' (radius of S') are defined as follows:

$$\boldsymbol{c}' = t\boldsymbol{c}_1 + (1-t)\boldsymbol{c}_2,$$
$$r' = \sqrt{r_2^2 - t^2\delta^2},$$
$$t = \frac{1}{2}\left(1 + \frac{r_2^2 - r_1^2}{\delta^2}\right).$$

Proof of Lemma 9. Let $E : \mathbb{R}^n \to \mathbb{R}^n$ be an distance-preserving mapping such that

$$E\boldsymbol{c}_1 = \boldsymbol{0},$$
$$E\boldsymbol{c}_2 = [\delta, \underbrace{0, \dots, 0}_{n-1}]^\top,$$

where $\delta = \|\boldsymbol{c}_1 - \boldsymbol{c}_2\|_2$. Note that such a mapping can be obtained as follows:

- Let $E\boldsymbol{v} := \Theta(\boldsymbol{v} - \boldsymbol{c}_1)$ ($\Theta \in \mathbb{R}^{n\times n}$).
- Set $\Theta_{1:} = \frac{1}{\delta^2}(\boldsymbol{c}_1 - \boldsymbol{c}_2)$.
- Set other rows of Θ so that Θ is an orthogonal matrix. This can be done by Gram-Schmidt algorithm.

Let $\boldsymbol{v} \in S_1 \cap S_2$, and $\boldsymbol{v}'$ be

$$E\boldsymbol{v} = \boldsymbol{v}' = [v'_1, \dots, v'_n]^\top.$$

Then, since E is distance-preserving, we have

$$\|E\boldsymbol{v} - E\boldsymbol{c}_1\|_2^2 = r_1^2 \iff \sum_{i=1}^{n} v_i'^2 = r_1^2, \tag{31}$$

$$\|E\boldsymbol{v} - E\boldsymbol{c}_2\|_2^2 = r_2^2 \iff \sum_{i=2}^{n} v_i'^2 + (v'_1 - \delta)^2 = r_2^2. \tag{32}$$

Taking the difference between equations (31) and (32), we have

$$v'_1 = \frac{r_1^2 - r_2^2 + \delta^2}{2\delta}, \tag{33}$$

$$\sum_{i=2}^{n} v_i'^2 = r_2^2 - (v'_1 - \delta)^2 = r_2^2 - \left(\frac{r_1^2 - r_2^2 - \delta^2}{2\delta}\right)^2 = r_2^2 - t^2\delta^2. \tag{34}$$

Note that the value (34) is nonnegative because

$$\begin{aligned} r_2^2 - \left(\frac{r_1^2 - r_2^2 - \delta^2}{2\delta}\right)^2 &= \frac{1}{2\delta}(2\delta r_2 + r_1^2 - r_2^2 - \delta^2)(2\delta r_2 - r_1^2 + r_2^2 + \delta^2) \\ &= \frac{1}{2\delta}[r_1^2 - (r_2 - \delta)^2][(r_2 + \delta)^2 - r_1^2] \\ &= \frac{1}{2\delta}\underbrace{(r_1 + r_2 - \delta)}_{\geq 0 \because (29)}\underbrace{(r_1 - r_2 + \delta)}_{\geq 0 \because (30)}\underbrace{(r_1 + r_2 + \delta)}_{>0 \text{ clearly}}\underbrace{(-r_1 + r_2 + \delta)}_{\geq 0 \because (30)}. \end{aligned}$$

In summary, equation (33) implies that v'_1 of $\boldsymbol{v}'$ is constant. In addition, equation (34) implies that $[v'_2, \dots, v'_n]$ is on a hypersphere whose center is $[v'_2, \dots, v'_n]^\top = [\underbrace{0, \dots, 0}_{n-1}]^\top$. So, if we take a hypersphere whose center is $E\boldsymbol{c}' = [v'_1, \underbrace{0, \dots, 0}_{n-1}]^\top$ and whose radius is $r' = \sqrt{r_2^2 - t^2\delta^2}$, then the intersection of it and the hyperplane $v'_1 = \frac{r_1^2 - r_2^2 + \delta^2}{2\delta}$ composes $\boldsymbol{v}'$.

Finally we derive the center of the hypersphere in the original space $\boldsymbol{c}'$. (Note that r' is the same between in the original space and the space after applying E, since E is distance-preserving.) Again, in the space after applying E,

$$E\boldsymbol{c}_1 = [0, \underbrace{0, \dots, 0}_{n-1}]^\top, \quad E\boldsymbol{c}' = [v'_1, \underbrace{0, \dots, 0}_{n-1}]^\top, \quad E\boldsymbol{c}_2 = [\delta, \underbrace{0, \dots, 0}_{n-1}]^\top.$$

Since E is distance-preserving, $\boldsymbol{c}'$ in the original space can be computed as

$$\begin{aligned} \boldsymbol{c}' &= \boldsymbol{c}_1 + \frac{v'_1}{\delta}(\boldsymbol{c}_2 - \boldsymbol{c}_1) \\ &= \boldsymbol{c}_1 + \frac{r_1^2 - r_2^2 + \delta^2}{2\delta^2}(\boldsymbol{c}_2 - \boldsymbol{c}_1) \\ &= \frac{\delta^2 - r_1^2 + r_2^2}{2\delta^2}\boldsymbol{c}_1 + \left(1 - \frac{\delta^2 - r_1^2 + r_2^2}{2\delta^2}\right)\boldsymbol{c}_2 = t\boldsymbol{c}_1 + (1 - t)\boldsymbol{c}_2. \end{aligned}$$

This derives $\boldsymbol{c}'$ and t in the lemma. ■

Proof of Theorem 5. In order to prove

$$\max_{\boldsymbol{\alpha}\in B_1\cap B_2\cap H} |X_{:j}^\top \boldsymbol{\alpha}| = \max\{u_j^+, u_j^-\},$$

first we note that

$$\max_{\boldsymbol{\alpha}} |X_{:j}^\top \boldsymbol{\alpha}| = \max\{\max_{\boldsymbol{\alpha}} X_{:j}^\top \boldsymbol{\alpha}, \max_{\boldsymbol{\alpha}} (-X_{:j})^\top \boldsymbol{\alpha}\}.$$

The second expression can be obtained by just replacing $X_{:j}$ with $-X_{:j}$ in the first expression. So we discuss only the first expression, that is,

$$\max_{\boldsymbol{\alpha}\in B_1\cap B_2\cap H} X_{:j}^\top \boldsymbol{\alpha}. \tag{35}$$

The Lagrangian function of (35) is defined as

$$\mathcal{L}(\boldsymbol{\alpha}, \xi_1, \xi_2, \xi_3) = X_{:j}^\top \boldsymbol{\alpha} - \xi_1(\|\boldsymbol{\alpha} - \tilde{\boldsymbol{\alpha}}^{(1)}\|_2^2 - r(R_1)^2) - \xi_2(\|\boldsymbol{\alpha} - \tilde{\boldsymbol{\alpha}}^{(2)}\|_2^2 - r(R_2)^2) - \xi_3 \boldsymbol{\alpha}^\top \mathbf{1}.$$

Then, the optimal solution of (35), denoted by $\tilde{\boldsymbol{\alpha}}^*$, must satisfy the following conditions:

$$\nabla_{\boldsymbol{\alpha}} \mathcal{L}|_{\boldsymbol{\alpha}=\tilde{\boldsymbol{\alpha}}^*} = \mathbf{0}, \tag{36}$$

$$\xi_1 \geq 0, \tag{37}$$

$$\|\tilde{\boldsymbol{\alpha}}^* - \tilde{\boldsymbol{\alpha}}^{(1)}\|_2^2 - r(R_1)^2 \leq 0, \tag{38}$$

$$\xi_1(\|\tilde{\boldsymbol{\alpha}}^* - \tilde{\boldsymbol{\alpha}}^{(1)}\|_2^2 - r(R_1)^2) = 0, \tag{39}$$

$$\xi_2 \geq 0, \tag{40}$$

$$\|\tilde{\boldsymbol{\alpha}}^* - \tilde{\boldsymbol{\alpha}}^{(2)}\|_2^2 - r(R_2)^2 \leq 0, \tag{41}$$

$$\xi_2(\|\tilde{\boldsymbol{\alpha}}^* - \tilde{\boldsymbol{\alpha}}^{(2)}\|_2^2 - r(R_2)^2) = 0, \tag{42}$$

$$\boldsymbol{\alpha}^\top \mathbf{1} = 0. \tag{43}$$

Note that, if $\xi_1 = \xi_2 = 0$, (i.e., neither (38) nor (41) are active), then $\max_{\boldsymbol{\alpha}} \mathcal{L}$ cannot be finite. So we can assume that $(\xi_1, \xi_2) \neq (0, 0)$. From (36), we have

$$\nabla_{\boldsymbol{\alpha}} \mathcal{L} = X_{:j} - 2\xi_1(\boldsymbol{\alpha} - \tilde{\boldsymbol{\alpha}}^{(1)}) - 2\xi_2(\boldsymbol{\alpha} - \tilde{\boldsymbol{\alpha}}^{(2)}) - \xi_3 \mathbf{1}.$$

Since $\xi_1 + \xi_2 \neq 0$, we have

$$\tilde{\boldsymbol{\alpha}}^* = \frac{1}{2(\xi_1 + \xi_2)} \left(X_{:j} + 2\xi_1 \tilde{\boldsymbol{\alpha}}^{(1)} + 2\xi_2 \tilde{\boldsymbol{\alpha}}^{(2)} - \xi_3 \mathbf{1}\right).$$

Moreover, since $\mathbf{1}^\top \tilde{\boldsymbol{\alpha}}^{(1)} = \mathbf{1}^\top \tilde{\boldsymbol{\alpha}}^{(2)} = 0$ from (43), we have

$$\frac{1}{2(\xi_1 + \xi_2)} \left(X_{:j}^\top \mathbf{1} - \xi_3 \mathbf{1}^\top \mathbf{1}\right) = 0$$

$$\therefore \xi_3 = \frac{X_{:j}^\top \mathbf{1}}{\mathbf{1}^\top \mathbf{1}}.$$

Here we calculate the solution $\tilde{\boldsymbol{\alpha}}^*$ based on the values of ξ_1 and ξ_2. First, if $\xi_1 \neq 0$ and $\xi_2 = 0$, (i.e., (38) is active but not (41)), then we have $\|\boldsymbol{\alpha} - \tilde{\boldsymbol{\alpha}}^{(1)}\|_2^2 = r(R_1)^2$ and

$$\frac{1}{4\xi_1^2} \|X_{:j} - \Pi_{\mathbf{1}}(X_{:j})\|_2^2 = r(R_1)^2$$

$$\therefore \xi_1 = \frac{\|X_{:j} - \Pi_{\mathbf{1}}(X_{:j})\|_2}{r(R_1)},$$

then

$$\tilde{\boldsymbol{\alpha}}^* = \frac{r(R_1)}{\|X_{:j} - \Pi_{\mathbf{1}}(X_{:j})\|_2} (X_{:j} - \Pi_{\mathbf{1}}(X_{:j})) + \tilde{\boldsymbol{\alpha}}^{(1)},$$

and the maximized result is calculated as

$$\begin{aligned}\mathcal{L}(\tilde{\boldsymbol{\alpha}}^*, \xi_1, \xi_2, \xi_3) &= X_{:j}^\top \tilde{\boldsymbol{\alpha}}^* \\ &= (X_{:j} - \Pi_{\mathbf{1}}(X_{:j}))^\top \tilde{\boldsymbol{\alpha}}^* \\ &= X_{:j}^\top \tilde{\boldsymbol{\alpha}}^{(1)} + r(R_1)\|X_{:j} - \Pi_{\mathbf{1}}(X_{:j})\|_2. \end{aligned} \tag{44}$$

In this case $\tilde{\boldsymbol{\alpha}}^* \in B_2$ must hold, that is, $\|\tilde{\boldsymbol{\alpha}}^* - \tilde{\boldsymbol{\alpha}}^{(2)}\|_2^2 \le r(R_2)^2$. So we have

$$\frac{X_{:j}^\top \boldsymbol{\delta}}{\|X_{:j} - \Pi_{\mathbf{1}}(X_{:j})\|_2} \le \frac{r(R_2)^2 - r(R_1)^2 - \|\boldsymbol{\delta}\|_2^2}{2r(R_1)}.$$

If $\xi_1 = 0$ and $\xi_2 \neq 0$, the calculation can be done similarly: we can conclude that

$$\tilde{\boldsymbol{\alpha}}^* = \frac{r(R_2)}{\|X_{:j} - \Pi_{\mathbf{1}}(X_{:j})\|_2} (X_{:j} - \Pi_{\mathbf{1}}(X_{:j})) + \tilde{\boldsymbol{\alpha}}^{(2)}$$

and

$$\mathcal{L}(\tilde{\boldsymbol{\alpha}}^*, \xi_1, \xi_2, \xi_3) = X_{:j}^\top \tilde{\boldsymbol{\alpha}}^{(2)} + r(R_2)\|X_{:j} - \Pi_{\mathbf{1}}(X_{:j})\|_2. \tag{45}$$

Since $\tilde{\boldsymbol{\alpha}}^* \in B_1$, we also have

$$\frac{X_{:j}^\top \boldsymbol{\delta}}{\|X_{:j} - \Pi_{\mathbf{1}}(X_{:j})\|_2} \ge \frac{r(R_2)^2 - r(R_1)^2 + \|\boldsymbol{\delta}\|_2^2}{2r(R_2)}.$$

Finally we show the case of $\xi_1 \neq 0$ and $\xi_2 \neq 0$. In this case, since $\|\boldsymbol{\alpha} - \tilde{\boldsymbol{\alpha}}^{(1)}\|_2^2 = r(R_1)^2$ and $\|\boldsymbol{\alpha} - \tilde{\boldsymbol{\alpha}}^{(2)}\|_2^2 = r(R_2)^2$, the constraint can be represented as an intersection of two hyperspheres in $\mathbb{R}^n$. So we replace them with Lemma 9. Let S_1 and S_2 be the surfaces of B_1 and B_2, respectively. Then the problem is rewritten as:

$$\max_{\boldsymbol{\alpha} \in S_1 \cap S_2 \cap H} X_{:j}^\top \boldsymbol{\alpha} = \max_{\boldsymbol{\alpha} \in S' \cap H' \cap H} X_{:j}^\top \boldsymbol{\alpha},$$

where

$$\begin{aligned} S' &= \{\boldsymbol{v} \in \mathbb{R}^n \mid \|\boldsymbol{v} - \tilde{\boldsymbol{\alpha}}'\| < r'\}, \\ H' &= \{\boldsymbol{v} \in \mathbb{R}^n \mid (\boldsymbol{v} - \tilde{\boldsymbol{\alpha}}')^\top \boldsymbol{\delta} = 0\}, \end{aligned}$$

and $\tilde{\boldsymbol{\alpha}}', r', \boldsymbol{\delta}$ are the ones defined in Theorem 5. Its Lagrangian function $\mathcal{L}'$ is defined as

$$\mathcal{L}'(\boldsymbol{\alpha}, \xi_1', \xi_2', \xi_3') = X_{:j}^\top \boldsymbol{\alpha} - \xi_1'(\|\boldsymbol{\alpha} - \tilde{\boldsymbol{\alpha}}'\|_2^2 - r'^2) - \xi_2'(\boldsymbol{\alpha} - \tilde{\boldsymbol{\alpha}})^\top \boldsymbol{\delta} - \xi_3' \boldsymbol{\alpha}^\top \mathbf{1},$$

with the optimality conditions

$$\nabla_{\boldsymbol{\alpha}} \mathcal{L}|_{\boldsymbol{\alpha} = \tilde{\boldsymbol{\alpha}}^*} = \mathbf{0}, \tag{46}$$

$$\|\tilde{\boldsymbol{\alpha}}^* - \tilde{\boldsymbol{\alpha}}'\|_2^2 - r' = 0, \tag{47}$$

$$(\tilde{\boldsymbol{\alpha}}^* - \tilde{\boldsymbol{\alpha}}')^\top \boldsymbol{\delta} = 0, \tag{48}$$

$$\tilde{\boldsymbol{\alpha}}^{*\top} \mathbf{1} = 0. \tag{49}$$

Noticing that $\xi_1 \neq 0$ (otherwise $\max_{\boldsymbol{\alpha}} \mathcal{L}'$ is not bounded), from (46) we have

$$\nabla \mathcal{L} = X_{:j} - 2\xi_1'(\boldsymbol{\alpha} - \tilde{\boldsymbol{\alpha}}') - \xi_2' \boldsymbol{\delta} - \xi_3' \mathbf{1}$$

and

$$\tilde{\boldsymbol{\alpha}}^* = \tilde{\boldsymbol{\alpha}}' + \frac{1}{2\xi_1'}\left(\xi_2'\boldsymbol{\delta} + X_{:j} - \xi_3'\mathbf{1}\right).$$

Since $\mathbf{1}^\top\boldsymbol{\delta} = 0$, from (49) we have

$$\xi_3' = \frac{X_{:j}^\top\mathbf{1}}{\|\mathbf{1}\|_2^2}$$

and from (48) we have

$$\xi_2' = -\frac{X_{:j}^\top\boldsymbol{\delta}}{\|\boldsymbol{\delta}\|_2^2}.$$

Then, from (47) we have

$$\xi_1' = \frac{\|X_{:j} - \Pi_{\mathbf{1}}(X_{:j}) - \Pi_{\boldsymbol{\delta}}(X_{:j})\|_2^2}{2r'}.$$

Since $\boldsymbol{\alpha} \in H$ and $\boldsymbol{\alpha} \in H'$, we have $\boldsymbol{\alpha}^\top\Pi_{\mathbf{1}}(X_{:j}) = 0$ and $\boldsymbol{\alpha}^\top\Pi_{\boldsymbol{\delta}}(X_{:j}) = \tilde{\boldsymbol{\alpha}}^\top\Pi_{\boldsymbol{\delta}}(X_{:j})$, respectively. Thus we can conclude that

$$\begin{aligned}
\mathcal{L}^*(\tilde{\boldsymbol{\alpha}}^*, \xi_1', \xi_2', \xi_3') &= X_{:j}^\top\tilde{\boldsymbol{\alpha}}^* \\
&= (X_{:j} - \Pi_{\mathbf{1}}(X_{:j}) - \Pi_{\boldsymbol{\delta}}(X_{:j}))^\top\tilde{\boldsymbol{\alpha}}^* + \tilde{\boldsymbol{\alpha}}'^\top\Pi_{\mathbf{1}}(X_{:j}) \\
&= X_{:j}^\top\tilde{\boldsymbol{\alpha}}' + r'\|X_{:j} - \Pi_{\mathbf{1}}(X_{:j}) - \Pi_{\boldsymbol{\delta}}(X_{:j})\|_2^2.
\end{aligned}$$

■

Supplemental note S4: Algorithms

We present Algorithms 1 to 4 described in this paper.

Algorithm 1 Safe Pattern Pruning

Input: $X, \boldsymbol{y}, \lambda, \kappa, R = (\tilde{\boldsymbol{\beta}}, \tilde{\boldsymbol{\alpha}})$
Output: $\mathcal{A}$
  $\mathcal{A} \leftarrow \emptyset$
  $\mathcal{P} \leftarrow \{\emptyset\}$
  while $\mathcal{P} \neq \emptyset$ **do**
    Pop from the top of $\mathcal{P}$ as p
    Enumerate expanded patterns $\mathcal{P}'$ from p
    for $p_j \in \mathcal{P}'$ **do**
      if $v_j(R) < \lambda$ **then**
        comtinue
      end if
      if $u_j(R) \geq \lambda$ **then**
        $\mathcal{A} \leftarrow \mathcal{A} \cup \{j\}$
      end if
      Push p' into the top of $\mathcal{P}$
    end for
  end while

Algorithm 2 Pathwise optimization with SPP

Input: $X, \boldsymbol{y}, \{(\lambda^{(k)}, \kappa^{(k)})\}_{k \in [K]}, \epsilon$
Output: $\{\boldsymbol{\beta}^{*(k)}\}_{k \in [K]}$
  $\boldsymbol{\beta} \leftarrow \mathbf{0}$
  for $k \in [K]$ **do**
    $\lambda \leftarrow \lambda^{(k)}$
    $\kappa \leftarrow \kappa^{(k)}$
    Compute $\boldsymbol{\alpha}$ from $\boldsymbol{\beta}$ by the dual scaling
    $R \leftarrow (\boldsymbol{\beta}, \boldsymbol{\alpha})$
    $\mathcal{A} \leftarrow \mathrm{SafePatternPruning}(X, \boldsymbol{y}, \lambda, \kappa, R)$
    while $true$ **do**
      Update $\boldsymbol{\beta}$ using the sub-gradient of P
      Copute $\boldsymbol{\alpha}$ from $\boldsymbol{\beta}$ by hte dual scaling
      $R \leftarrow (\boldsymbol{\beta}, \boldsymbol{\alpha})$
      if $G(R) < \epsilon$ **then**
        $\boldsymbol{\beta}^{*(k)} \leftarrow \boldsymbol{\beta}$
        break
      end if
      Remove inactive patterns from $\mathcal{A}$ using safe screening
    end while
  end for

Algorithm 3 Pathwise optimization with multi-reference SPP

Input: $X, \boldsymbol{y}, \{\lambda^{(k)}\}_{k\in[K]}, \{\kappa^{(k')}\}_{k'\in[K']}, \epsilon, M$

Output: $\{R^{(k,k')}\}_{(k,k')\in[K]\times[K']}$

for $k \in [K]$ **do**
  for $k' \in [K']$ **do**
    $\lambda \leftarrow \lambda^{(k)}$
    $\kappa \leftarrow \kappa^{(k')}$
    $\mathcal{R} \leftarrow \emptyset$
    if $k > 1$ **then**
      $\mathcal{R} \leftarrow \mathcal{R} \cup \{R^{(k-1,k')}\}$
    end if
    if $k' > 1$ **then**
      $\mathcal{R} \leftarrow \mathcal{R} \cup \{R^{(k,k'-1)}\}$
    end if
    if $\mathcal{R} = \emptyset$ **then**
      $\mathcal{R} \leftarrow \mathcal{R} \cup \{(\mathbf{0}, \mathbf{0})\}$
    end if
    for $R \in \mathcal{R}$ **do**
      $\boldsymbol{\beta}, \boldsymbol{\alpha} \leftarrow R$
      Update $\boldsymbol{\alpha}$ from $\boldsymbol{\beta}$ by dual scaling
    end for
    $\mathcal{A} \leftarrow \mathrm{MultiSafePatternPruning}(X, \boldsymbol{y}, \lambda, \kappa, \mathcal{R})$
    for $m \in \{1, 2, \dots\}$ **do**
      for $R \in \mathcal{R}$ **do**
        $\boldsymbol{\beta}, \boldsymbol{\alpha} \leftarrow R$
        Update $\boldsymbol{\beta}$ of R using the sub-gradient of P
        Update $\boldsymbol{\alpha}$ from $\boldsymbol{\beta}$ using the dual scaling
        $R \leftarrow (\boldsymbol{\beta}, \boldsymbol{\alpha})$
      end for
      if $\min_{R\in\mathcal{R}} G(R) < \epsilon$ **then**
        $\boldsymbol{\beta}^{*(k,k')} \leftarrow \boldsymbol{\beta}$
        break
      end if
      Remove inactive patterns from $\mathcal{A}$ using multi safe screening
      if $m \geq M$ **then**
        $\mathcal{R} \leftarrow \{\mathrm{argmin}_{R\in\mathcal{R}} G(R)\}$
      end if
    end for
  end for
end for

Algorithm 4 Pathwise optimization in cross-validation with multi-reference SPP

Input: $X, \boldsymbol{y}, \{\mathcal{I}^{(k)}\}_{k\in[K]}, \{\lambda^{(k')}\}_{k'\in[K']}, \kappa, \epsilon, M$
Output: $\{R^{(k,k')}\}_{(k,k')\in[K]\times[K']}$
  for $k \in [K]$ **do**
    $X' \leftarrow (X^\top_{\mathcal{I}^{(k)}})^\top$
    $\boldsymbol{y}' \leftarrow \boldsymbol{y}_{\mathcal{I}^{(k)}}$
    for $k' \in [K']$ **do**
      $\lambda \leftarrow \lambda^{(k')}$
      $\mathcal{R} \leftarrow \emptyset$
      if $k > 1$ **then**
        $\mathcal{R} \leftarrow \mathcal{R} \cup \{R^{(1,k')}\}$
      end if
      if $k' > 1$ **then**
        $\mathcal{R} \leftarrow \mathcal{R} \cup \{R^{(k,k'-1)}\}$
      end if
      if $\mathcal{R} = \emptyset$ **then**
        $\mathcal{R} \leftarrow \mathcal{R} \cup \{(\mathbf{0}, \mathbf{0})\}$
      end if
      for $R \in \mathcal{R}$ **do**
        $\boldsymbol{\beta}, \boldsymbol{\alpha} \leftarrow R$
        Update $\boldsymbol{\alpha}$ from $\boldsymbol{\beta}$ by dual scaling
      end for
      $\mathcal{A} \leftarrow \mathrm{MultiSafePatternPruning}(X', \boldsymbol{y}', \lambda, \kappa, \mathcal{R})$
      for $m \in \{1, 2, \ldots\}$ **do**
        for $R \in \mathcal{R}$ **do**
          $\boldsymbol{\beta}, \boldsymbol{\alpha} \leftarrow R$
          Update $\boldsymbol{\beta}$ of R using the sub-gradient of P
          Update $\boldsymbol{\alpha}$ from $\boldsymbol{\beta}$ using the dual scaling
          $R \leftarrow (\boldsymbol{\beta}, \boldsymbol{\alpha})$
        end for
        if $\min_{R\in\mathcal{R}} G(R) < \epsilon$ **then**
          $\boldsymbol{\beta}^{*(k,k')} \leftarrow \boldsymbol{\beta}$
          break
        end if
        Remove inactive patterns from $\mathcal{A}$ using multi safe screening
        if $m \geq M$ **then**
          $\mathcal{R} \leftarrow \{\mathrm{argmin}_{R\in\mathcal{R}} G(R)\}$
        end if
      end for
    end for
  end for

References

1. Fercoq, O., Gramfort, A., and Salmon, J. Mind the duality gap: safer rules for the lasso. In: *International Conference on Machine Learning*. PMLR (2015):(333–342). doi:https://dl.acm.org/doi/10.5555/3045118.3045155.

2. Ndiaye, E., Fercoq, O., Gramfort, A., and Salmon, J. (2017). Gap safe screening rules for sparsity enforcing penalties. The Journal of Machine Learning Research *18*, 4671–4703. doi:https://dl.acm.org/doi/abs/10.5555/3122009.3208009.

3. Rockafellar, R. T., and Wets, R. J.-B. Variational analysis chap. §11.H “Dual Problems of Optimization”, §11.I “Lagrangian Functions”, §12.H “Strong Monotonicity and Strong Convexity”. Springer Science & Business Media (2009):.

4. Bertsekas, D. P. Nonlinear Programming chap. §B: “Convex Analysis”. Athena Scientific 2nd ed. (1999):.
